# Alternative and efficient one-pot three-component synthesis of substituted 2-aryl-4-styrylquinazolines/4-styrylquinazolines from synthetically available 1-(2-aminophenyl)-3-arylprop-2-en-1-ones: characterization and evaluation of their antiproliferative activities†

**DOI:** 10.1039/d4ra03702b

**Published:** 2024-07-02

**Authors:** Diego Fernando Rodríguez, Kelly Johanna Lipez, Elena Stashenko, Iván Díaz, Justo Cobo, Alirio Palma

**Affiliations:** a Laboratorio de Síntesis Orgánica, Escuela de Química, Universidad Industrial de Santander AA 678 Bucaramanga Colombia apalma@uis.edu.co; b National Research Center for the Agroindustrialization of Aromatic and Medicinal Tropical Species (CENIVAM), Universidad Industrial de Santander Colombia; c Departamento de Química Inorgánica y Orgánica, Universidad de Jaén Spain

## Abstract

In this study, an alternative and efficient one-pot three-component synthesis approach to develop a new series of (*E*)-2-aryl-4-styrylquinazolines and (*E*)-4-styrylquinazolines is described. According to this approach, the target compounds were synthesized straightforward in high yields and in short reaction times from substituted 1-(2-aminophenyl)-3-arylprop-2-en-1-ones *via* its well-Cu(OAc)_2_-mediated cyclocondensation reactions with aromatic aldehydes or its well-catalyst-free cyclocondensation reactions with trimethoxy methane (trimethyl orthoformate), and ammonium acetate under aerobic conditions. This is an operationally simple, valuable, and direct method to synthesize 2-aryl- and non-C2-substituted quinazolines containing a styryl framework at C4 position from cheap and synthetically available starting materials. All the synthesized compounds were submitted to the US National Cancer Institute for *in vitro* screening. The bromo- and chloro-substituted quinazolines 5c and 5d displayed a potent antitumor activity against all the tested subpanel tumor cell lines with IC_50_ (MG-MID) values of 5.25 and 5.50 μM, and a low cytotoxic effect with LC_50_ (MG-MID) values of 91.20 and 84.67 μM, respectively, indicating a low toxicity of these compounds to normal human cell lines, as required for potential antitumor agents.

## Abstract 2

The AcONH_4_-promoted and catalyst-free one-pot three-component synthesis of diverse substituted 4-styrylquinazolines have been developed from 2′-aminochalcones, aromatic aldehydes/trimethyl orthoformate, and ammonium acetate.

## Graphical abstract

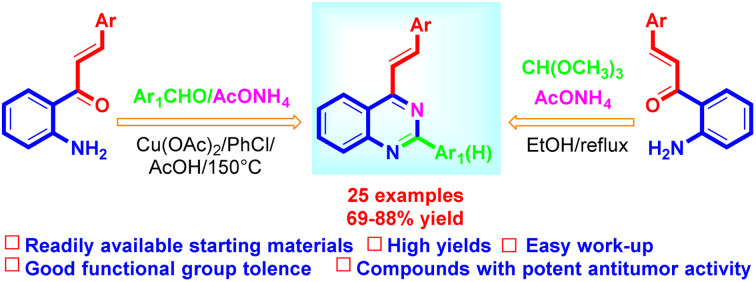

## 1 Introduction

**Fig. 1:**
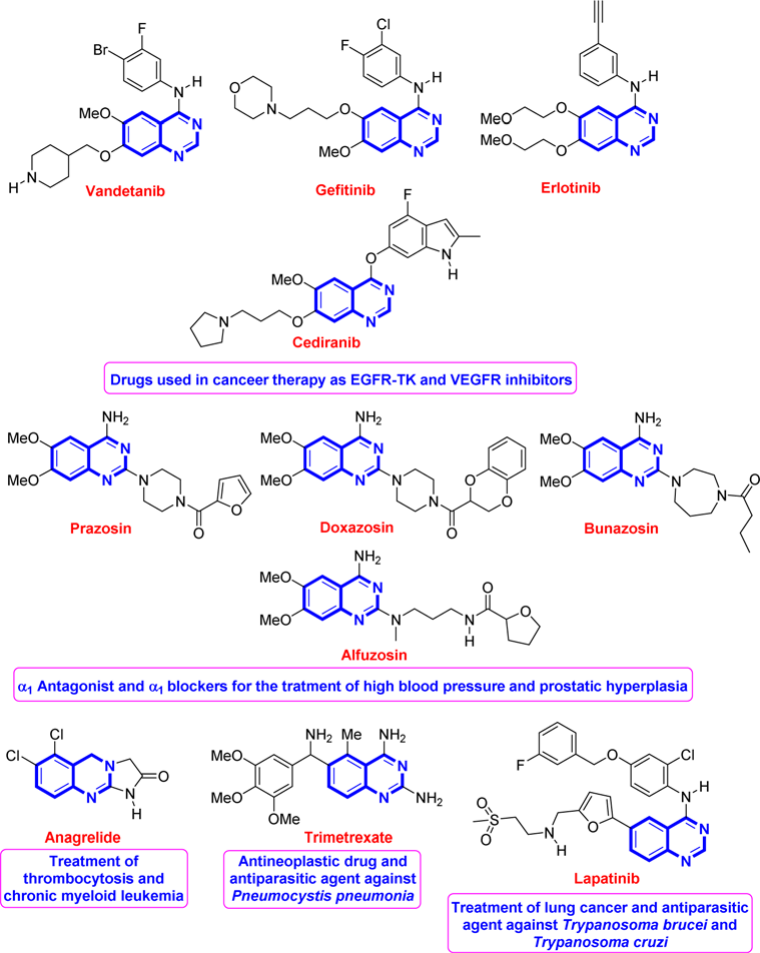
Representative drugs based on the quinazoline backbone.

Quinazoline is regarded as a privileged scaffold found in many naturally existing and synthesized compounds and used as an effective template in medicinal chemistry for drug discovery.^1^ Compounds bearing this fragment have shown remarkable and broad-spectrum biological and pharmacological activities such as antimicrobial,^2^ antipsychotic,^3^ antimalarial,^1*d*,4^ anti-spasm,^1*b*^ and anticancer^1*a*,*c*,5^ activities. Fig. 1 shows some representative examples of known quinazoline-based drugs used for the treatment of several diseases.^1*a*,*d*,5*b*,6–10^

Due to their wide-ranging biological and pharmacological properties, the synthesis of quinazoline derivatives has been extensively studied over the years. Regardless of efficiency and importance, most of the developed synthetic approaches involve either multiple steps or the use of special or not readily available starting materials or preliminary preparation of some intermediates.^11–19^ Among them, the one-pot three-component cyclocondensation reaction between 2-aminoaryl ketones or 2-aminobenzonitriles and electrophilic reagents such as aromatic aldehydes or orthoesters, and amines or ammonium acetate under different reaction conditions is the most common way towards quinazoline derivatives.^20^

**Scheme 1:**
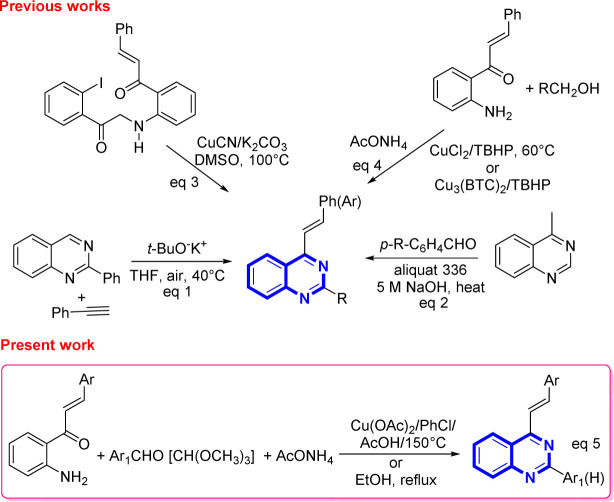
Recent approaches to the synthesis of substituted 4-styrylquinazolines.

However, despite the existence of a myriad of compounds based on the quinazoline backbone, studies related to the synthesis and biological activities of 4-styrylquinazolines have been found to be scarce in the literature so far. These types of compounds are usually prepared by employing the direct *t*-BuOK-mediated stereoselective alkenylation of preformed quinazolines with unactivated terminal alkynes (Scheme 1, eqn (1)),^21^ or by achieving the Knoevenagel-type condensation of preformed 4-methylquinazolines with aromatic aldehydes (Scheme 1, eqn (2)),^22^ or through copper-catalyzed cyanation of 2-iodo-*N*-arylbenzamides with the subsequent rearrangement of the formed 2-cyano-*N*-arylbenzamides (Scheme 1, eqn (3)).^23^

In recent years, Satish and co-workers, in 2019, and Krishnan and co-workers, in 2022, have used copper(ii) chloride or copper-based metal organic frameworks to promote the one-pot three-component oxidative amination of 2′-aminochalcones with methanol and primary alcohols and ammonium acetate in the presence of *tert*-butyl hydroperoxide affording substituted 4-styrylquinazolines (Scheme 1, eqn (4)).^24^

Our group has already reported the use of 2′-aminochalcones as valuable precursors for the straightforward synthesis of functionally diverse and structurally complex 4-styrylquinolines through the Friedländer reaction.^25^ As part of an ongoing interest on the reactivity and synthetic utility of 2′-aminochalcones, herein, we aimed to provide a simple, efficient, and alternative one-pot three-component synthesis approach of 2-aryl-4-styrylquinazolines and 4-styryl-quinazolines *via* Cu(OAc)_2_-promoted and catalyst-free cyclocondensations of 1-(2-aminophenyl)-3-arylprop-2-en-1-ones with aromatic aldehydes or trimethyl orthoformate, and ammonium acetate under aerobic conditions (Scheme 1, eqn (5)).

## 2 Results and discussion

We have started using 2′-aminochalcone 1a, benzaldehyde 2a and ammonium acetate as the reaction model to set up the conditions for the designed 2-aryl-4-styrylquinazolines and 4-styrylquinazolines. The reactants were combined in 1.0/1.0/2.5 molar ratio, respectively, and reacted under different experimental conditions, as shown in Table 1. Initially, we tried to perform the reaction in refluxing ethanol under aerobic conditions without any catalyst; however, after 6 and 20 h of heating, only multiple spots on TLC controls were observed (entries 1 and 2). To evaluate the influence of catalysts/promoters on this transformation, molecular iodine, CAN, CoCl_2_·2H_2_O, and ZnCl_2_ were tested in ethanol (entries 3–8) and toluene (entries 9 and 10), but none of them was capable of performing the planed transformation. No reaction also occurred when glacial acetic acid (AcOH) was used as the catalyst and solvent (entries 11 and 12). A new compound was observed (TLC control) when the reaction was performed in chlorobenzene at 150 °C without any addition of catalyst (entry 13). After purification of the reaction mixture by column chromatography, the new product formed was obtained as a solid in 15% yield with recovery of starting 2′-aminochalcone 1a. NMR analysis confirmed that this product was the expected 2-phenyl-4-styrylquinazoline 3a.

**Table 1:** Initial trials for the one-pot three-component synthesis of 2-phenyl-4-styrylquinazoline 3a

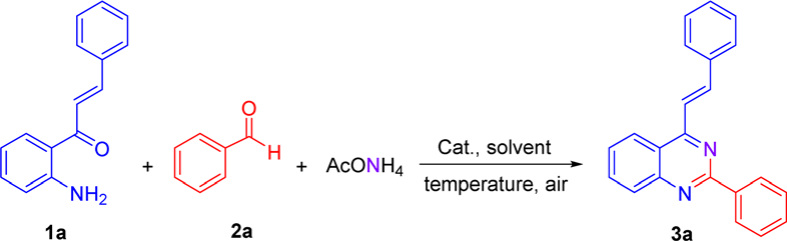					
Entry^a^	Solvent	Catalyst/additive	*T* (°C)	*t* (h)	Yield (%)
1	EtOH	—	80	6	—
2	EtOH	—	80	20	—
3	EtOH	I_2_ (5.0 mol%)	80	20	—
4	EtOH	I_2_ (10 mol%)	80	20	—
5	EtOH	CAN (5.0 mol%)	80	7	—
6	EtOH	CAN (10.0 mol%)	80	10	—
7	EtOH	CoCl_2_·2H_2_O (5.0 mol%)	80	5	—
8	EtOH	CoCl_2_·2H_2_O (10.0 mol%)	80	10	—
9	Toluene	ZnCl_2_ (5.0 mol%)	90	7	—
10	Toluene	ZnCl_2_ (10.0 mol%)	110	10	—
11	AcOH	—	100	10	—
12	AcOH	—	115	10	—
13	PhCl	—	150	10	15
14^b^	PhCl/AcOH	—	150	8	27
15^c^	PhCl/DMSO	—	150	12	14
16	PhCl/AcOH	NiCl_2_·6H_2_O (5.0 mol%)	150	8	33
17	PhCl/AcOH	NiCl_2_·6H_2_O (10.0 mol%)	150	8	43
18	PhCl/AcOH	Cu(OAc)_2_ (0.5 mol)	150	2	50
19	PhCl/AcOH	Cu(OAc)_2_ (0.5 mmol)	120	4	36
20	PhCl/AcOH	Cu(OAc)_2_ (1.0 mmol)	150	1	60
21	PhCl	Cu(OAc)_2_ (1.0 mmol)	150	5	48
22^d^	PhCl/AcOH	Cu(OAc)_2_ (1.0 mmol)	150	2	60
23^e^	PhCl/AcOH	Cu(OAc)_2_ (1.0 mmol)	150	2	62
**24** b	**PhCl/AcOH**	**Cu(OAc)**_**2**_ **(1.3 mmol)**	**150**	**1**	**81**
25	PhCl/AcOH	Cu(OAc)_2_ (1.5 mmol)	150	4	72
26^f^	PhCl/AcOH	Cu(OAc)_2_ (1.3 mmol)	150	3	68
27^g^	PhCl/AcOH	Cu(OAc)_2_ (1.3 mmol)	150	1	80
28^h^	PhCl/AcOH	Cu(OAc)_2_ (1.3 mmol)	150	1	—
29^i^	PhCl/AcOH	Cu(OAc)_2_ (1.3 mmol)	150	1	—
30^j^	PhCl/AcOH	Cu(OAc)_2_ (1.3 mmol)	150	1	—

a 1a (1.0 mmol), 2a (1.0 mmol), AcONH_4_ (2.5 mmol).

b PhCl (2 mL)/AcOH (three drops).

c PhCl (2 mL)/DMSO (5 mol%).

d PhCl (2 mL)/AcOH (0.5 mL).

e PhCl (2 mL)/AcOH (1.0 mL).

f AcONH_4_ (2.0 mmol).

g AcONH_4_ (3.0 mmol).

h NH_4_I (2.5 mmol).

i NH_4_Cl (2.5 mmol).

j (NH_4_)_2_SO_4_ (2.5 mmol).

Notably, the yield of 3a practically doubled and the reaction time decreased in 2 h when AcOH (three drops) was added to the reaction mixture (entry 14). By contrast, introduction of an aprotic polar solvent such as DMSO to the reaction mixture had a negative effect and delivered 3a in diminished yields (entry 15).

The above-mentioned experimental results showed that chlorobenzene as the solvent with a little amount of AcOH is the best basic medium to perform our model reaction, and we started combining with other catalysts/promoters. Therefore, we decided to evaluate the effect of NiCl_2_·6H_2_O (5.0–10.0 mol%) and Cu(OAc)_2_ (0.5–1.0 mmol), running the reaction under aerobic conditions at 150 °C. We were pleased to find that the presence of these two salts has a significant influence on the 3a yields (entries 16–20). Among these two salts, Cu(OAc)_2_, in an equimolar ratio, was found to be the best in promoting the reaction, whereby 3a was isolated in 60% yield after 1 h of heating (entry 20).

In this optimization process, it was also observed that a decrease in the amount of Cu(OAc)_2_ and the temperature led to a drop in 3a yield (entries 18 and 19). Without addition of AcOH, a significant reduction in the yield was also noticed (entry 21). Furthermore, an increase in the amount of AcOH had only a negligible effect on the reaction outcome, giving 3a in similar yields to that obtained in entry 20 (entries 22 and 23). These results strongly suggested that equimolar amounts of Cu(OAc)_2_ in combination with a catalytic amount of AcOH in chlorobenzene play an important role in this one-pot three-component transformation.

Encouraged by these results, we moved then to further optimize the reaction yield by evaluating the effect of temperature, reaction time and reactant/Cu(OAc)_2_ molar ratios. Pleasingly, there was a significant improvement in 3a yield when the reaction was performed using 1.3 mmol of Cu(OAc)_2_ at 150 °C for 1 h without changing the initial molar ratios of reactants (1.0/1.0/2.5); under these conditions, the reaction was very clean and provided the desired product in 81% yield (entry 24). On the contrary, a significant drop in 3a yield was noticed when the amount of Cu(OAc)_2_ was increased to 1.5 mmol (entry 25), but especially when the amount of AcONH_4_ was decreased to 2.0 mmol (entry 26). Additionally, increasing the amount of AcONH_4_ from 2.5 to 3.0 mmol did not afford better results (entry 27), giving 3a in a similar yield to that obtained in entry 24. Finally, when the model reaction was performed in the fixed molar ratios of reactants and conditions of entry 24, but using other ammonia sources such as NH_4_I, NH_4_Cl and (NH_4_)_2_SO_4_ instead of AcONH_4_, in each case, a complex mixture of compounds was noticed (entries 28–30). From these trials, it was concluded that the best conditions to perform this one-pot three-component transformation are those highlighted in entry 24 (Table 1).

**Scheme 2:**
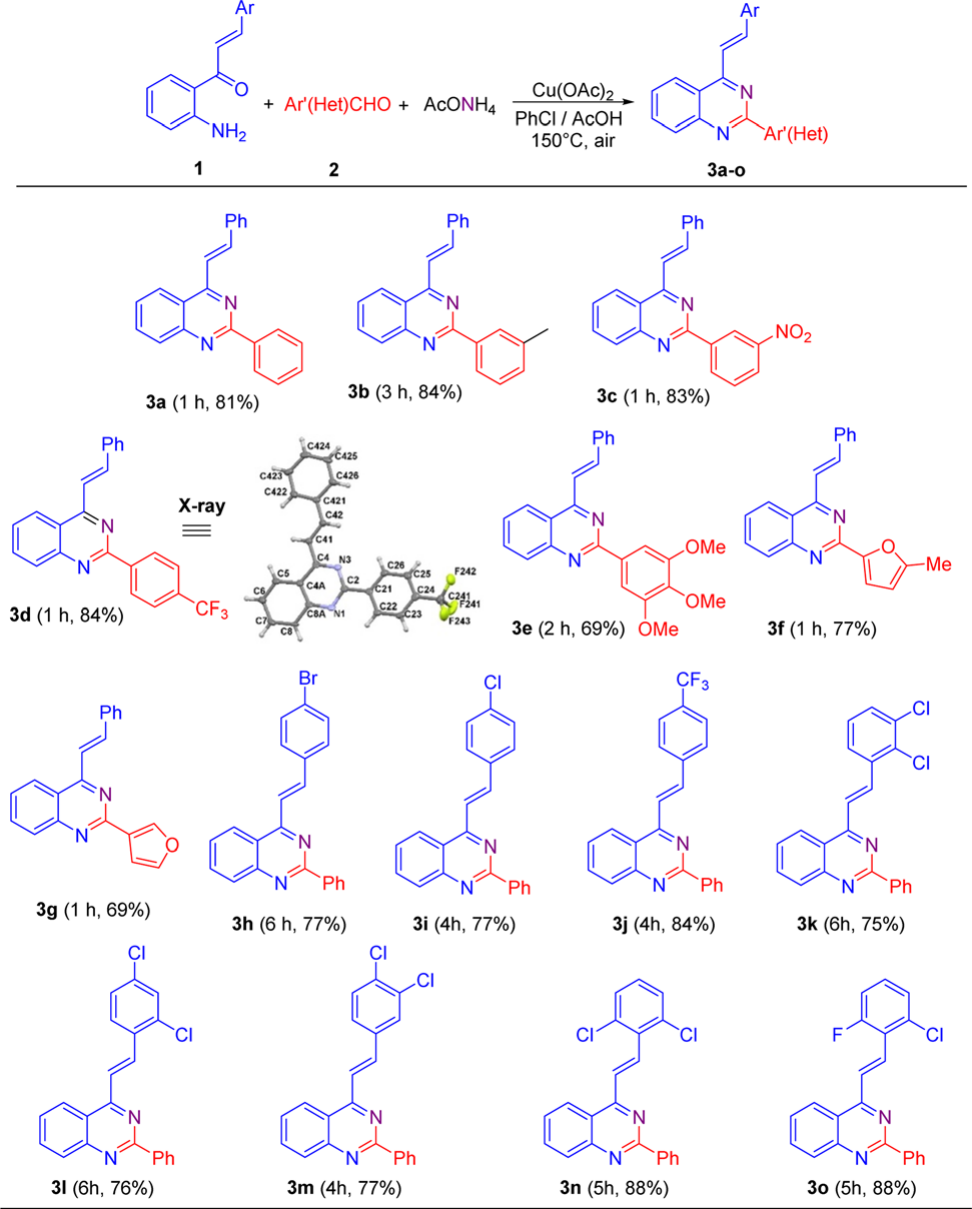
Cu(AcO)_2_-promoted oxidative cyclocondensation of 2′-aminochalcones 1 with aromatic aldehydes 2 and ammonium acetate under aerobic conditions. The X-ray structure for 3d is shown with thermal anisotropic ellipsoids at 50% probability. Experimental conditions: 1 (1.0 mmol), 2 (1.0 mmol), AcONH_4_ (2.5 mmol), Cu(OAc)_2_ (1.3 mmol), PhCl (2 mL), and AcOH (three drops).

Once the reaction conditions were established, they were applied to different substrates to explore the scope of this transformation. Initially, we studied the behavior of 2′-aminochalcone 1a in its reaction with (hetero)aromatic aldehydes 2 (Scheme 2). Pleasingly, it was found that all the tested aldehydes reacted well with 1a to produce the corresponding 2-aryl-4-styrylquinazolines 3 in good to excellent yields.

As shown in Scheme 2, benzaldehydes containing a weak electron-donating group at *m*eta position (2b) or strong electron-withdrawing substituents at *m*eta (2c) and *para* (2d) positions practically did not affect the reaction yields, affording the expected 4-styryl-2-arylquinazolines 3b–3d in yields comparable to that obtained for 3a. Nevertheless, the *meta*-methylbenzaldehyde 2b exhibited a lower reactivity than that of its *meta*-nitro- and *para*-trifluoromethyl-substituted analogues 2c and 2d, and so a longer reaction time was necessary for substrate consumption.

Similarly, trisubstituted benzaldehydes such as 3,4,5-trimethoxybenzaldehyde 2e and heteroaromatic aldehydes such as 5-methylfuran-2-carbaldehyde 2f and furan-3-carbaldehyde 2g proceeded successfully to come up with the expected products. However, in general, they exhibited lower efficiencies than their 2a–2d analogues and afforded the corresponding quinazoline derivatives 3e–3g in lower yields.

Next, we evaluated the reactivity of 2′-aminochalcones 1 containing one or two electron-withdrawing substituents at different positions on the benzene ring of the styryl fragment with benzaldehyde 2a as the fixed electrophilic partner. It was found that both *para*-monosubstituted (1h–1j) and disubstituted (1k–1o) chalcones were successfully subjected to this transformation but required longer reaction times to afford the corresponding 2-aryl-4-styrylquinazolines 3h–3o in yields of 75–88%. 2′-Aminochalcone with a trifluoromethyl group at the *para*-position 1j gave a better yield (84%) than that of its *para*-halogen-substituted analogues 1h (77%) and 1i (77%). The reaction with dichloro-substituted chalcones 1k–1m also reached completion within 4–6 h, producing the desired quinazolines 3k–3m in similar yields to that obtained for *p*-bromo(chloro)-substituted analogues 3h and 3i.

Among the tested substituted 2′-aminochalcones 1, 2,6-dichloro- (1n) and 2-chloro-6-fluoro- (1o) disubstituted derivatives afforded the corresponding 2-phenyl-4-styrylquinazolines 3n and 3o in the best yields (88%).

To broaden the scope of this one-pot three-component transformation, we then evaluated the reaction of 2′-aminochalcones 1 with ammonium acetate and trimethyl orthoformate 4 instead of aromatic aldehydes. Curiously, to the best of our knowledge, this transformation was not previously explored with the aim of assembling the non-substituted C2 4-styrylquinazoline core position.

As shown in Table 2, this one-pot three-component cyclocondensation under the optimized conditions, using 1a as the model substrate, occurs at a much lower rate than that of their 2-aryl-4-styrylquinazoline analogues 3, affording the expected 4-styrylquinazoline 5a in only 45% yield after 20 h of heating (entry 1). This unsatisfactory result prompted us to investigate again this transformation in more detail with respect to Cu(OAc)_2_ loading, molar ratios of reactants, solvent, and temperature.

**Table 2:** Optimization of the 4-styrylquinazoline 5a synthesis from 2′-aminochalcone 1a, trimethyl orthoformate 4 and ammonium acetate

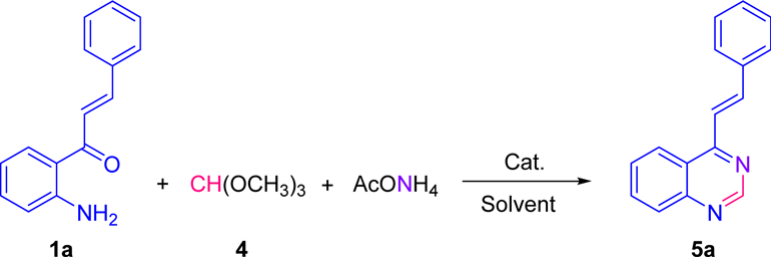						
Entry	Ratio	Solvent	Cu(OAc)_2_ (mmol)	*T* (°C)	*t* (h)	Yield (%)
	1a/4/AcONH_4_					
1	1.0 : 1.0 : 2.5	PhCl/AcOH	1.3 mmol	150	20	45
2	1.0 : 2.0 : 2.5	PhCl/AcOH	1.3 mmol	150	20	47
3	1.0 : 3.5 : 2.5	PhCl/AcOH	1.3 mmol	150	20	50
4	1.0 : 3.5 : 2.5	PhCl/AcOH	1.0 mmol	150	18	55
5	1.0 : 3.5 : 2.5	PhCl/AcOH	0.5 mmol	150	18	58
6	1.0 : 3.5 : 2.5	PhCl/AcOH	0.5 mmol	100	24	60
7	1.0 : 3.5 : 2.5	PhCl	—	150	18	55
8	1.0 : 3.5 : 2.5	PhCl	—	100	18	50
9	1.0 : 3.5 : 2.5	AcOH	—	110	10	—
10	1.0 : 3.5 : 2.5	AcOH	—	80	10	—
11	1.0 : 3.5 : 2.5	—	—	80	15	—
12	1.0 : 3.5 : 2.5	—	—	120	15	—
13	1.0 : 3.5 : 2.5	MeOH	—	65	25	63
14	1.0 : 3.5 : 2.5	EtOH	—	80	18	70
**15**	**1.0 : 3.5 : 3.0**	**EtOH**	**—**	**80**	**5**	**85**
16	1.0 : 3.0 : 3.0	EtOH	—	80	8	80
17	1.0 : 4.0 : 3.5	EtOH	—	80	4	83
18	1.0 : 4.0 : 3.5	EtOH	—	80	10	84
19	1.0 : 4.0 : 4.0	EtOH	—	80	10	82
20	1.0 : 3.5 : 2.5	*n*-BuOH	—	120	17	67

In this new optimization process, it was observed that an increase in the amount of 4 from 1.0 to 2.0–3.5 mmol without changing the reaction temperature and the molar ratio of Cu(OAc)_2_, 1a, and ammonium acetate had only a negligible influence on the 5a yields (entries 2 and 3).

Nevertheless, the yield of 5a was significantly improved (up to 60%) running the reaction with 3.5 mmol of 4 and reducing, at the same time, both the amount of Cu(OAc)_2_ and the reaction temperature (entries 4–6). Similarly, performing the reaction using 3.5 mmol of 4 in PhCl at 150 and 100 °C in the absence of Cu(OAc)_2_ and AcOH had a little effect on the reaction outcome and delivered 3a in diminished yields (entries 7 and 8). The desired product was not formed at all when AcOH was used as the catalyst and solvent (entries 9 and 10).

It would appear from these results that both Cu(OAc)_2_ and AcOH are not necessary for the reaction to take place. Contrary to the report by Bath and coworkers,^20*a*^ we found that the solvent nature plays an important role in this transformation. For example, the solvent-free trials at 80 and 120 °C did not afford the expected compound (entries 11 and 12).

In the light of the above-mentioned results, we decided to perform the planed transformation by simple heating in different polar solvents (methanol, ethanol, and *n*-butanol), evaluating the effect of reaction times and molar ratio of reactants (entries 13–20). The reaction proceeded in all three tested solvents, with ethanol as the best one/solvent for this transformation (entries 14–19). With ethanol as the solvent, the optimal molar ratio of reactants for the reaction was determined to be 1.0/3.5/3.0 (1a : 4 : AcONH_4_); under these conditions, the reaction reached completion within 5 h, affording the desired product 5a in 85% yield (entry 15). Other molar ratios of reactants did not afford better results even if the reaction time was extended to 8–18 h (entries 14 and 16–19).

**Scheme 3:**
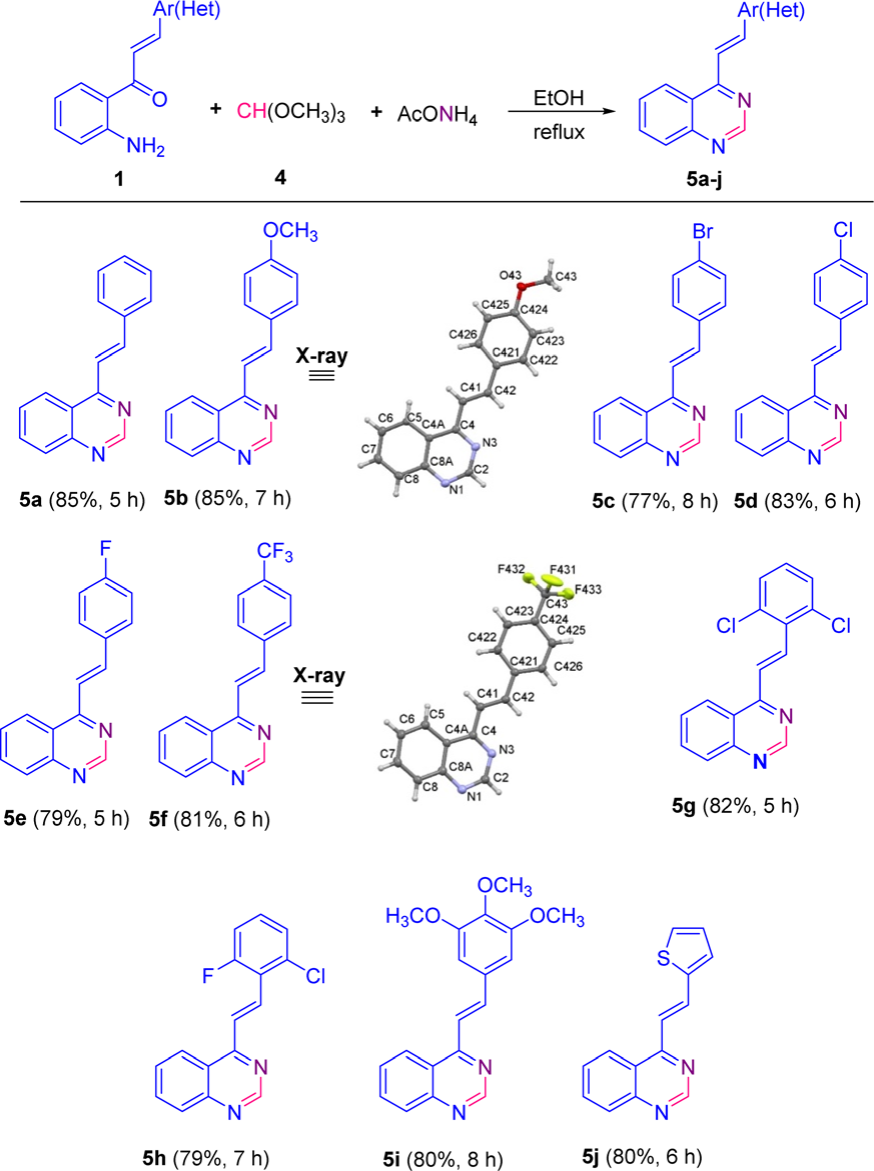
Catalyst-free one-pot three-component synthesis of 4-styrylquinazolines 5. The X-ray structures of 5b and 5f are shown with thermal anisotropic ellipsoids at 50% probability.

The scope of this catalyst-free one-pot three-component reaction with different substituted 2′-aminochalcones 1 was explored under the optimized conditions highlighted in entry 15, and the results are listed in Scheme 3. It was rewarding to find that chalcones 1a–i containing substituents on the aromatic ring of the styryl fragment, regarding their electronic behavior, that is, both electron donating/withdrawing groups, and their substitution pattern (mono, di- or trisubstituted) were also successfully subjected to this transformation. As a result, the corresponding 4-styrylquinazoline derivatives 5a–i were obtained in similar yields to that obtained for 5a, regardless of the positions of substituents.

Among the *para*-halogenated 2′-aminochalcones 1c, 1d, and 1e, and *para*-trifluoromethyl-substituted chalcone 1f, chalcones 1d and 1f resulted to be the most reactive, giving the corresponding 4-styrylquinazolines 5d and 5f in yields of 83 and 81%, respectively. The *para*-bromo- (1c) and the *para*-fluoro- (1e) substituted 2′-aminochalcones also worked well in this transformation, though providing the 4-styrylquinazolines 5c and 5e in slightly inferior yields (77 and 79%, respectively).

No significant differences were noticed in the efficiencies of dihalogenated chalcones 1g and 1h with respect to trimethoxy-substituted chalcone 1i; all three chalcones successfully produced the expected quinazolines 5g–5i in yields of 82, 79 and 80%, respectively. Moreover, 2′-aminochalcone 1j containing a thiophene ring in the vinyl fragment instead of the benzene ring also participated smoothly in this transformation, providing the corresponding quinazoline 5j in a similar yield (80%).

Among all the tested 2′-aminochalcones 1, the non-substituted and *para*-methoxy-substituted chalcones 1a and 1b showed the highest efficiencies and provided the corresponding 4-styrylquinazolines 5a and 5b in the best yields (85%).

Finally, we also tried to use triethyl orthoacetate in reaction with 2′-aminochalcone 1a and ammonium acetate to assemble the 2-methyl-substituted 4-styrylquinazoline core. The importance of this class of derivatives lies in their potential use as building blocks of more complex molecules due to the inherent reactivity of the methyl group that allows for further functionalization. Unfortunately, under the reaction conditions established for compounds 3 and 5, the planned one-pot three-component cyclocondensation with triethyl orthoacetate did not occur. This may be due to its lower reactivity compared to that shown by trimethyl orthoformate and aromatic aldehydes.

The structures of the target compounds 3 and 5 were established mainly by a combined study of IR, HRMS and ^1^H NMR and ^13^C NMR spectra (see the Experimental section). In their IR spectra, the absence of any N–H stretching bands around 3275–3285 cm^−1^, which are characteristic in the spectra of 2′-aminochalcone precursors 1, was used for monitoring the formation of the quinazoline ring. Additionally, the IR spectra of 3 and 5 showed the characteristic absorption band at 961–973 cm^−1^ (for 3) and 948–983 cm^−1^ (for 5) attributed to a *trans*-disubstituted alkene. Additional supports for the expected structures were obtained from their mass-spectra. Compounds were identified based on the elemental composition of the protonated molecules and their product ions [M + H]^+^, which confirmed their exact molecular weights (see Experimental section).

**Scheme 4:**
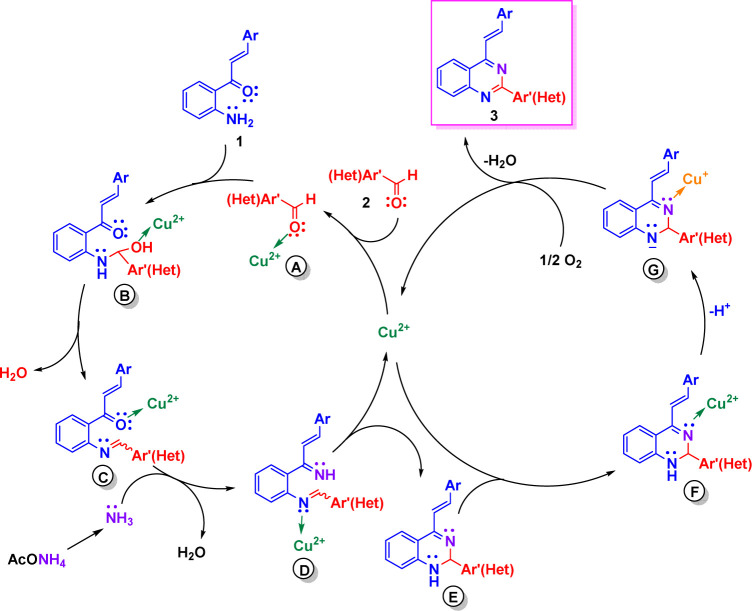
Plausible mechanism leading to 2-aryl-4-styrylquinazolines 3.

Based on literature reports,^17,20*a*,*e*^ we propose tentative reaction mechanisms by which the formation of 2-aryl-4-styrylquinazolines 3 and 4-styrylquinazolines 5 could occur. The rationalization of mechanism for compounds 3 is depicted in Scheme 4. First, the carbonyl group of aromatic aldehydes 2 is activated by Cu(ii) to give complex A. Nucleophilic attack of 2′-aminochalcone 1 onto activated aldehyde A followed by dehydration of the formed aminol B yields the imine intermediate C in which the carbonyl group is activated by Cu(ii). Subsequent condensation of C with ammonia, generated from ammonium acetate, produces the diimine D, which upon intramolecular cycloaddition and release of Cu(ii) afforded the dihydroquinazoline E. Then, Cu(ii) coordinates with the imine nitrogen atom of E and produces the intermediate F, which upon deprotonation and reduction of Cu(ii) to Cu(i) is transformed into the anion G.^17^ Finally, after reoxidation of the Cu(i) complex to the Cu(ii) complex, compounds 3 are released and the catalytic cycle would be closed.

**Scheme 5:**
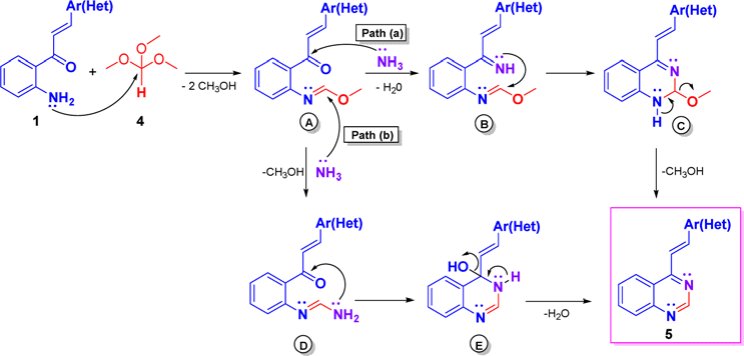
Plausible mechanism leading to 4-styrylquinazolines 5.

The plausible mechanism for compounds 5 is illustrated in Scheme 5, which could imply the initial formation of methyl formimidate A, which is transformed into the final product 5*via* two possible paths.^20*a*^ Through path a, intermediate A by condensation with ammonia, generated from ammonium acetate, yields the methyl imino-formimidate B, which upon intramolecular cycloaddition produces the dihydroquinazoline C. Finally, C is transformed into the target compound 5 by loss of a molecule of methanol. Through path b, intermediate A reacts with ammonia *via* nucleophilic substitution affording the formimidamide D, which, in turn, upon intramolecular cycloaddition and subsequent dehydration of the formed dihydroquinazolin-4-ol E yields the target 4-styryquinazoline 5.

The formation of the 4-styrylquinazoline scaffolds with 2-aryl-substituted and non-substituted at the C2 position was unambiguously confirmed by a detailed analysis of the ^1^H, ^13^C and 2D NMR spectra, which showed no signals arising from the H atoms of the amino group, neither were there any signals from the carbonyl group which had been present in the precursor chalcones 1. Instead, the ^13^C spectra of quinazolines 3 contained signals from two new quaternary aromatic C atoms at *δ* 153.7–160.4 (C2) and *δ* 161.1–162.3 (C4). In turn, the ^1^H and ^13^C spectra of the non-substituted at C2 styrylquinazolines 5 contained signals from a new Caryl-H unit (H2/C2) at *δ* 9.23–9.36 (singlet) and *δ* 153.6–155.0, and a new quaternary aromatic C atom at *δ* 161.4–162.4 (C4). As in the spectra of the precursor chalcones 1, the ^1^H spectra of 3 and 5 contained signals from the *trans* vinylic protons –CH_A_=CH_B_–, appearing as two doublets in the aromatic region at *δ* 7.91–8.29 and 8.32–8.80 (for 3) and at *δ* 7.69–8.25 and 8.21–8.48 (for 5), respectively.

Additionally, the molecular structures of representative compounds 3d, 5b and 5f were unambiguously established by single-crystal X-ray diffraction analysis, and their structures are included in Schemes 2 and 3. Their X-ray crystal structures agree with the structural elucidation deduced from NMR analysis.

All the synthesized 2-aryl-4-styrylquinazolines 3 (15 compounds) and 4-styrylquinazolines 5 (10 compounds) were selected by the USA National Cancer Institute (NCI) to test their antitumor activities under the drug discovery program of the NCI (USA).^26^ Two of them showed an average inhibition percentage higher than 70%, which corresponding to *para*-bromostyryl- (5c) (NSC 840315) and *para*-chlorostyryl- (5d) (NSC 847835) substituted quinazolines were so selected to determine their IC_50_ and LC_50_ at five concentrations at 10-fold dilution (100, 10, 1, 0.1 and 0.01 μM). The other twenty-three compounds proved to have limited activity or were totally inactive (see Tables 1S and 2S in the ESI†).

An overview of the substitution pattern of the tested compounds reveals that active compounds 5c and 5d belong to the non-substituted at C2 position 4-styryquinazolines, suggesting that the presence of a phenyl or aryl substituent at C2 position has a detrimental effect on the antitumor activity.

The results obtained in this assay indicated that the two compounds under investigation (5c and 5d) exhibited remarkable antitumor activities against most of the tested subpanel tumor cell lines. Overall, both compounds showed practically the same pattern of sensitivity against some individual cell lines (Table 3), as well as a broad spectrum of antitumor activity (Table 4). Regarding the sensitivity against some individual cell lines, 5c and 5d proved to be very sensitive towards most of the tested subpanel tumor cell lines with IC_50_ values in the range of 1.78–31.62 μM. However, 5d displayed the best sensitivity profile against all the examined cancer cell lines with IC_50_ values in the range of 1.78–28.84 μM (Table 3). For these two compounds, the most sensitive cell lines are as follows: SNB-75 (CNS cancer, IC_50_ = 2.24 μM, for 5c), HCT-15 (colon cancer, IC_50_ = 1.78 μM, for 5d), SNB-75 (CNS cancer, IC_50_ = 1.91 μM, for 5d), and MCF7 and MDA-MB-468 (breast cancer, IC_50_ = 1.86 μM, for 5d, and IC_50_ = 2.09 and 1.91 μM, for 5c and 5d, respectively).

**Table 3:** Inhibitory concentration (IC_50_, μM) and lethal concentration (LC_50_, μM) of compounds 5c and 5d over all tested *in vitro* tumor cell lines^a^

Cancer panel cell line	Compound			
	5c b		5d c	
	IC_50_ (μM)	LC_50_ (μM)	IC_50_ (μM)	LC_50_ (μM)
**Leukemia (I)**				
CCRF-CEM	3.09	>100	3.72	>100
HL-60(TB)	2.45	>100	2.40	>100
K-562	3.24	>100	4.07	>100
MOLT-4	3.89	>100	12.02	>100
RPMI-8226	3.24	>100	NT^d^	NT
SR	3.24	>100	NT	NT

**Non-small cell lung cancer (II)**				
A549/ATCC	6.92	>100	5.75	>100
EKVX	2.95	>100	2.63	>100
HOP-62	5.50	>100	10.47	>100
NCI-H226	7.76	93.33	2.82	>100
NCI-H23	3.72	63.10	3.16	70.79
NCI-H322M	4.37	>100	3.47	69.18
NCI-H460	5.75	>100	8.51	>100
NCI-H522	4.79	77.62	10.72	>100

**Colon cancer (III)**				
COLO 205	5.62	45.71	3.72	43.65
HCC-2998	5.01	81.28	6.61	52.48
HCT-116	3.39	>100	3.72	52.48
HCT-15	2.51	>100	1.78	63.10
HT29	5.75	>100	5.37	63.10
KM12	3.02	>100	10.23	57.54
SW-620	3.16	>100	3.55	>100

**CNS cancer (IV)**				
SF-268	7.76	>100	9.12	>100
SF-295	14.79	>100	3.80	>100
SF-539	5.75	60.26	4.90	46.77
SNB-19	7.24	>100	10.72	52.48
SNB-75	2.24	>100	1.91	>100
U251	4.79	>100	4.37	64.57

**Melanoma (V)**				
LOX IMVI	3.63	41.69	4.17	47.86
MALME-3M	15.49	>100	22.91	>100
M14	6.17	>100	5.13	56.23
MDA-MB-435	4.07	>100	4.90	>100
SK-MEL-2	5.50	89.13	6.92	56.23
SK-MEL-28	13.18	>100	11.22	64.57
SK-MEL-5	9.33	54.95	10.47	47.86
UACC-257	31.62	>100	7.76	>100
UACC-62	5.25	58.88	4.47	58.88

**Ovarian cancer (VI)**				
IGROV1	17.38	>100	15.85	>100
OVCAR-3	2.51	74.13	3.47	>100
OVCAR-4	19.05	>100	10.23	>100
OVCAR-5	5.01	>100	4.57	>100
OVCAR-8	5.89	>100	5.50	97.72
NCI/ADR-RES	2.82	100.00	2.34	75.86
SK-OV-3	10.47	>100	5.50	>100

**Renal cancer (VII)**				
786-0	12.88	>100	8.51	>100
A498	16.60	>100	28.84	>100
ACHN	5.13	>100	4.57	>100
CAKI-1	3.63	>100	3.98	>100
RXF 393	4.79	>100	ND	NT
SN12C	3.72	>100	5.50	61.66
TK-10	19.05	>100	19.05	>100
UO-31	3.47	>100	4.37	>100

**Prostate cancer (VIII)**				
PC-3	5.62	>100	5.89	>100
DU-145	4.37	>100	6.03	>100

**Breast cancer (IX)**				
MCF7	2.45	>100	1.86	56.23
MDA-MB-231/ATCC	4.27	>100	11.75	>100
HS 578T	8.13	>100	9.33	>100
BT-549	3.09	75.86	2.63	44.67
T-47D	2.75	>100	2.69	>100
MDA-MB-468	2.09	32.36	1.91	27.54

a Data obtained from NCIs *in vitro* disease-oriented human tumor cell screen.

b NSC 840315.

c NSC 847835.

d NT, not tested.

**Table 4:** Median 50% inhibitory concentration (IC_50_, μM) values of *in vitro* subpanel tumor cell lines

Comp.	Subpanel tumor cell lines^a^									MG-MID^b^
	I	II	III	IV	V	VI	VII	VIII	IX	
5c	3.19	5.22	4.07	7.10	10.47	9.02	8.66	4.99	3.80	**5.25**
5d	5.55	5.68	5.00	5.80	8.66	6.78	10.69	5.96	5.03	**5.50**

a I = leukemia; II = non-small cell lung cancer; III = colon cancer; IV = CNS cancer; V = melanoma; VI = ovarian cancer; VII = renal cancer; VIII = prostate cancer; IX = breast cancer.

b IC_50_ (μM) full panel mean graph midpoint (MG-MID) = the average sensitivity of all cell lines towards the test compound.

Regarding the broad spectrum of antitumor activity, the results also revealed that 5c and 5d showed effective growth inhibition with IC_50_ (MG-MID) values of 5.25 and 5.50 μM (Table 4), and a low cytotoxic effect with LC_50_ (MG-MID) values of 91.20 and 84.67 μM, respectively (Table 5), indicating the low toxicity of these compounds to normal human cell lines, as required for potential antitumor agents.

**Table 5:** Median 50% lethal concentration (LC_50_, μM) values of *in vitro* subpanel tumor cell lines

Comp.	Subpanel tumor cell lines^a^									MG-MID^b^
	I	II	III	IV	V	VI	VII	VIII	IX	
5c	> 100	91.76	89.57	93.38	82.74	95.69	> 100	> 100	91.95	**91.20**
5d	> 100	87.66	61.76	77.30	73.16	96.23	94.52	> 100	71.41	**84.67**

a I = leukemia; II = non-small cell lung cancer; III = colon cancer; IV = CNS cancer; V = melanoma; VI = ovarian cancer; VII = renal cancer; VIII = prostate cancer; IX = breast cancer.

b LC_50_ (μM) full panel mean graph midpoint (MG-MID) = the average sensitivity of all cell lines towards the test compound.

## 3 Conclusions

We have described an easy and efficient one-pot three-component synthetic protocol to afford, in high yields and short reaction times, 2-aryl-4-styrylquinolines and 4-styrylquinazolines from readily available 2′-aminochalcones. Easily available and cheap starting materials, operational simplicity, high yields, and a good functional group tolerance are the most significant features of this protocol, which make it a good alternative to previous methods for the synthesis of 2-aryl- and non-C2-substituted 4-styrylquinazolines.

Additionally, in contrast to other methodologies, the synthesis of non-C2-substituted 4-styrylquinazolines did not require the use of any catalyst. Quinazolines 3 and 5 were screened for their antitumor activity, with 4-bromostyrylquinazoline 5c and 4-chloro-styrylquinazoline 5d as the most active compounds with an IC_50_ value of 5 μM. They exhibited remarkable inhibitory action against all the tested subpanel tumor cell lines and a low cytotoxic profile; thus, they can be considered promising hits, especially bearing in mind their small size and potential for further structural modifications with the aim to improve both the potency and selectivity.

## 4 Experimental

All reagents and solvents were purchased from commercial sources and used without further purification. Monitoring of the reactions was performed using silica gel TLC plates (Merck silica gel 60 F254). Spots were visualized by UV light at 254 and 365 nm. Column chromatography was performed using Merck Kieselgel (230–400 mesh) (ASTM). Melting points were determined using a MEL-TEMP 1201D capillary apparatus and are uncorrected. IR spectra were recorded using a Bruker Tensor 27 spectrometer (equipped with a platinum ATR cell). ^1^H and ^13^C NMR spectra were recorded at 25 °C using a Bruker Avance III 400 spectrometer, with CDCl_3_ as the solvent. Chemical shifts (*δ*) and coupling constant (*J*) values are reported in ppm and Hz, respectively. Chemical shifts are relative to the solvent peak used as reference (CDCl_3_: *δ* 7.26 for ^1^H and *δ* 77.23 for ^13^C). NMR multiplicity assignments: q = quartet, t = triplet, d = doublet, s = singlet, br = broad, m = multiplet. High-resolution mass spectra (HRMS) were recorded using either a Waters Micromass AutoSpect NT system (equipped with a direct inlet probe) operating at 70 eV or a Dionex Ultimate® 3000 chromatograph coupled with a LTQ Orbitrap XL™ Hybrid Ion Trap-Orbitrap Fourier Transform Mass Spectrometer with injection into HESI II in the positive mode. Elemental analyses were performed using a Vario Micro cube CHN/S analyzer and were within ±0.4 of theoretical values.

The single-crystal X-ray diffraction measurements of the suitable crystal of 3d, 5b and 5f were obtained using a Hampton Research Mounted CryoloopTM equipped with a Diffractometer Bruker D8 Venture (APEX 4) at Centro de Instrumentación Científico y Técnico” (CICT) in Universidad de Jaén (UJA), Spain, with a monochromator multilayer mirror, CCD rotation images, thick slices φ & θ scans, and a Mo INCOATEC high brilliance microfocus sealed tube as the X-ray source (*λ* = 0.71073 Å). The structures were solved by a direct method and refined by the full-matrix least-squares technique against F^2^ in the isotropic-anisotropic approximation. Data collection: APEX4 v2021.10-0;80 (ref. 27) cell refinement: SAINT V8.40B;^28^ data reduction: SAINT V8.37A;16 program(s) used to solve structure: SHELXT-2014/5; 81 (ref. 29) program(s) used to refine structure: SHELXL-2019/1;82 (ref. 30) software used to prepare material for publication: Wingx 2018.283 (ref. 31) and Mercury 3.10.3.84.^32^

It is important to mention that all compounds described in this paper were obtained as perfectly stable solid substances that could be stored without special precautions; however, some of them have been previously synthesized and reported as viscous liquids.^24*a*^

### 4.1 Synthesis of substituted (*E*)-4-styrylquinazolines 3 and 5

All reactions were performed using a Radley Carousel 12 PlusTM Reaction Station System with a stand, a stirring hotplate and an integral temperature controller.

#### 4.1.1 General procedure for the synthesis of (*E*)-2-aryl-4-styrylquinazolines 3

In a 20 mL Radleys reaction tube, 2′-aminochalcones 1 (1.0 mmol), (hetero)aromatic aldehydes 2 (1.0 mmol), ammonium acetate (2.5 mmol) and copper acetate (1.3 mmol) were mixed in chlorobenzene (2.0 mL) and glacial acetic acid (three drops) at room temperature and then capped. The obtained suspensions were stirred magnetically at 150 °C until completion of the reactions (TLC monitored). Then, the crude mixtures were allowed to cool down to room temperature and extracted with dichloromethane (3 × 30 mL) followed by washing with water (50 mL) and brine. In each case, the combined organic layers were dried over anhydrous sodium sulfate, and the solvents removed under reduced pressure. The resulting crude was purified by flash chromatography on silica gel using *n*-heptane–ethyl acetate mixtures as the eluent (10 : 1 to 8 : 1) to obtain the target compounds 3 as solid substances.

##### 4.1.1.1 (*E*)-2-Phenyl-4-styrylquinazoline (3a)

White solid; yield: 0.250 g (81%); m.p. 140–142 °C; *R*_f_ = 0.53 (9% ethyl acetate–*n*-heptane).

IR (ATR): *ν̄*_max_ (cm^−1^) 3026 (C_sp^2^_–H), 1628 (C=N), 1528 (C=C_arom_), 973 (=C–H_*trans*_).

NMR ^1^H (400 MHz, CDCl_3_): *δ* = 8.74–8.71 (m, 2H, H2′/H6′), 8.46 (d, *J* = 15.5 Hz, 1H, =CH_B_), 8.30 (dd, *J* = 8.3, 1.4 Hz, 1H, H5), 8.10 (dd, *J* = 8.4, 1.2 Hz, 1H, H8), 7.97 (d, *J* = 15.5 Hz, 1H, H_A_C=), 7.87 (ddd, *J* = 8.4, 6.9, 1.4 Hz, 1H, H7), 7.79–7.76 (m, 2H, H2/H6′′), 7.61 (ddd, *J* = 8.3, 6.9, 1.3 Hz, 1H, H6), 7.60–7.55 (m, 2H, H3′/H5′), 7.55–7.50 (m, 1H, H4′), 7.49–7.45 (m, 2H, H3′′/H5′′), 7.44–7.39 (m, 1H, H4′′).

NMR ^13^C (100 MHz, CDCl_3_): *δ* = 161.9 (C4), 160.2 (C2), 152.1 (C8a), 139.5 (=CH_B_), 138.6 (C1′), 136.2 (C1′′), 133.5 (C7), 130.4 (C4′), 129.6 (C4′′), 129.4 (C8), 128.9 (C3′′/C5′′), 128.6 (C2′/C6′, C3′/C5′), 128.1 (C2′′/C6′′), 126.9 (C6), 123.9 (C5), 121.7 (C4a), 121.0 (H_A_C=).

HPLC-ESI^+^-QTOF-MS: *m*/*z* [M + H]^+^ calcd for C_22_H_16_N_2_: 309.1386; found: 309.1386.

Anal. calcd for C_22_H_16_N_2_: C, 85.69; H, 5.23; N, 9.08. Found: C, 85.66; H, 5.20; N, 9.12.

##### 4.1.1.2 (*E*)-4-Styryl-2-(*m*-tolyl)quinazoline (3b)

Yellow solid; yield: 0.271 g (84%); m.p. 122–123 °C; *R*_f_ = 0.50 (12.5% ethyl acetate–*n*-heptane).

IR (ATR): *ν̄*_max_ (cm^−1^) 3064 (C_sp^2^_–H), 1629 (C=N), 1530 (C=C_arom_), 968 (=C–H_*trans*_), 704 and 690 (C_arom_–H, benzene *m*-subst.).

NMR ^1^H (400 MHz, CDCl_3_): *δ* = 8.54–8.52 (m, 2H, H2′, H6′), 8.45 (d, *J* = 15.5 Hz, 1H, =CH_B_), 8.30 (dd, *J* = 8.4, 1.4 Hz, 1H, H5), 8.10 (dd, *J* = 8.4, 1.3 Hz, 1H, H8), 7.97 (d, *J* = 15.5 Hz, 1H, H_A_C=), 7.87 (ddd, *J* = 8.4, 6.9, 1.4 Hz, 1H, H7), 7.79–7.76 (m, 2H, H2′′/H6′′), 7.60 (ddd, *J* = 8.4, 6.9, 1.3 Hz, 1H, H6), 7.46 (dd, *J* = 8.4, 7.4 Hz, 1H, H5′), 7.43–7.39 (m, 3H, H3′′/H5′′, H4′′), 7.34 (dt, *J* = 7.4, 1.9 Hz, 1H, H4′), 2.53 (s, 3H, 3′-CH_3_).

NMR ^13^C (100 MHz, CDCl_3_): *δ* = 161.9 (C4), 160.4 (C2), 152.1 (C8a), 139.5 (=CH_B_), 138.5 (C1′), 138.2 (C3′), 136.2 (C1′′), 133.5 (C7), 131.3 (C4′), 129.6 (C8), 129.3 (C4′′), 129.1 (C2′), 128.9 (C3′′/C5′′), 128.5 (C5′), 128.1 (C2′′/C6′′), 126.8 (C6), 125.9 (C6′), 123.9 (C5), 121.7 (C4a), 121.1 (H_A_C=), 21.6 (3′-CH_3_).

HPLC-ESI^+^-QTOF-MS: *m*/*z* [M + H]^+^ calcd for C_23_H_18_N_2_: 323.1543, found: 323.1543.

Anal. calcd for C_23_H_18_N_2_: C, 85.68; H, 5.63; N, 8.69. Found: C, 85.65; H, 5.68; N, 8.67.

##### 4.1.1.3 (*E*)-2-(3-Nitrophenyl)-4-styrylquinazoline (3c)

White solid; yield: 0.295 g (83%); m.p. 178–179 °C; *R*_f_ = 0.40 (12.5% ethyl acetate–*n*-heptane).

IR (ATR): *ν̄*_max_ (cm^−1^) 3070 (C_sp^2^_–H), 1631 (C=N), 1560 (C=C_arom_), 1523 and 1346 (NO_2_), 969 (=C–H_*trans*_), 708 and 685 (C_arom_–H, benzene *p*-subst.).

NMR ^1^H (400 MHz, CDCl_3_): *δ* = 9.54 (t, *J* = 1.9 Hz, 1H, H2′), 9.05 (dt, *J* = 7.8, 1.4 Hz, 1H, H6′), 8.44 (d, *J* = 15.5 Hz, 1H, =CH_B_), 8.35 (ddd, *J* = 8.2, 2.4, 1.9 Hz, 1H, H4′), 8.31 (dd, *J* = 8.4, 1.4 Hz, 1H, H5), 8.11–8.09 (m, 1H, H8), 7.95 (d, *J* = 15.5 Hz, 1H, H_A_C=), 7.91 (ddd, *J* = 8.5, 7.0, 1.5 Hz, 1H, H7), 7.79–7.76 (m, 2H, H2′′/H6′′), 7.71 (t, *J* = 8.0 Hz, 1H, H5′), 7.66 (ddd, *J* = 8.4, 7.0, 1.4 Hz, 1H, H6), 7.50–7.46 (m, 2H, H3′′/H5′′), 7.45–7.41 (m, 1H, H4′′).

NMR ^13^C (100 MHz, CDCl_3_): *δ* = 162.3 (C4), 157.8 (C2), 151.8 (C8a), 148.8 (C3′), 140.4 (C1′), 140.1 (=CH_B_), 135.9 (C1′′), 134.4 (C6′), 133.9 (C7), 129.9 (C4′′), 129.4 (C8, C5′), 129.0 (C3′′/C5′′), 128.2 (C2′′/C6′′), 127.6 (C6), 124.8 (C4′), 123.9 (C5), 123.6 (C2′), 121.9 (C4a), 120.5 (H_A_C=). HPLC-ESI^+^-QTOF-MS: *m*/*z* [M + H]^+^ calcd for C_22_H_15_N_3_O_2_: 354.1237, found: 354.1235.

Anal. calcd for C_22_H_15_N_3_O_2_: C, 74.78; H, 4.28; N, 11.89. Found: C, 74.77; H, 4.30; N, 11.87.

##### 4.1.1.4 (*E*)-4-Styryl-2-(4-(trifluoromethyl)phenyl)quinazoline (3d)

White solid; yield: 0.316 g (84%); m.p. 141–142 °C; *R*_f_ = 0.50 (12.5% ethyl acetate–*n*-heptane).

IR (ATR): *ν̄*_max_ (cm^−1^) 3024 (C_sp^2^_–H), 1632 (C=N), 1534 (C=C_arom_), 1064 (C_sp^3^_–F), 961 (=C–H_*trans*_), 858 (C_arom_–H, benzene *p*-subst.).

NMR ^1^H (400 MHz, CDCl_3_): *δ* = 8.84–8.81 (m, 2H, H2′/H6′), 8.44 (d, *J* = 15.5 Hz, 1H, =CH_B_), 8.32 (ddd, *J* = 8.3, 1.6, 0.6, 1H, H5), 8.10 (ddd, *J* = 8.5, 1.3, 0.6 Hz, 1H, H8), 7.96 (d, *J* = 15.5 Hz, 1H, H_A_C=), 7.90 (ddd, *J* = 8.4, 6.9, 1.3 Hz, 1H, H7), 7.82–7.79 (m, 2H, H3′/H5′), 7.79–7.76 (m, 2H, H2′′/H6′′), 7.64 (ddd, *J* = 8.3, 6.9, 1.3 Hz, 1H, H6), 7.50–7.46 (m, 2H, H3′′/H5′′), 7.45–7.41 (m, 1H, H4′′).

NMR ^13^C (100 MHz, CDCl_3_): *δ* = 162.1 (C4), 158.7 (C2), 151.9 (C8a), 141.9 (C1′), 139.8 (=CH_B_), 136.0 (C1′′), 133.7 (C7), 131.9 (d, *J* = 32.3 Hz, C4′), 129.8 (C4′′), 129.4 (C8), 129.0 (C3′′/C5′′), 128.9 (C2′/C6′), 128.1 (C2′′/C6′′), 127.4 (C6), 125.4 (q, *J* = 3.7 Hz, C3′/C5′, 4′′-CF_3_), 123.9 (C5), 121.8 (C4a), 120.7 (H_A_C=).

HPLC-ESI^+^-QTOF-MS: *m*/*z* [M + H]^+^ calcd for C_23_H_15_F_3_N_2_: 377.1260, found: 377.1257.

Crystals suitable for X-ray single-crystal diffraction were obtained from a hexane/ethyl acetate (8 : 1) solution, and the crystal data for 3d were deposited at CCDC with deposition number 2349436:† chemical formula C_23_H_15_F_3_N_2_, Mr 376.37; orthorhombic, *P*2_1_2_1_2_1_; 100 K, cell dimensions *a*, *b*, *c* (Å) 5.0188 (6), 13.9106 (15), 25.986 (3) *α*, *β*, *γ* (°) 90, 90, 90. *V* (Å^3^) 1814.2 (4), *Z* = 4, *F*(000) = 776, Dx (Mg m^−3^) = 1.38, Mo Kα, *μ* (mm^−1^) = 0.10, crystal size (mm) = 0.32 × 0.05 × 0.04. Multi-scan absorption correction (SADABS2016/2), *T*_min_, *T*_max_ 0.418, 0.746. No. of measured, independent and observed [*I* > 2*σ*(*I*)] reflections 15 905, 4131, 3355, *R*_int_ = 0.096, (sin *θ*/λ)_max_ (Å^−1^) = 0.650, *θ* values (°): *θ*_max_ = 27.5, *θ*_min_ = 2.1; Range *h* = −16→16, *k* = −18→18, *l* = −32→33, Refinement, R[*F*^2^ > 2σ(F^2^)] = 0.087, wR(*F*^2^) = 0.195, *S* = 1.19. No. of reflections 4131, No. of parameters 253, No. of restraints 0. Weighting scheme: *w* = 1/*σ*^2^(*F*_o_^2^) + (0.0603*P*)^2^ + 2.0297*P* where *P* = (*F*_o_^2^ + 2*F*_c_^2^)/3. (*Δ*/*σ*) < 0.001, Δ*ρ*_max_, Δ*ρ*_min_ (e Å^−3^) 0.41, −0.43.

##### 4.1.1.5 (*E*)-4-Styryl-2-(3,4,5-trimethoxyphenyl)quinazoline (3e)

Yellow solid; yield: 0.275 g (69%); m.p. 152–153 °C; *R*_f_ = 0.26 (25% ethyl acetate–*n*-heptane).

IR (ATR): *ν̄*_max_ (cm^−1^) 2933 (C_sp^2^_–H), 1630 (C=N), 1441 (C=C_arom_), 961 (=C–H_*trans*_).

NMR ^1^H (400 MHz, CDCl_3_): *δ* = 8.39 (d, *J* = 15.4 Hz, 1H, =CH_B_), 8.30 (ddd, *J* = 8.4, 1.3, 0.6 Hz, 1H, H5), 8.09 (ddd, *J* = 8.4, 1.2, 0.6 Hz, 1H, H8), 8.02 (s, 2H, H2′/H6′), 7.96 (d, *J* = 15.4 Hz, 1H, H_A_C=), 7.88 (ddd, *J* = 8.4, 6.9, 1.3 Hz, 1H, H7), 7.76–7.73 (m, 2H, H2′′/H6′′), 7.60 (ddd, *J* = 8.4, 6.9, 1.2 Hz, 1H, H6), 7.50–7.45 (m, 2H, H3′′/H5′′), 7.44–7.40 (m, 1H, H4′′), 4.07 (s, 6H, 3′-/5′-OCH_3_), 3.96 (s, 3H, 4′-OCH_3_).

NMR ^13^C (100 MHz, CDCl_3_): *δ* = 161.9 (C4), 159.7 (C2), 153.4 (C3′/C5′), 152.0 (C8a), 140.5 (C4′), 139.5 (=CH_B_), 136.1 (C1′′), 134.0 (C1′), 133.5 (C7), 129.6 (C4′′), 129.3 (C8), 129.0 (C3′′/C5′′), 128.0 (C2′′/C6′′), 126.8 (C6), 123.9 (C5), 121.6 (C4a), 121.1 (H_A_C=), 106.0 (C2′/C6′), 61.0 (4′-OCH_3_), 56.3 (3′-/5′-OCH_3_).

UHPLC-ESI-Orbitrap-MS: *m*/*z* [M + H]^+^ calcd for C_25_H_22_N_2_O_3_: 399.17087, found: 399.17010.

Anal. calcd for C_25_H_22_N_2_O_3_: C, 75.36; H, 5.57; N, 7.03. Found: C, 75.40; H, 5.58; N, 7.01.

##### 4.1.1.6 (*E*)-4-Styryl-2-(5-methylfuran-2-yl)quinazoline (3f)

Yellow solid; yield: 0.241 g (77%); m.p. 171–172 °C; *R*_f_ = 0.33 (25% ethyl acetate–*n*-heptane).

IR (ATR): *ν̄*_max_ (cm^−1^) 2912 (C_sp^2^_–H), 1631 (C=N), 1547 (C=C_arom_), 1198 (C*–*O), 966 (=C–H_*trans*_). NMR ^1^H (400 MHz, CDCl_3_): *δ* = 8.33 (d, *J* = 15.5 Hz, 1H, =CH_B_), 8.25 (dd, *J* = 8.3, 1.3 Hz, 1H, H5), 8.09 (dd, *J* = 8.4, 1.3 Hz, 1H, H8), 7.91 (d, *J* = 15.5 Hz, 1H, H_A_C=), 7.84 (ddd, *J* = 8.5, 6.9, 1.5 Hz, 1H, H7), 7.75–7.73 (m, 2H, H2′′/H6′′), 7.56 (ddd, *J* = 8.3, 6.9, 1.3 Hz, 1H, H6), 7.48 (d, *J* = 3.1 Hz, 1H, H3′), 7.45–7.44 (m, 2H, H3′′/H5′′), 7.42–7.38 (m, 1H, H4′′), 6.24 (dd, *J* = 3.1, 1.0 Hz, 1H, H4′), 2.52 (s, 3H, 5′–CH_3_).

NMR ^13^C (100 MHz, CDCl_3_): *δ* = 162.3 (C4), 155.9 (C5′), 153.7 (C2), 151.7 (C8a), 151.5 (C2′), 139.8 (=CH_B_), 136.0 (C1′′), 133.7 (C7), 129.7 (C4′′), 129.1 (C8), 128.9 (C3′′/C5′′), 128.1 (C2′′/C6′′), 126.5 (C6), 124.0 (C5), 121.4 (C4a), 120.6 (H_A_C=), 115.5 (C3′), 108.8 (C4′), 14.3 (5′-CH_3_). UHPLC-ESI-Orbitrap-MS *m*/*z* [M + H]^+^ calcd for C_21_H_16_N_2_O: 313.13409; found: 313.13345.

Anal. calcd for C_21_H_16_N_2_O: C, 80.75; H, 5.16; N, 8.97. Found: C, 80.78; H, 5.13; N, 8.99.

##### 4.1.1.7 (*E*)-4-Styryl-2-(furan-3-yl)quinazoline (3g)

Yellow solid; yield: 0.206 g (69%); m.p. 175–176 °C; *R*_f_ = 0.35 (25% ethyl acetate–*n*-heptane).

IR (ATR): *ν̄*_max_ (cm^−1^) 3063 (C_sp^2^_–H), 1628 (C=N), 1535 (C=C_arom_), 1150 (C*–*O), 966 (=C–H_*trans*_). NMR ^1^H (400 MHz, CDCl_3_): *δ* = 8.46 (dd, *J* = 1.7, 0.8 Hz, 1H, H5′), 8.33 (d, *J* = 15.5 Hz, 1H, =CH_B_), 8.26 (dd, *J* = 8.3, 1.5 Hz, 1H, H5), 8.01 (dd, *J* = 8.4, 1.3 Hz, 1H, H8), 7.92 (d, *J* = 15.5 Hz, 1H, H_A_C=), 7.85 (ddd, *J* = 8.4, 6.9, 1.5 Hz, 1H, H7), 7.76–7.73 (m, 2H, H2′′/H6′′), 7.57 (ddd, *J* = 8.3, 6.9, 1.3 Hz, 1H, H6), 7.56 (t, *J* = 1.7 Hz, 1H, H2′), 7.48–7.44 (m, 2H, H3′′/H5′′), 7.43–7.39 (m, 1H, H4′′), 7.28 (dd, *J* = 1.7, 0.8 Hz, 1H, H4′).

NMR ^13^C (100 MHz, CDCl_3_): *δ* = 162.0 (C4), 157.0 (C2), 151.9 (C8a), 145.1 (C5′), 143.8 (C2′), 139.5 (=CH_B_), 136.1 (C1′′), 133.5 (C7), 129.7 (C4′′), 129.0 (C3′′/C5′′), 128.9 (C8), 128.1 (C2′′/C6′′), 127.9 (C3′), 126.6 (C6), 123.9 (C5), 121.6 (C4a), 120.7 (H_A_C=), 109.9 (C4′).

HPLC-ESI^+^-QTOF-MS: *m*/*z* [M + H]^+^ calcd for C_20_H_14_N_2_O: 299.1179; found: 299.1180.

Anal. calcd for C_20_H_14_N_2_O: C, 80.52; H, 4.73; N, 9.39; O, 5.36. Found: C, 80.56; H, 4.70; N, 9.36.

##### 4.1.1.8 (*E*)-4-(4-Bromostyryl)-2-phenylquinazoline (3h)

Yellow solid; yield: 0.298 g (77%); m.p. 123–125 °C; *R*_f_ = 0.50 (9% ethyl acetate–*n*-heptane).

IR (ATR): *ν̄*_max_ (cm^−1^) 3022 (C_sp^2^_–H), 1629.26 (C=N), 1558 (C=C_arom_), 967 (=C–H_*trans*_), 816 (C_arom_–H, benzene *p*-subst.), 659 (C_sp^2^_–Br).

NMR ^1^H (400 MHz, CDCl_3_): *δ* = 8.73–8.69 (m, 2H, H2′/H6′), 8.38 (d, *J* = 15.5 Hz, 1H, =CH_B_), 8.27 (dd, *J* = 8.4, 1.4 Hz, 1H, H5), 8.10 (dd, *J* = 8.4, 1.3 Hz, 1H, H8), 7.95 (d, *J* = 15.5 Hz, 1H, H_A_C=), 7.88 (ddd, *J* = 8.4, 6.8, 1.2 Hz, 1H, H7), 7.64–7.52 (m, 8H, H6, H3′/H5′, H4′, H2′′/H6′′, H3′′/H5′′).

NMR ^13^C (100 MHz, CDCl_3_): *δ* = 161.5 (C4), 152.1 (C8a), 138.2 (C1′), 138.3 (=CH_B_), 134.6 (C1′′), 133.5 (C7), 132.0 (C3′′/C5′′), 130.5 (C4′), 129.5 (C2′′/C6′′), 129.3 (C8), 128.6 (C2′/C6′, C3′/C5′), 126.7 (C6), 124.0 (C4′′), 123.6 (C5), 121.5 (C4a), 121.0 (H_A_C=).

UHPLC-ESI^+^-Orbitrap-MS: *m*/*z* [M + H]^+^ calcd for C_22_H_15_BrN_2_, [^79^Br]: 387.04926; found [^79^Br]: 387.04919.

##### 4.1.1.9 (*E*)-4-(4-Chlorostyryl)-2-phenylquinazoline (3i)

Yellow solid; yield: 0.264 g (77%); m.p. 115–116 °C; *R*_f_ = 0.50 (9% ethyl acetate–*n*-heptane).

IR (ATR): *ν̄*_max_ (cm^−1^) 3059 (C_sp^2^_–H), 1629 (C=N), 1559 (C=C_arom_), 968 (=C–H_*trans*_), 817 (C_arom_–H, benzene *p*-subst.), 686 (C_sp^2^_*–*Cl).

NMR ^1^H (400 MHz, CDCl_3_): *δ* = 8.72–8.69 (m, 2H, H2′/H6′), 8.39 (d, *J* = 15.4 Hz, 1H, =CH_B_), 8.27 (dd, *J* = 8.4, 1.3 Hz, 1H, H5), 8.10 (dd, *J* = 8.4, 1.3 Hz, 1H, H8), 7.93 (d, *J* = 15.4 Hz, 1H, H_A_C=), 7.88 (ddd, *J* = 8.4, 6.9, 1.3 Hz, 1H, H7), 7.71–7.68 (m, 2H, H2′′/H6′′), 7.61 (ddd, *J* = 8.3, 6.9, 1.3 Hz, 1H, H6), 7.58–7.54 (m, 2H, H3′/H5′), 7.54–7.50 (m, 1H, H4′), 7.45–7.42 (m, 2H, H3′′/H5′′).

NMR ^13^C (100 MHz, CDCl_3_): *δ* = 161.6 (C4), 160.2 (C2), 152.1 (C8a), 138.5 (C1′), 138.0 (=CH_B_), 135.4 (C4′′), 134.6 (C1′′), 133.6 (C7), 130.5 (C4′), 129.4 (C8), 129.2 (C2′′/C6′′, C3′′/C5′′), 128.6 (C2′/C6′, C3′/C5′), 126.9 (C6), 123.7 (C5), 121.6 (C4a), 121.5 (H_A_C=).

HPLC-ESI^+^-QTOF-MS: *m*/*z* [M + H]^+^ calcd for C_22_H_15_ClN_2_, [^35^Cl/^37^Cl]: 343.0997/345.0975, found [^35^Cl/^37^Cl]: 343.0992/345.0979.

##### 4.1.1.10 (*E*)-4-(4-Trifluoromethylstyryl)-2-phenylquinazoline (3j)

Yellow solid; yield: 0.316 g (84%); m.p. 145–146 °C; *R*_f_ = 0.47 (9% ethyl acetate–*n*-heptane).

FTIR (ATR) *ν̄*_max_ (cm^−1^): 3063 (C_sp^2^_–H), 1637 (C=N), 1562 (C=C_arom_), 1319 (C_sp^3^_–F), 969 (=C–H_*trans*_), 831 (C_arom_–H, benzene *p*-subst.).

NMR ^1^H (400 MHz, CDCl_3_): *δ* = 8.73–8.70 (m, 2H, H2′/H6′), 8.45 (d, *J* = 15.5 Hz, 1H, =CH_B_), 8.29 (dd, *J* = 8.4, 1.4 Hz, 1H, H5), 8.11 (dd, *J* = 8.4, 1.3 Hz, 1H, H8), 8.03 (d, *J* = 15.5 Hz, 1H, H_A_C=), 7.89 (ddd, *J* = 8.4, 6.9, 1.4 Hz, 1H, H7), 7.86 (d, *J* = 8.0 Hz, 2H, H2′′/H6′′), 7.72 (d, *J* = 8.0 Hz, 2H, H3′′/H5′′), 7.63 (ddd, *J* = 8.3, 6.9, 1.3 Hz, 1H, H6), 7.59–7.53 (m, 3H, H3′/H5′, H4′).

NMR ^13^C (100 MHz, CDCl_3_): *δ* = 161.2 (C4), 160.2 (C2), 152.2 (C8a), 139.5 (C1′′), 138.3 (C1′), 137.6 (=CH_B_), 133.7 (C7), 130.6 (C4′), 129.5 (C8), 129.3 (C4′′), 128.6 (C3′/C5′, C2′/C6′), 128.1 (C2′′/C6′′), 127.1 (C6), 125.9 (q, *J* = 3.7 Hz, C3′′/C5′′, 4′′–CF_3_), 123.7 (C5), 123.4 (H_A_C=), 121.7 (C4a).

UHPLC-ESI^+^-Orbitrap-MS: *m*/*z* [M + H]^+^ calcd for C_23_H_15_F_3_N_2_: 377.12582, found: 377.12656.

##### 4.1.1.11 (*E*)-4-(2,3-Dichlorostyryl)-2-phenylquinazoline (3k)

Yellow solid; yield: 0.283 g (75%); m.p. 163–165 °C; *R*_f_ = 0.34 (9% ethyl acetate–*n*-heptane).

IR (ATR): *ν̄*_max_ (cm^−1^) 3065 (C_sp^2^_–H), 1625 (C=N), 1556 (C=C_arom_), 967 (=C–H_*trans*_), 689 (C_sp^2^_–Cl).

NMR ^1^H (400 MHz, CDCl_3_): *δ* = 8.80 (d, *J* = 15.5 Hz, 1H, =CH_B_), 8.74–8.71 (m, 2H, H2′/H6′), 8.26 (dd, *J* = 8.3, 1.3 Hz, 1H, H5), 8.11 (dd, *J* = 8.2, 1.3 Hz, 1H, H8), 7.91 (d, *J* = 15.5 Hz, 1H, H_A_C=), 7.91–7.86 (m, 1H, H7), 7.77 (dd, *J* = 7.9, 1.5 Hz, 1H, H4′′), 7.61 (ddd, *J* = 8.3, 6.9, 1.3 Hz, 1H, H6), 7.59–7.52 (m, 3H, H3′/H5′, H4′), 7.51 (dd, *J* = 7.9, 1.5 Hz, 1H, H6′′), 7.30 (td, *J* = 7.9, 1.5 Hz, 1H, H5′′).

NMR ^13^C (100 MHz, CDCl_3_): *δ* = 161.3 (C4), 160.2 (C2), 152.2 (C8a), 138.3 (C1′), 136.9 (C1′′), 135.5 (=CH_B_), 134.0 (C3′′), 133.7 (C7), 133.0 (C2′′), 130.8 (C6′′), 130.6 (C4′), 129.5 (C8), 128.6 (C2′/C6′, C3′/C5′), 127.4 (C5′′), 127.1 (C6), 125.8 (C4′′), 125.0 (H_A_C=), 123.7 (C5), 121.7 (C4a). UHPLC-ESI^+^-Orbitrap-HRMS: *m*/*z* [M + H]^+^ calcd for C_22_H_14_Cl_2_N_2_, [^35^Cl, ^35^Cl]: 377.06068; found: [^35^Cl, ^35^Cl]: 377.06104.

##### 4.1.1.12 (*E*)-4-(2,4-Dichlorostyryl)-2-phenylquinazoline (3l)

Yellow solid; yield: 0.287 g (76%); m.p. 177–178 °C; *R*_f_ = 0.44 (9% ethyl acetate–*n*-heptane).

IR (ATR): *ν̄*_max_ (cm^−1^) 3025 (C_sp^2^_–H), 1626 (C=N), 1559 (C=C_arom_), 968 (=C–H_*trans*_), 692 (C_sp^2^_–Cl). NMR ^1^H (400 MHz, CDCl_3_): *δ* = 8.74 (dd, *J* = 15.5, 0.6 Hz, 1H, =CH_B_), 8.73–8.71 (m, 2H, H2′/H6′), 8.25 (dd, *J* = 8.3, 1.4 Hz, 1H, H5), 8.11 (dd, *J* = 8.5, 1.3 Hz, 1H, H8), 7.92 (d, *J* = 15.5 Hz, 1H, H_A_C=), 7.88 (ddd, *J* = 8.5, 6.9, 1.4 Hz, 1H, H7), 7.80 (d, *J* = 8.5 Hz, 1H, H6′′), 7.61 (ddd, *J* = 8.3, 6.9, 1.3 Hz, 1H, H6), 7.58–7.52 (m, 3H, H3′/H5′, H4′), 7.51 (d, *J* = 2.1 Hz, 1H, H3′′), 7.34 (ddd, *J* = 8.5, 2.1, 0.6 Hz, 1H, H5′′).

NMR ^13^C (100 MHz, CDCl_3_): *δ* = 161.3 (C4), 160.2 (C2), 152.2 (C8a), 138.3 (C1′), 135.5 (C2′′, C4′′), 134.3 (=CH_B_), 133.6 (C7), 133.1 (C1′′), 130.5 (C4′), 130.1 (C3′′), 129.5 (C8), 128.6 (C2′/C6′, C3′/C5′), 128.4 (C6′′), 127.5 (C5′′), 127.1 (C6), 124.1 (H_A_C=), 123.7 (C5), 121.7 (C4a).

UHPLC-ESI^+^-Orbitrap-HRMS: *m*/*z* [M + H]^+^ calcd for C_22_H_14_Cl_2_N_2_ [^35^Cl, ^35^Cl]: 377.06068; found: [^35^Cl, ^35^Cl]: 377.06061.

##### 4.1.1.13 (*E*)-4-(3,4-Dichlorostyryl)-2-phenylquinazoline (3m)

Yellow solid; yield: 0.290 g (77%); m.p. 174–175 °C; *R*_f_ = 0.47 (9% ethyl acetate–*n*-heptane).

IR (ATR): *ν̄*_max_ (cm^−1^) 3065 (C_sp^2^_–H), 1629 (C=N), 1557 (C=C_arom_), 967 (=C–H_*trans*_), 689 (C_sp^2^_–Cl). NMR ^1^H (400 MHz, CDCl_3_): *δ* = 8.71–8.68 (m, 2H, H2′/H6′), 8.32 (d, *J* = 15.4 Hz, 1H, =CH_B_), 8.26 (dd, *J* = 8.5, 1.4 Hz, 1H, H5), 8.10 (dd, *J* = 8.5, 1.3 Hz, 1H, H8), 7.92 (d, *J* = 15.4 Hz, 1H, H_A_C=), 7.88 (ddd, *J* = 8.4, 6.9, 1.4 Hz, 1H, H7), 7.84 (d, *J* = 2.0 Hz, 1H, H2′′), 7.61 (ddd, *J* = 8.4, 6.9, 1.3 Hz, 1H, H6), 7.58–7.51 (m, 5H, H3′/H5′, H4′, H5′′, H6′′).

NMR ^13^C (100 MHz, CDCl_3_): *δ* = 161.1 (C4), 160.1 (C2), 152.1 (C8a), 138.3 (C1′), 136.6 (=CH_B_), 136.2 (C1′′), 133.7 (C7), 133.4 (C3′′), 133.2 (C4′′), 130.9 (C5′′), 130.6 (C4′), 129.5 (C8, C2′′), 128.6 (C2′/C6′, C3′/C5′), 127.1 (C6, C6′′), 123.7 (C5), 122.7 (H_A_C=), 121.6 (C4a).

HPLC-ESI^+^-QTOF-MS: *m*/*z* [M + H]^+^, calcd for C_22_H_14_Cl_2_N_2_ [^35^Cl, ^35^Cl]: 377.0607, found: 377.0605; calcd [^35^Cl, ^37^Cl]: 379.0581, found: 379.0583; calcd [^37^Cl, ^37^Cl]: 380.0607, found: 380.0610.

##### 4.1.1.14 (*E*)-4-(2,6-Dichlorostyryl)-2-phenylquinazoline (3n)

Yellow solid; yield: 0.332 g (88%); m.p. 137–139 °C; *R*_f_ = 0.53 (9% ethyl acetate–*n*-heptane).

IR (ATR): *ν̄*_max_ (cm^−1^) 3058 (C_sp^2^_–H), 1626 (C=N), 1558 (C=C_arom_), 975 (=C–H_*trans*_), 687 (C_sp^2^_–Cl). NMR ^1^H (400 MHz, CDCl_3_): *δ* = 8.75–8.72 (m, 2H, H2′/H6′), 8.69 (d, *J* = 15.8 Hz, 1H, =CH_B_), 8.29 (d, *J* = 15.8 Hz, 1H, H_A_C=), 8.25 (dd, *J* = 8.3, 1.3 Hz, 1H, H5), 8.11| (dd, *J* = 8.4, 1.3 Hz, 1H, H8), 7.88 (ddd, *J* = 8.4, 6.9, 1.3 Hz, 1H, H7), 7.61 (ddd, *J* = 8.3, 6.9, 1.3 Hz, 1H, H6), 7.59–7.50 (m, 3H, H3′/H5′, H4′), 7.33 (ddd, *J* = 8.1, 1.4, 0.7 Hz, 1H, H3′′), 7.27 (td, *J* = 8.5, 5.6 Hz, 1H, H4′′), 7.15–7.10 (m, 1H, H5′′).

NMR ^13^C (100 MHz, CDCl_3_): *δ* = 161.4 (C4), 160.2 (C2), 152.2 (C8a), 138.4 (C1′), 135.2 (C2′′/C6′′), 133.6 (C7, C1′′), 133.0 (=CH_B_), 130.5 (C4′), 129.5 (C8), 129.4 (H_A_C=), 129.3 (C4′′), 128.9 (C3′′/C5′′), 128.6 (C2′/C6′, C3′/C5′), 127.1 (C6), 124.0 (C5), 121.9 (C4a).

UHPLC-ESI^+^-Orbitrap-HRMS: *m*/*z* [M + H]^+^ calcd for C_22_H_14_Cl_2_N_2_ [^35^Cl, ^35^Cl]: 377.06068; found: 377.06085.

##### 4.1.1.15 (*E*)-4-(2-Chloro-6-fluorostyryl)-2-phenylquinazoline (3o)

Yellow solid; yield: 0.318 (88%); m.p. > 141 °C (decomposition); *R*_f_ = 0.50 (9% ethyl acetate–*n*-heptane). IR (ATR): *ν̄*_max_ (cm^−1^) 3065 (C_sp^2^_–H), 1622 (C=N), 1557 (C=C_arom_), 1210 (C_sp^2^_–F), 974 (=C–H_*trans*_), 686 (C_sp^2^_–Cl).

NMR ^1^H (400 MHz, CDCl_3_): *δ* = 8.75–8.72 (m, 2H, H2′/H6′), 8.69 (d, *J* = 15.8 Hz, 1H, =CH_B_), 8.29 (d, *J* = 15.8 Hz, 1H, H_A_C=), 8.25 (da, *J* = 8.7 Hz, 1H, H5), 8.11 (dd, *J* = 8.4, 1.3 Hz, 1H, H8), 7.88 (ddd, *J* = 8.4, 6.9, 1.3 Hz, 1H, H7), 7.61 (ddd, *J* = 8.3, 6.9, 1.3 Hz, 1H, H6), 7.59–7.50 (m, 3H, H3′/H5′, H4′), 7.33 (dd, *J* = 8.1, 1.4 Hz, 1H, H3′′), 7.27 (td, *J* = 8.1, 5.6 Hz, 1H, H4′′), 7.15–7.10 (m, 1H, H5′′).

NMR ^13^C (100 MHz, CDCl_3_): *δ* = 162.1 (d, *J* = 255.2 Hz, C6′′), 161.8 (C4), 160.1 (C2), 152.2 (C8a), 138.4 (C1′), 136.1 (d, *J* = 5.7 Hz, C2′′), 133.6 (C7), 130.5 (C4′), 129.9 (d, *J* = 10.5 Hz, C4′′), 129.7 (d, *J* = 2.3 Hz, =CH_B_), 129.3 (C8), 128.6 (C2′/C6′, C3′/C5′), 128.0 (d, *J* = 14.5 Hz, H_A_C=), 127.1 (C6), 126.1 (d, *J* = 3.1 Hz, C3′′), 123.9 (C5), 123.2 (d, *J* = 14.0 Hz, C1′′), 121.8 (C4a), 114.9 (d, *J* = 23.5 Hz, C5′′).

UHPLC-ESI^+^-Orbitrap-MS: *m*/*z* [M + H]^+^ calcd for C_22_H_14_ClFN_2_ [^35^Cl]: 361.09015; found: 361.09078.

#### 4.1.2 General procedure for the synthesis of (*E*)-4-styrylquinazolines 5

In a 20 mL Radleys reaction tube, 2′-aminochalcones 1 (1.0 mmol), trimethyl orthoformate 4 (3.5 mmol), and ammonium acetate (3.0 mmol) were mixed in ethanol (2.0 mL) at room temperature and then capped. The obtained suspensions were stirred magnetically at 80 °C until completion of the reactions (TLC monitored). Then, the crude mixtures were allowed to cool down to room temperature, and the excess of trimethyl orthoformate and ethanol was removed under reduced pressure. In each case, the residue was extracted with ethyl acetate (3 × 30 mL) followed by washing with water (30 mL) and brine. The combined organic layers were dried over anhydrous sodium sulfate, and the solvent was removed under reduced pressure. The resulting crude was purified by flash chromatography on silica gel using *n*-heptane–ethyl acetate mixtures as the eluent (8 : 1 to 5 : 1) to obtain the target compounds 5 as solid substances.

##### 4.1.2.1 (*E*)-4-(Styryl)quinazoline (5a)

Yellow solid; yield: 0.197 g (85%); m.p. 94–96 °C; *R*_f_ = 0.22 (25% ethyl acetate–*n*-heptane).

IR (ATR): *ν̄*_max_ (cm^−1^) 3032 (C_sp^2^_–H), 1626 (C=N), 1557 (C=C_arom_), 974 (=C–H_*trans*_).

NMR ^1^H (400 MHz, CDCl_3_): *δ* = 9.29 (s, 1H, H2), 8.32 (dd, *J* = 8.4, 1.5 Hz, 1H, H5), 8.28 (d, *J* = 15.5 Hz, 1H, =CH_B_), 8.05 (dt, *J* = 8.5, 1.3 Hz, 1H, H8), 7.93 (d, *J* = 15.5 Hz, 1H, H_A_C=), 7.90 (ddd, *J* = 8.4, 6.9, 1.3 Hz, 1H, H7), 7.74–7.71 (m, 2H, H2′/H6′), 7.67 (ddd, *J* = 8.3, 6.9, 1.3 Hz, 1H, H6), 7.47–7.38 (m, 3H, H3′/H5′, H4′).

NMR ^13^C (100 MHz, CDCl_3_): *δ* = 162.1 (C4), 154.8 (C2), 151.2 (C8a), 139.9 (=CH_B_), 135.9 (C1′), 133.6 (C7), 129.8 (C4′), 129.1 (C8), 129.0 (C2′/C6′), 128.1 (C3′/C5′), 127.5 (C6), 123.9 (C5), 123.1 (C4a), 120.5 (H_A_C=).

HPLC-ESI^+^-QTOF-MS: *m*/*z* [M + H]^+^ calcd for C_16_H_12_N_2_: 233.1073; found: 233.1076.

Anal. calcd for C_16_H_12_N_2_: C, 82.73; H, 5.21; N, 12.06. Found: C, 82.77; H, 5.20; N, 12.03.

##### 4.1.2.2 (*E*)-4-(4-Methoxystyryl)quinazoline (5b)

Yellow solid; yield: 0.223 g (85%); m.p. 93–95 °C; *R*_f_ = 0.22 (25% ethyl acetate–*n*-heptane).

IR (ATR): *ν̄*_max_ (cm^−1^) 3033 (C_sp^2^_–H), 1622 (C=N), 1563 (C=C_arom_), 978 (=C–H_*trans*_).

NMR ^1^H (400 MHz, CDCl_3_): *δ* = 9.25 (s, 1H, H2), 8.30 (dd, *J* = 8.5, 1.6 Hz, 1H, H5), 8.25 (d, *J* = 15.4 Hz, 1H, =CH_B_), 8.02 (dd, *J* = 8.5, 1.0 Hz, 1H, H8), 7.88 (ddd, *J* = 8.3, 6.9, 1.3 Hz, 1H, H7), 7.79 (d, *J* = 15.4 Hz, 1H, H_A_C=), 7.70–7.66 (m, 2H, H2′/H6′), 7.64 (ddd, *J* = 8.4, 6.9, 1.4 Hz, 1H, H6), 6.98–6.95 (m, 2H, H3′/H5′), 3.86 (s, 3H, 4′–OCH_3_).

NMR ^13^C (100 MHz, CDCl_3_): *δ* = 162.4 (C4), 161.1 (C4′), 151.1 (C8a), 154.8 (C2), 139.6 (=CH_B_), 133.5 (C7), 129.7 (C2′/C6′), 129.0 (C8), 128.7 (C1′), 127.3 (C6), 123.9 (C5), 123.0 (C4a), 118.0 (H_A_C=), 114.4 (C3′/C5′), 55.4 (4′-OCH_3_).

HPLC-ESI^+^-QTOF-MS: *m*/*z* [M + H]^+^ calcd for C_17_H_14_N_2_O: 263.1179; found: 263.1185.

Anal. calcd for C_17_H_14_N_2_O: C, 77.84; H, 5.38; N, 10.68. Found: C, 77.84; H, 5.36; N, 10.65.

Crystals suitable for X-ray single-crystal diffraction were obtained from a hexane/ethyl acetate (8 : 1) solution, and the crystal data for 5b were deposited at CCDC with deposition number 2349524:† chemical formula C_17_H_14_N_2_O, Mr 262.30; orthorhombic, *Pca*2_1_; 100 K, cell dimensions *a*, *b*, *c* (Å) 24.6763(13), 3.9753(2), 13.3702(7), *α*, *β*, *γ* (°) 90, 90, 90. *V* (Å^3^) = 1311.56(12), *Z* = 4, *F*(000) = 552, Dx (Mg m^−3^) = 1.328, Mo Kα, *μ* (mm^−1^) = 0.084, crystal size (mm) = 0.10 × 0.06 × 0.04. Multi-scan absorption correction (SADABS2016/2), *T*_min_, *T*_max_ 0.6632, 0.7455. No. of measured, independent and observed [*I* > 2*σ*(*I*)] reflections 24 495, 2885, 2668, *R*_int_ = 0.051, (sin *θ*/*λ*)_max_ (Å^−1^) = 0.642, *θ* values (°): *θ*_max_ = 27.1, *θ*_min_ = 2.2; range *h* = −31 → 31, *k* = −5 → 5, *l* = −17 → 17, refinement, *R*[*F*^2^ > 2*σ*(*F*^2^)] = 0.031, wR(*F*^2^) = 0.074, *S* = 1.05. No. of reflections 2885, no. of parameters 182, no. of restraints 1. Weighting scheme: *w* = 1/*σ*^2^(*F*_o_^2^) + (0.0603*P*)^2^ + 0.3624*P* where *P* = (*F*_o_^2^ + 2*F*_c_^2^)/3. (Δ/*σ*) < 0.001, Δ*ρ*_max_, Δ*ρ*_min_ (e Å^−3^) 0.14, −0.18.

##### 4.1.2.3 (*E*)-4-(4-Bromostyryl)quinazoline (5c)

Yellow solid; yield: 0.240 g (77%); m.p. 150–152 °C; *R*_f_ = 0.22 (25% ethyl acetate–*n*-heptane).

IR (ATR): *ν̄*_max_ (cm^−1^) 3038 (C_sp^2^_–H), 1627 (C=N), 1563 (C=C_arom_), 983 (=C–H_*trans*_), 661 (C_sp^2^_–Br). NMR ^1^H (400 MHz, CDCl_3_): *δ* = 9.29 (s, 1H, H2), 8.31 (dd, *J* = 8.5, 1.3 Hz, 1H, H5), 8.22 (d, *J* = 15.5 Hz, 1H, =CH_B_), 8.06 (dt, *J* = 8.4, 1.0 Hz, 1H, H8), 7.92 (d, *J* = 15.5 Hz, 1H, H_A_C=), 7.91 (ddd, *J* = 8.4, 6.9, 1.4 Hz, 1H, H7), 7.68 (ddd, *J* = 8.3, 6.9, 1.3 Hz, 1H, H6), 7.61–7.56 (m, 4H, H2′/H6′, H3′/H5′).

NMR ^13^C (100 MHz, CDCl_3_): *δ* = 161.7 (C4), 154.8 (C2), 151.2 (C8a), 138.5 (=CH_B_), 134.8 (C1′), 133.7 (C7), 132.2 (C3′/C5′), 129.4 (C2′/C6′), 129.2 (C8), 127.7 (C6), 124.0 (C4′), 123.8 (C5), 123.1 (C4a), 121.1 (H_A_C=).

HPLC-ESI^+^-QTOF-MS: *m*/*z* [M + H]^+^ calcd for C_16_H_11_BrN_2_ [^79^Br]: 311.0178; found: 311.0177.

##### 4.1.2.4 (*E*)-4-(4-Chlorostyryl)quinazoline (5d)

Yellow solid; yield: 0.221 g (83%); m.p. 162–163 °C; *R*_f_ = 0.22 (25% ethyl acetate–*n*-heptane).

IR (ATR): *ν̄*_max_ (cm^−1^) 3058 (C_sp^2^_–H), 1626 (C=N), 1535 (C=C_arom_), 977 (=C–H_*trans*_), 682 (C_sp^2^_–Cl). NMR ^1^H (400 MHz, CDCl_3_): *δ* = 9.29 (s, 1H, H2), 8.30 (dd, *J* = 8.4, 1.7 Hz, 1H, H5), 8.23 (d, *J* = 15.5 Hz, 1H, =CH_B_), 8.05 (dd, *J* = 8.5, 1.8 Hz, 1H, H8), 7.91 (ddd, *J* = 8.4, 6.9, 1.7 Hz, 1H, H7), 7.89 (d, *J* = 15.5 Hz, 1H, H_A_C=), 7.67 (ddd, *J* = 8.4, 6.9, 1.6 Hz, 1H, H6), 7.67–7.63 (m, 2H, H2′/H6′), 7.43–7.40 (m, 2H, H3′/H5′).

NMR ^13^C (100 MHz, CDCl_3_): *δ* = 161.8 (C4), 154.8 (C2), 151.2 (C8a), 138.4 (=CH_B_), 135.6 (C4′), 134.4 (C1′), 133.7 (C7), 129.2 (C2′/C6′, C3′/C5′), 129.1 (C8), 127.6 (C6), 123.8 (C5), 123.1 (C4a), 121.0 (H_A_C=).

HPLC-ESI^+^-QTOF-MS: *m*/*z* [M + H]^+^ calcd for C_16_H_11_ClN_2_ [^35^Cl]: 267.0684; found [^35^Cl]: 267.0685.

##### 4.1.2.5 (*E*)-4-(4-Fluorostyryl)quinazoline (5e)

Yellow solid; yield: 0.198 g (79%); m.p. 134–136 °C; *R*_f_ = 0.22 (25% ethyl acetate-*n*-heptane).

IR (ATR): *ν̄*_max_ (cm^−1^) 3031 (C_sp^2^_–H), 1628 (C=N), 1566 (C=C_arom_), 1157 (C_sp^2^_–F), 969 (=C–H_*trans*_). NMR ^1^H (400 MHz, CDCl_3_): *δ* = 9.28 (s, 1H, H2), 8.30 (dt, *J* = 8.8, 1.0 Hz, 1H, H5), 8.24 (d, *J* = 15.5 Hz, 1H, =CH_B_), 8.04 (dd, *J* = 8.5, 1.0 Hz, 1H, H8), 7.90 (ddd, *J* = 8.4, 6.9, 1.3 Hz, 1H, H7), 7.84 (d, *J* = 15.5 Hz, 1H, H_A_C=), 7.72–7.69 (m, 2H, H2′/H6′), 7.66 (ddd, *J* = 8.3, 6.9, 1.3 Hz, 1H, H6), 7.16–7.11 (m, 2H, H3′/H5′).

NMR ^13^C (100 MHz, CDCl_3_): *δ* = 163.6 (d, *J* = 251.3 Hz, C4′), 161.9 (C4), 154.8 (C2), 151.2 (C8a), 138.6 (=CH_B_), 133.7 (C7), 132.1 (d, *J* = 2.2 Hz, C1′), 129.8 (d, *J* = 8.2 Hz, C2′/C6′), 129.1 (C8), 127.6 (C6), 123.8 (C5), 123.0 (C4a), 120.2 (d, *J* = 2.3 Hz, H_A_C=), 116.1 (d, *J* = 22.0 Hz, C3′/C5′). HPLC-ESI^+^-QTOF-MS:*m*/*z* [M + H]^+^ calcd for C_16_H_11_FN_2_: 251.097; found: 251.0978.

##### 4.1.2.6 (*E*)-4-(4-(Trifluoromethyl)styryl)quinazoline (5f)

Yellow solid; yield: 0.243 g (81%); m.p. 156–157 °C; *R*_f_ = 0.26 (25% ethyl acetate–*n*-heptane).

IR (ATR): *ν̄*_max_ (cm^−1^) 3039 (C_sp^2^_–H), 1629 (C=N), 1541 (C=C_arom_), 1109 (C_sp^3^_–F), 973 (=C–H_*trans*_). NMR ^1^H (400 MHz, CDCl_3_): *δ* = 9.32 (s, 1H, H2), 8.29 (d, *J* = 8.6 Hz, 1H, H5), 8.29 (d, *J* = 15.5 Hz, 1H, =CH_B_), 8.08 (d, *J* = 8.52 Hz, 1H, H8), 8.00 (d, *J* = 15.5 Hz, 1H, H_A_C=), 7.93 (ddd, *J* = 8.4, 6.8, 1.4 Hz, 1H, H7), 7.82 (d, *J* = 8.1 Hz, 2H, H2′/H6′), 7.70 (ddd, *J* = 8.4, 6.9, 1.2 Hz, 1H, H6), 7.70 (d, *J* = 8.1 Hz, 2H, H3′/H5′).

NMR ^13^C (100 MHz, CDCl_3_): *δ* = 161.4 (C4), 154.8 (C2), 151.3 (C8a), 139.3 (C1′), 138.0 (=CH_B_), 133.8 (C7), 131.2 (d, *J* = 32.4 Hz, C4′), 129.2 (C8), 127.8 (C6), 128.1 (C2′/C6′, C3′/C5′), 125.9 (q, *J* = 3.8 Hz, 4′-CF_3_), 123.7 (C5), 123.1 (C4a), 122.9 (H_A_C=).

HPLC-ESI^+^-QTOF-MS: *m*/*z* [M + H]^+^ calcd for C_17_H_11_F_3_N_2_: 301.0947; found: 301.0946.

Crystals suitable for X-ray single-crystal diffraction were obtained from a hexane/ethyl acetate (8 : 1) solution, and the crystal data for 5f were deposited at CCDC with deposition number 2349522:† chemical formula C_17_H_11_F_3_N_2_, Mr 300.28; monoclinic, *P*2_1_/*c*; 100 K, cell dimensions *a*, *b*, *c* (Å) 13.1299 (8), 13.2052 (9), 7.7725 (4) *α*, *β*, *γ* (°) 90, 95.083 (2), 90. *V* (Å^3^) = 1342.32 (14), *Z* = 4, *F* (000) = 616, Dx (Mg m^−3^) = 1.49, Mo Kα, *μ* (mm^−1^) = 0.118, crystal size (mm) = 0.12 × 0.06 × 0.05. Multi-scan absorption correction (SADABS2016/2), *T*_min_, *T*_max_ 0.669, 0.746. No. of measured, independent and observed [*I* > 2*σ*(*I*)] reflections 30 816, 2951, 2229, *R*_int_ = 0.076, (sin *θ*/*λ*)_max_ (Å^−1^) = 0.642, *θ*values (°): *θ*_max_ = 27.1, *θ*_min_ = 2.2; range *h* = −16 → 16, *k* = −16 → 16, *l* = −9 → 9, refinement, *R*[*F*^2^ > 2*σ*(*F*^2^)] = 0.055, wR(*F*^2^) = 0.132, *S* = 1.8. No. of reflections 2951, no. of parameters 199, no. of restraints 0. Weighting scheme: *w* = 1/*σ*^2^(*F*_o_^2^) + (0.0381*P*)^2^ + 1.6974*P* where *P* = (*F*_o_^2^ + 2*F*_c_^2^)/3. (Δ/*σ*) < 0.001, Δ*ρ*_max_, Δ*ρ*_min_ (e Å^−3^) 0.52, −0.48.

##### 4.1.2.7 (*E*)-4-(2,6-dichlorostyryl)quinazoline (5g)

Yellow solid; yield: 0.247 g (82%); m.p. 154–156 °C; *R*_f_ = 0.30 (17% ethyl acetate–*n*-heptane).

IR (ATR): *ν̄*_max_ (cm^−1^) 3032 (C_sp^2^_–H), 1631 (C=N), 1538 (C=C_arom_), 968 (=C–H_*trans*_), 756 (C_sp^2^_–Cl). NMR ^1^H (400 MHz, CDCl_3_): *δ* = 9.36 (s, 1H, H2), 8.32 (d, *J* = 15.5 Hz, 1H, =CH_B_), 8.25 (dd, *J* = 8.6, 1.4 Hz, 1H, H5), 8.11 (d, *J* = 15.5 Hz, 1H, H_A_C=), 8.07 (dd, *J* = 8.5, 1.4 Hz, 1H, H8), 7.92 (ddd, *J* = 8.5, 6.9, 1.4 Hz, 1H, H7), 7.67 (ddd, *J* = 8.5, 6.9, 1.4 Hz, 1H, H6), 7.42 (d, *J* = 8.1 Hz, 2H, H3′/H5′), 7.22 (t, *J* = 8.1 Hz, 1H, H4′).

NMR ^13^C (100 MHz, CDCl_3_): *δ* = 161.6 (C4), 155.0 (C2), 151.2 (C8a), 135.2 (C2′/C6′), 133.8 (C7), 133.5 (C1′), 133.4 (=CH_B_), 129.4 (C4′), 129.3 (H_A_C=), 129.1 (C8), 128.9 (C3′/C5′), 127.8 (C6), 124.0 (C5), 123.3 (C4a).

HPLC-ESI^+^-QTOF-MS: *m*/*z* [M + H]^+^ calcd for C_16_H_10_Cl_2_N_2_ [^35^Cl]: 301.0294; found [^35^Cl]: 301.0296.

##### 4.1.2.8 (*E*)-4-(2-Chloro-6-fluorostyryl)quinazoline (5h)

Beige solid; yield: 0.245 g (79%); m.p. 130–131 °C; *R*_f_ = 0.30 (17% ethyl acetate–*n*-heptane).

IR (ATR): *ν̄*_max_ (cm^−1^) 3038 (C_sp^2^_–H), 1625 (C=N), 1539 (C=C_arom_), 1198 (C_sp^2^_–F), 970 (=C–H_*trans*_), 757 (C_sp^2^_–Cl).

NMR ^1^H (400 MHz, CDCl_3_): *δ* = 9.35 (s, 1H, H2), 8.48 (d, *J* = 15.8 Hz, 1H, =CH_B_), 8.28 (dt, *J* = 8.4, 1.2 Hz, 1H, H5), 8.25 (d, *J* = 15.8 Hz, 1H, H_A_C=), 8.06 (dt, *J* = 8.5, 1.2 Hz, 1H, H8), 7.91 (ddd, *J* = 8.4, 6.9, 1.4 Hz, 1H, H7), 7.68 (ddd, *J* = 8.4, 6.9, 1.3 Hz, 1H, H6), 7.32–7.29 (m, 1H, H3′), 7.26 (td, *J* = 8.0, 5.4 Hz, 1H, H4′), 7.12 (dddd, *J* = 11.1, 8.1, 1.6, 0.6 Hz, 1H, H5′).

NMR ^13^C (100 MHz, CDCl_3_): *δ* = 162.1 (d, *J* = 254.5 Hz, C6′), 161.9 (C4), 154.9 (C2), 151.2 (C8a), 136.2 (d, *J* = 5.2 Hz, C2′), 133.7 (C7), 130.1 (d, *J* = 3.0 Hz, =CH_B_), 130.1 (d, *J* = 10.8 Hz, C4′), 129.1 (C8), 127.7 (d, *J* = 13.7 Hz, H_A_C=), 127.6 (C6), 126.1 (d, *J* = 3.6 Hz, C3′), 124.0 (C5), 123.2 (C4a), 123.0 (d, *J* = 14.0 Hz, C1′), 119.9 (d, *J* = 23.5 Hz, C5′).

HPLC-ESI^+^-QTOF-MS: *m*/*z* [M + H]^+^ calcd for C_16_H_10_ClFN_2_ [^35^Cl]: 285.0589; found [^35^Cl]: 285.0591.

##### 4.1.2.9 (*E*)-4-(3,4,5-Trimethoxystyryl)quinazoline (5i)

Yellow solid; yield: 0.258 g (80%); m.p. 181–182 °C; *R*_f_ = 0.20 (33.3% ethyl acetate–*n*-heptane).

IR (ATR): *ν̄*_max_ (cm^−1^) 3040 (C_sp^2^_–H), 1625 (C=N), 1560 (C=C_arom_), 972 (=C–H_*trans*_).

NMR ^1^H (400 MHz, CDCl_3_): *δ* = 9.26 (s, 1H, H2), 8.32 (dt, *J* = 8.4, 1.4 Hz, 1H, H5), 8.21 (d, *J* = 15.4 Hz, 1H, =CH_B_), 8.04 (dd, *J* = 8.4, 1.2 Hz, 1H, H8), 7.89 (ddd, *J* = 8.4, 6.9, 1.4 Hz, 1H, H7), 7.79 (d, *J* = 15.4 Hz, 1H, H_A_C=), 7.66 (ddd, *J* = 8.4, 6.9, 1.4 Hz, 1H, H6), 6.94 (s, 2H, H2′/H6′), 3.95 (s, 6H, 3′-/5′-OCH_3_), 3.91 (s, 3H, 4′-OCH_3_).

NMR ^13^C (100 MHz, CDCl_3_): *δ* = 162.0 (C4), 154.8 (C3′/C5′), 153.6 (C2, C4′), 151.2 (C8a), 140.0 (=CH_B_), 133.6 (C7), 131.4 (C1′), 129.1 (C8), 127.5 (C6), 123.9 (C5), 123.0 (C4a), 119.7 (H_A_C=), 105.4 (C2′/C6′), 61.0 (4′-OCH_3_), 56.3 (3′-/5′-OCH_3_).

HPLC-ESI^+^-QTOF-MS: *m*/*z* [M + H]^+^ calcd for C_19_H_18_N_2_O_3_: 323.1390; found: 323.1391.

Anal. calcd for C_19_H_18_N_2_O_3_: C, 70.79; H, 5.63; N, 8.69. Found: C, 70.76; H, 5.60; N, 8.72.

##### 4.1.2.10 (*E*)-4-(2-(Thiophen-2-yl)vinyl)quinazoline (5j)

Yellow solid; yield: 0.191 g (80%); m.p. 74–75 °C; *R*_f_ = 0.30 (25% ethyl acetate–*n*-heptane).

IR (ATR): *ν̄*_max_ (cm^−1^) 3057 (C_sp^2^_–H), 1617 (C=N), 1534 (C=C_arom_), 948 (=C–H_*trans*_).

NMR ^1^H (400 MHz, CDCl_3_): *δ* = 9.25 (s, 1H, H2), 8.42 (d, *J* = 15.0 Hz, 1H, =CH_B_), 8.27 (d, *J* = 8.4 Hz, 1H, H5), 8.03 (d, *J* = 8.3 Hz, 1H, H8), 7.89 (ddd, *J* = 8.3, 7.0, 1.3 Hz, 1H, H7), 7.69 (d, *J* = 15.0 Hz, 1H, H_A_C=), 7.67–7.64 (m, 1H, H6), 7.40 (d, *J* = 5.1 Hz, 1H, H5′), 7.37 (d, *J* = 3.6 Hz, H3′), 7.11–7.09 (m, 1H, H4′).

NMR ^13^C (100 MHz, CDCl_3_): *δ* = 161.8 (C4), 154.8 (C2), 151.1 (C8a), 141.4 (C2′), 133.6 (C7), 132.5 (=CH_B_), 130.7 (C3′), 129.0 (C8), 128.3 (C4′), 127.8 (C5′), 127.5 (C6), 123.9 (C5), 122.9 (C4a), 119.4 (H_A_C=).

HPLC-ESI^+^-QTOF-MS: *m*/*z* [M + H]^+^ calcd for C_14_H_10_N_2_S: 239.0637; found: 239.0640.

Anal. calcd for C_14_H_10_N_2_S: C, 70.56; H, 4.23; N, 11.76; S, 13.45. Found: C, 70.54; H, 4.22; N, 11.73; S, 13.41.

## Data availability

The data supporting this article have been included as part of the ESI† (^1^H and ^13^C NMR spectra of compounds 3 and 5). Growth inhibitory percentage (GI%) of compounds 3 over all tested *in vitro* tumor cell lines (Table 1S†). Growth inhibitory percentage (GI%) of compounds 5 over all tested *in vitro* tumor cell lines (Table 2S†). Crystallographic data for compounds 3d, 5b and 5f have been deposited at CCDC with deposition numbers 2349436, 2349524, and 2349522, respectively.†

## Author contributions

All authors declare that they have all participated in the execution and analysis of the paper and approved the final version.

## Conflicts of interest

There are no conflicts to declare.

## Supplementary Material

RA-014-D4RA03702B-s001

RA-014-D4RA03702B-s002
