# Supplementary material for: Alternative and efficient one-pot three-component synthesis of substituted 2-aryl-4-styrylquinazolines/4-styrylquinazolines from synthetically available 1-(2-aminophenyl)-3-arylprop-2-en-1-ones: characterization and evaluation of their antiproliferative activities
Source: RSC Adv. 2024 Jul 2;14(29):20951–65. doi: 10.1039/d4ra03702b (PMC11218040; doi:10.1039/d4ra03702b)
Supplement: RA-014-D4RA03702B-s001 [file RA-014-D4RA03702B-s001.pdf]

Electronic Supplementary Material (ESI) for RSC Advances.
This journal is © The Royal Society of Chemistry 2024

An alternative and efficient one-pot three-component synthesis of substituted 2-aryl-4-styrylquinazolines/4-styrylquinazolines from synthetically available 1-(2-aminophenyl)-3-arylprop-2-en-1-ones. Characterization and evaluation of their antiproliferative activity

Diego Fernando Rodríguez,[1] Kelly Johanna Lipez,[1] Elena Stashenko,[2] Iván Díaz,[3] Justo Cobo,[3] Alirio Palma,[1*]

[1] *Laboratorio de Síntesis Orgánica, Escuela de Química, Universidad Industrial de Santander, AA 678 Bucaramanga, Colombia.*
[2] *National Research Center for the Agroindustrialization of Aromatic and Medicinal Tropical Species (CENIVAM), Universidad Industrial de Santander.*
[3] *Departamento de Química Inorgánica y Orgánica, Universidad de Jaén, España.*
e-mail: apalma@uis.edu.co

Supplementary Information

(*E*)–2–Phenyl–4–styrylquinazoline (3a)

^{1}H NMR spectrum of 3a

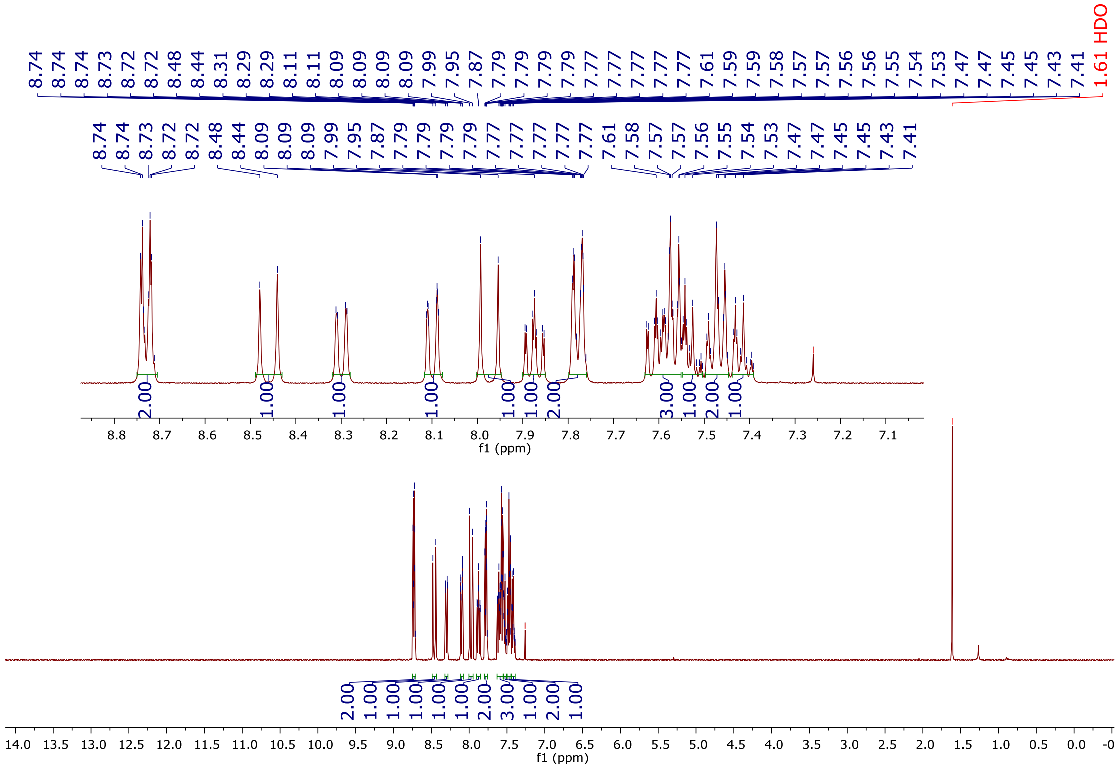

^{13}C NMR spectrum of 3a

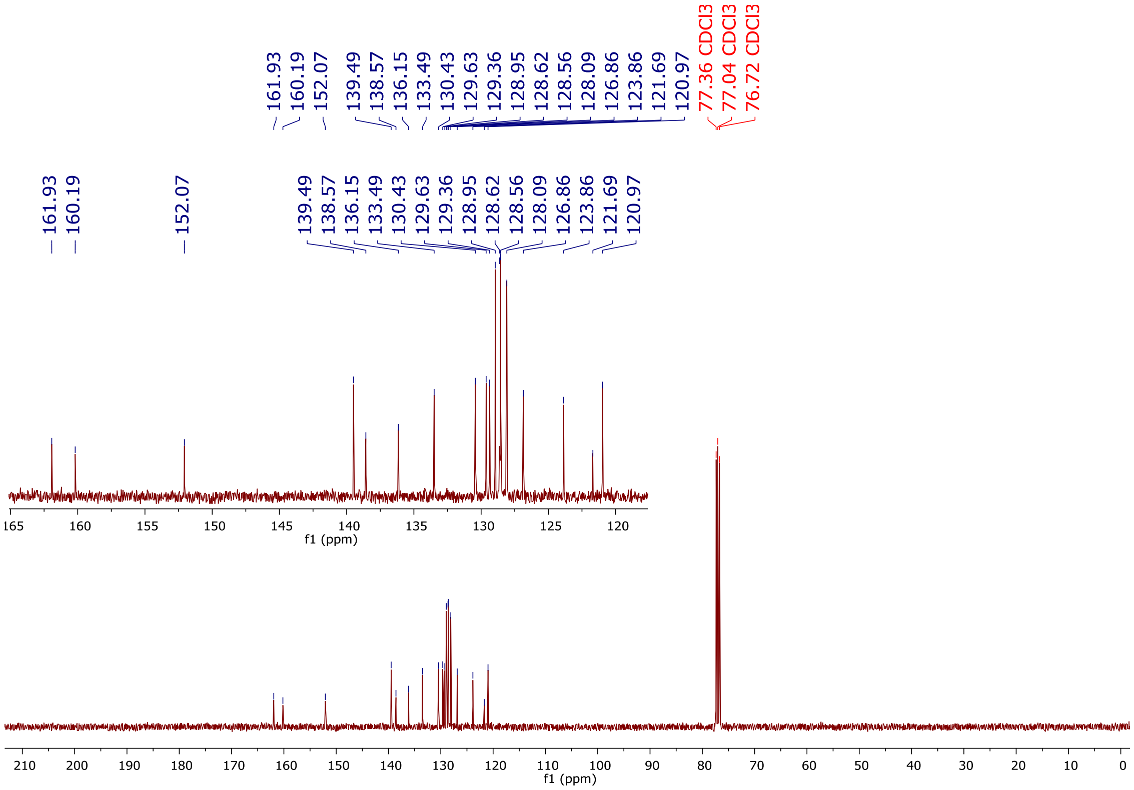

HRMS spectrum of 3a

MS Zoomed Spectrum

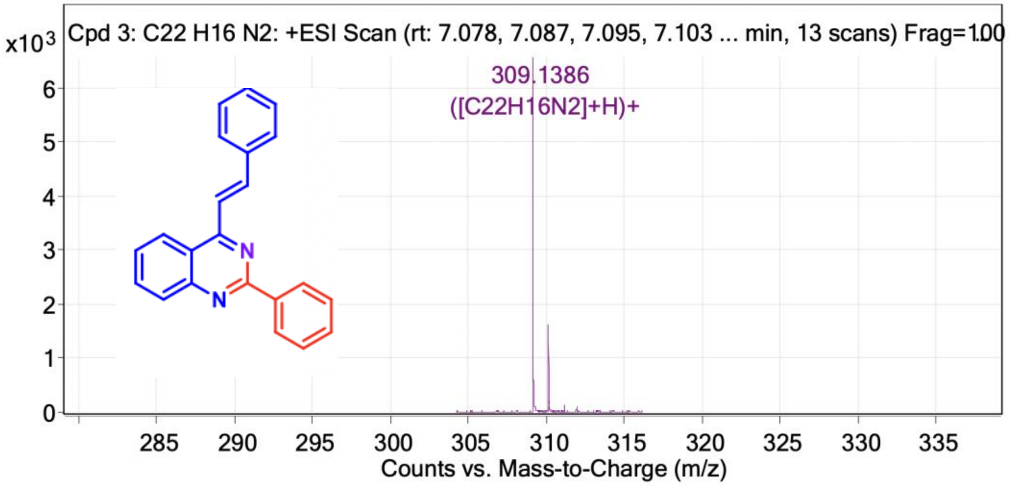

MS Spectrum Peak List

m/z	Calc *m/z*	Diff(ppm)	z	Abund	Formula	Ion
309.1386	309.1386	0.14	1	6573.96	C22H16N2	(M+H)+
310.142	310.1418	-0.48	1	1670.56	C22H16N2	(M+H)+
311.1408	311.145	13.56	1	113.33	C22H16N2	(M+H)+

--- End Of Report ---

(*E*)–4–Styryl–2–(*m*–tolyl)quinazoline (3b)

^{1}H NMR spectrum of 3b

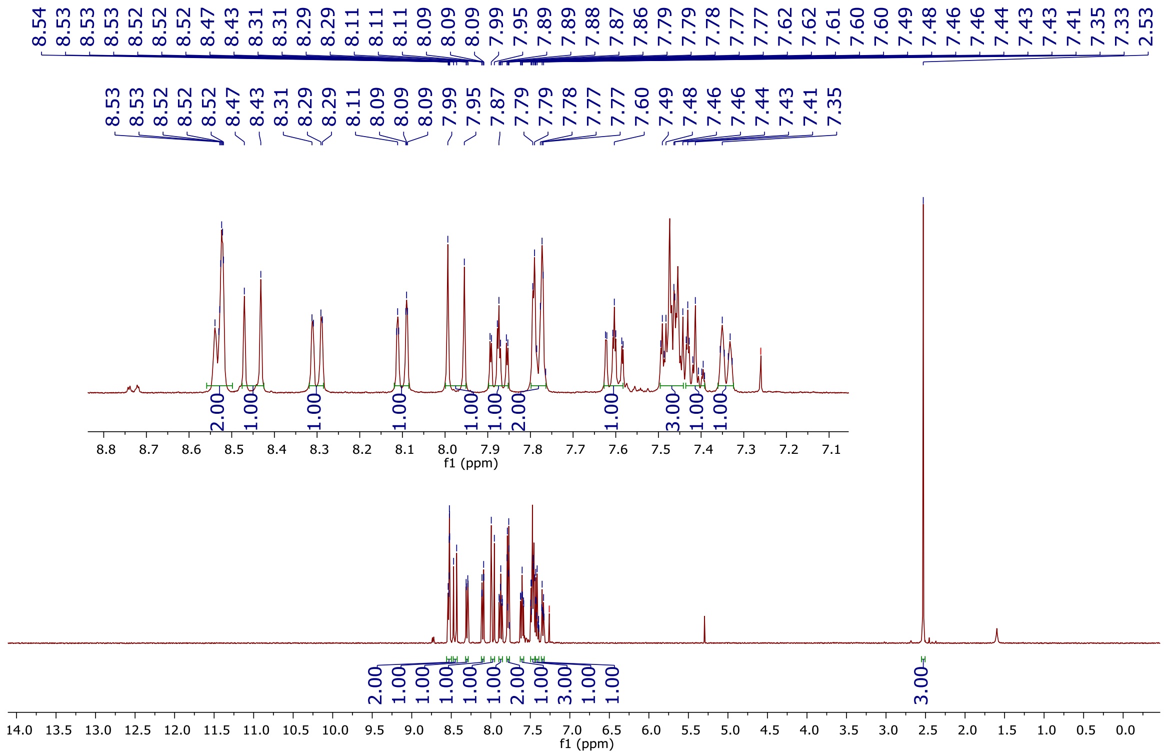

^{13}C NMR spectrum of 3b

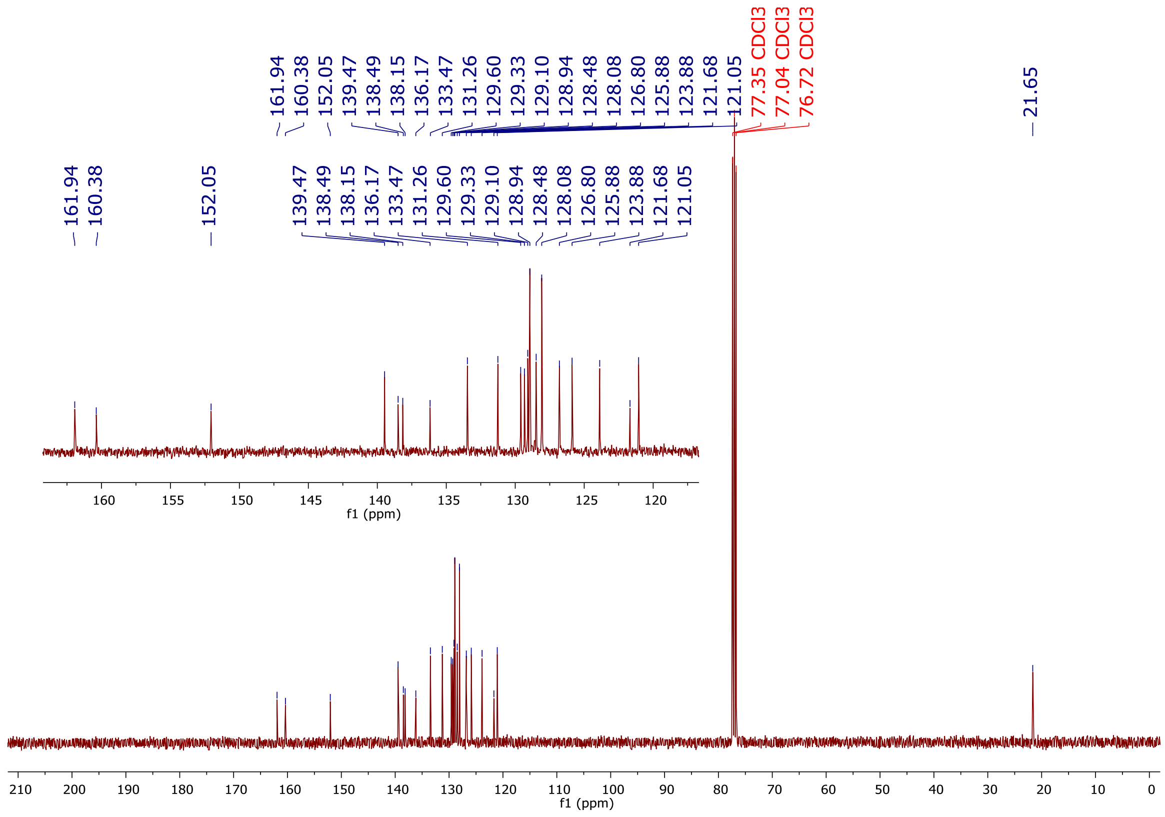

HRMS spectrum of 3b

MS Zoomed Spectrum

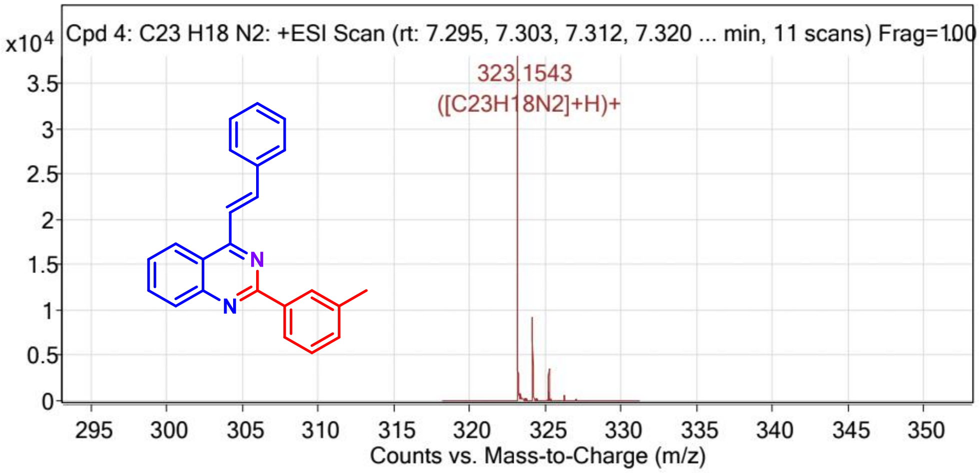

MS Spectrum Peak List

m/z	Calc *m/z*	Diff(ppm)	z	Abund	Formula	Ion
323.1543	323.1543	-0.08	1	38167.41	C23H18N2	(M+H)+
324.1573	324.1575	0.68	1	9464.11	C23H18N2	(M+H)+
325.1631	325.1607	-7.36	1	780.06	C23H18N2	(M+H)+
326.167	326.1639	-9.73	1	27.8	C23H18N2	(M+H)+

--- End Of Report ---

(*E*)–2–(3–Nitrophenyl)–4–styrylquinazoline (3c)

^{1}H NMR spectrum of 3c

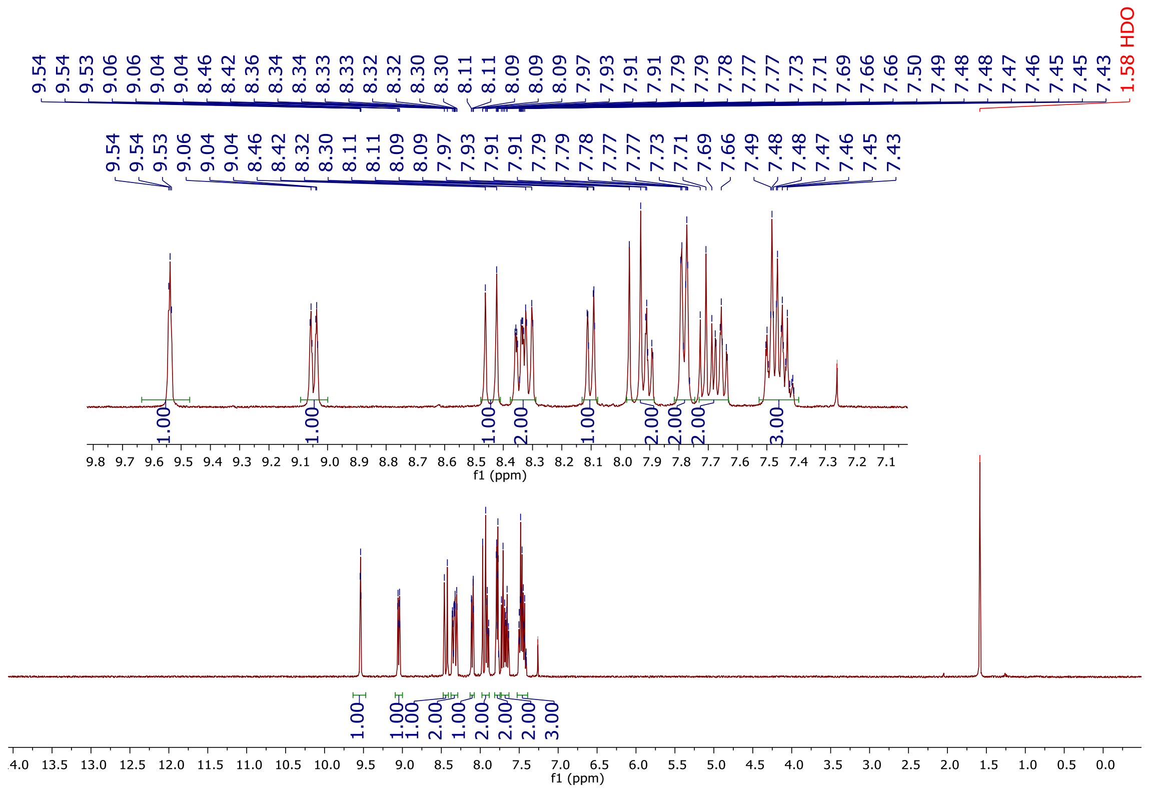

^{13}C NMR spectrum of 3c

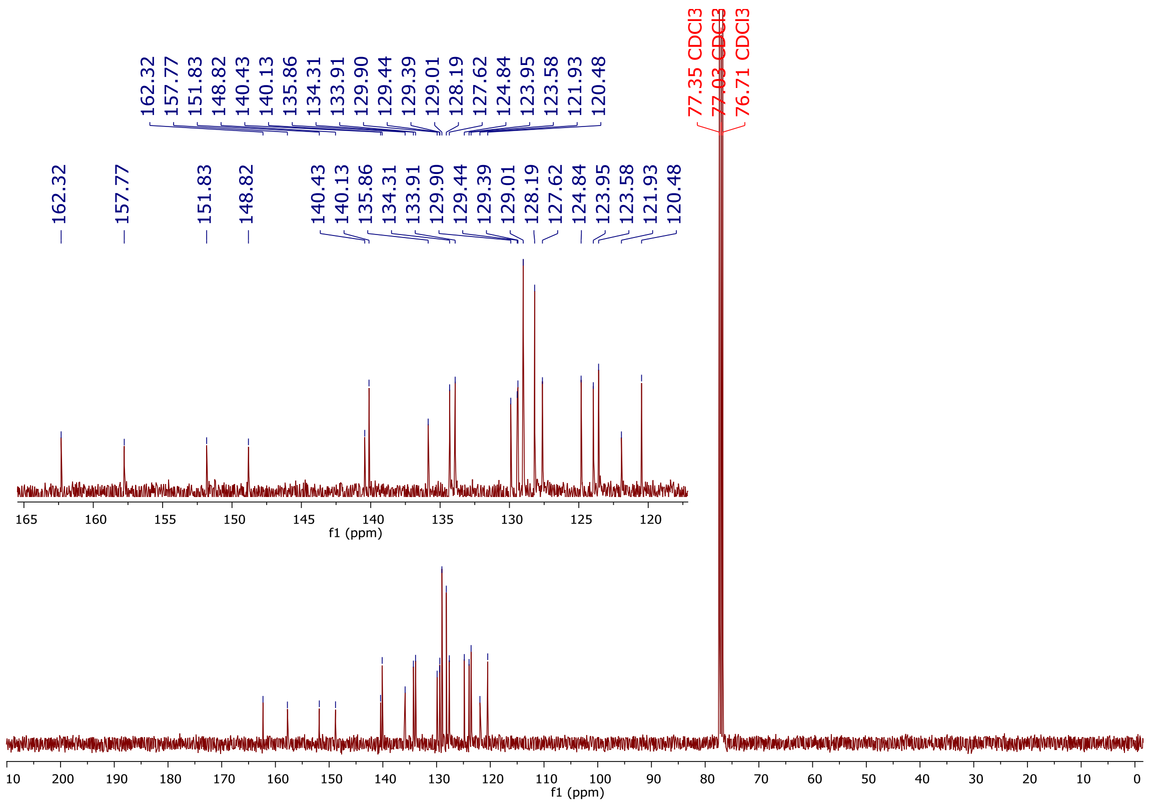

HRMS spectrum of 3c

MS Zoomed Spectrum

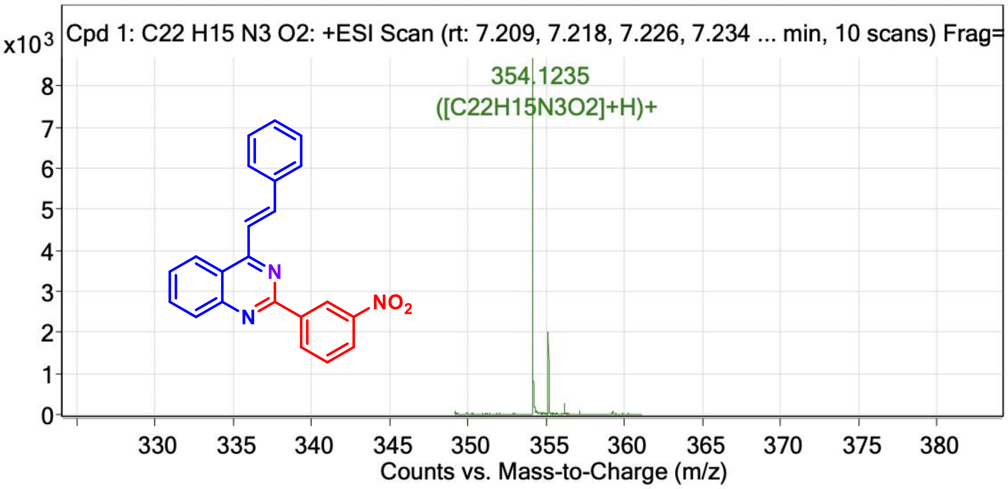

MS Spectrum Peak List

m/z	Calc *m/z*	Diff(ppm)	z	Abund	Formula	Ion
354.1235	354.1237	0.51	1	8798.42	C22H15N3O2	(M+H)+
355.1274	355.1268	-1.79	1	2072.13	C22H15N3O2	(M+H)+
356.1313	356.1297	-4.51	1	269.41	C22H15N3O2	(M+H)+

--- End Of Report ---

(*E*)–4–Styryl–2–(4–(trifluoromethyl)phenyl)quinazoline (3d)

^{1}H NMR spectrum of 3d

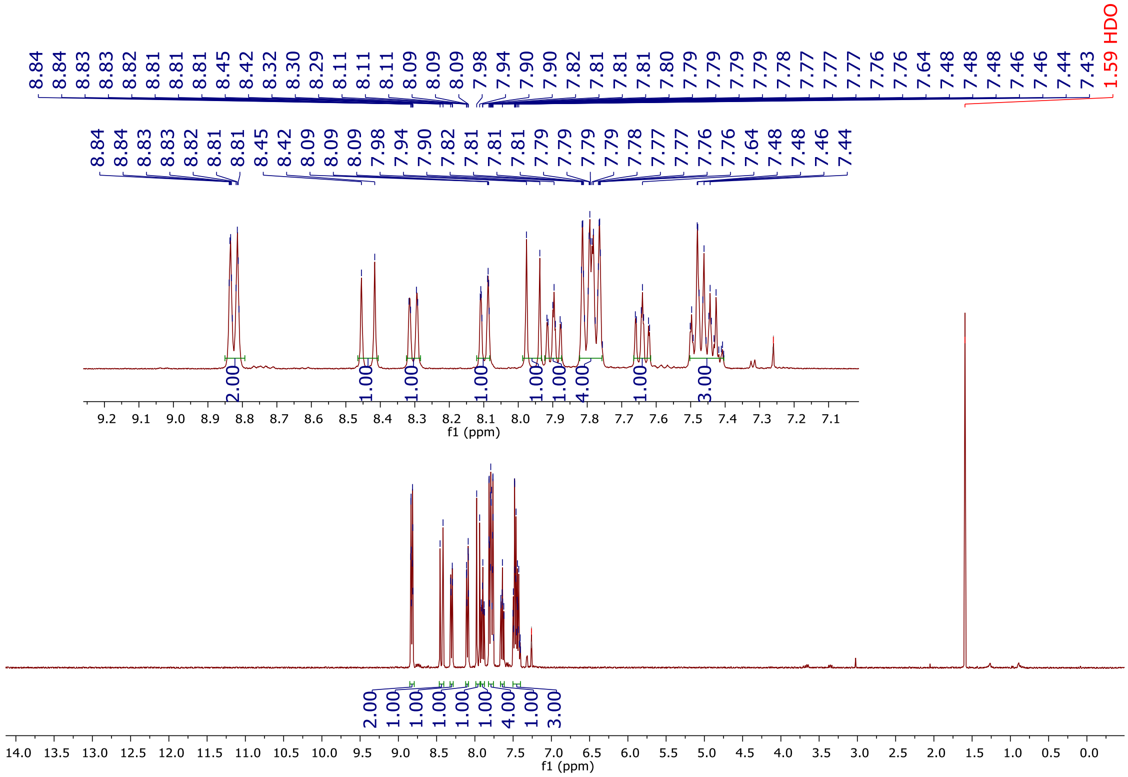

^{13}C NMR spectrum of 3d

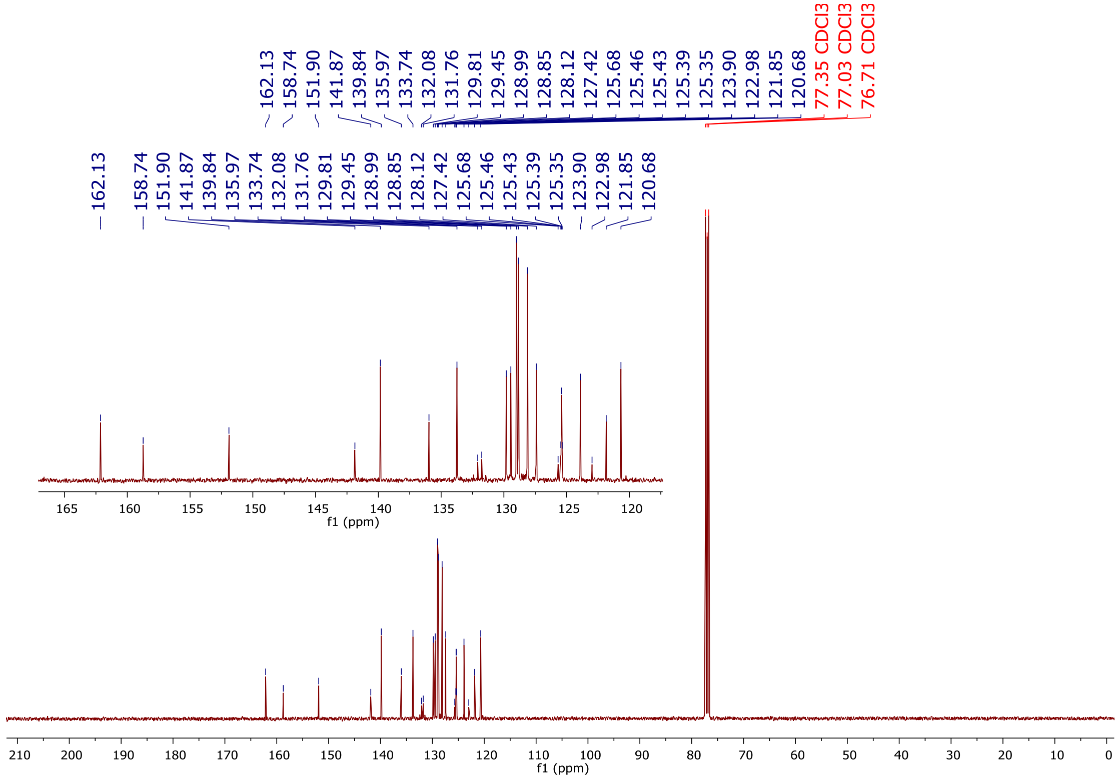

HRMS spectrum of 3d

MS Zoomed Spectrum

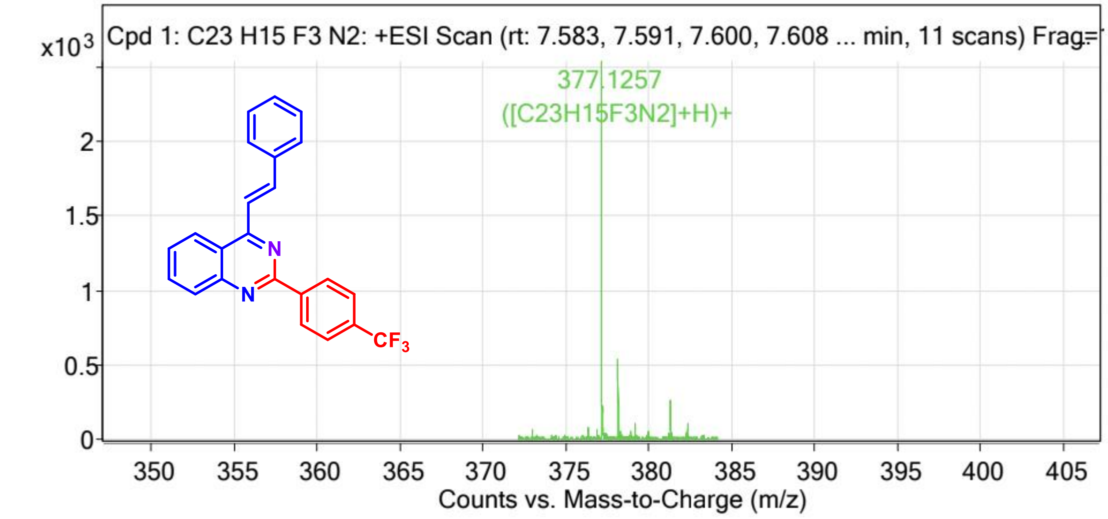

MS Spectrum Peak List

m/z	Calc *m/z*	Diff(ppm)	z	Abund	Formula	Ion
377.1257	377.126	0.77	1	2647.16	C23H15F3N2	(M+H)+
378.1296	378.1292	-0.98	1	622.54	C23H15F3N2	(M+H)+
379.134	379.1324	-4.22	1	95.75	C23H15F3N2	(M+H)+

--- End Of Report ---

(*E*)–4–Styryl–2–(3,4,5–trimetoxyphenyl)quinazoline (3e)

^{1}H NMR spectrum of 3e

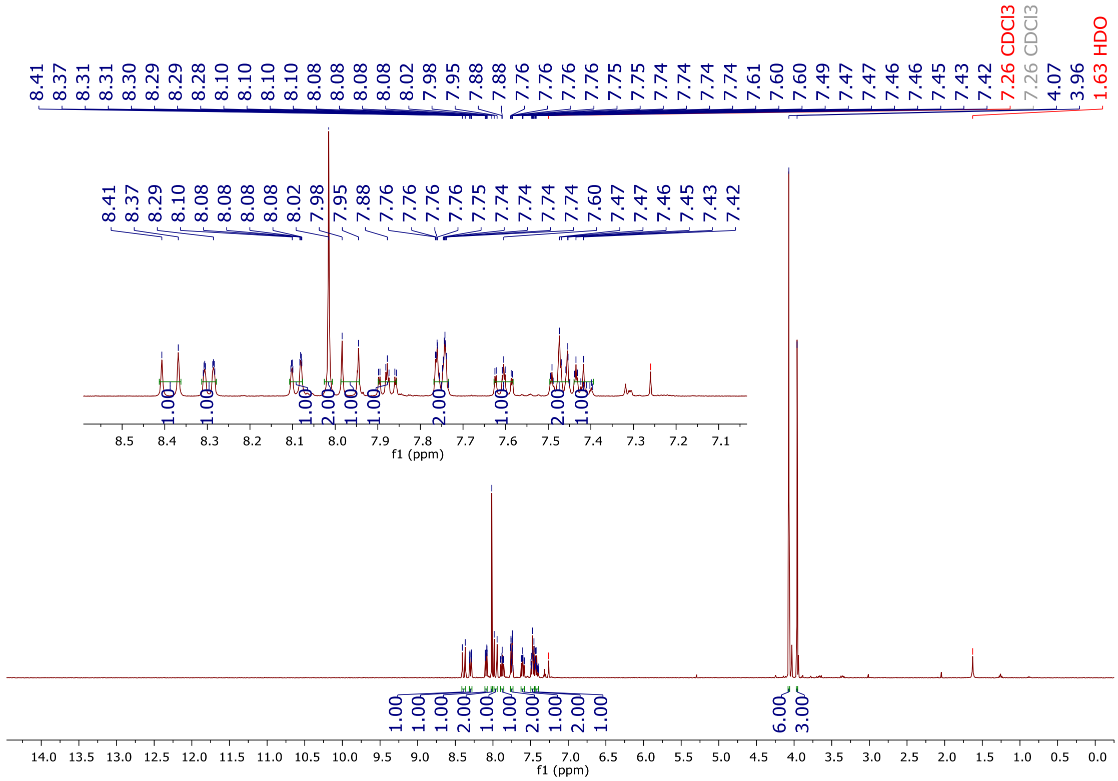

^{13}C NMR spectrum of 3e

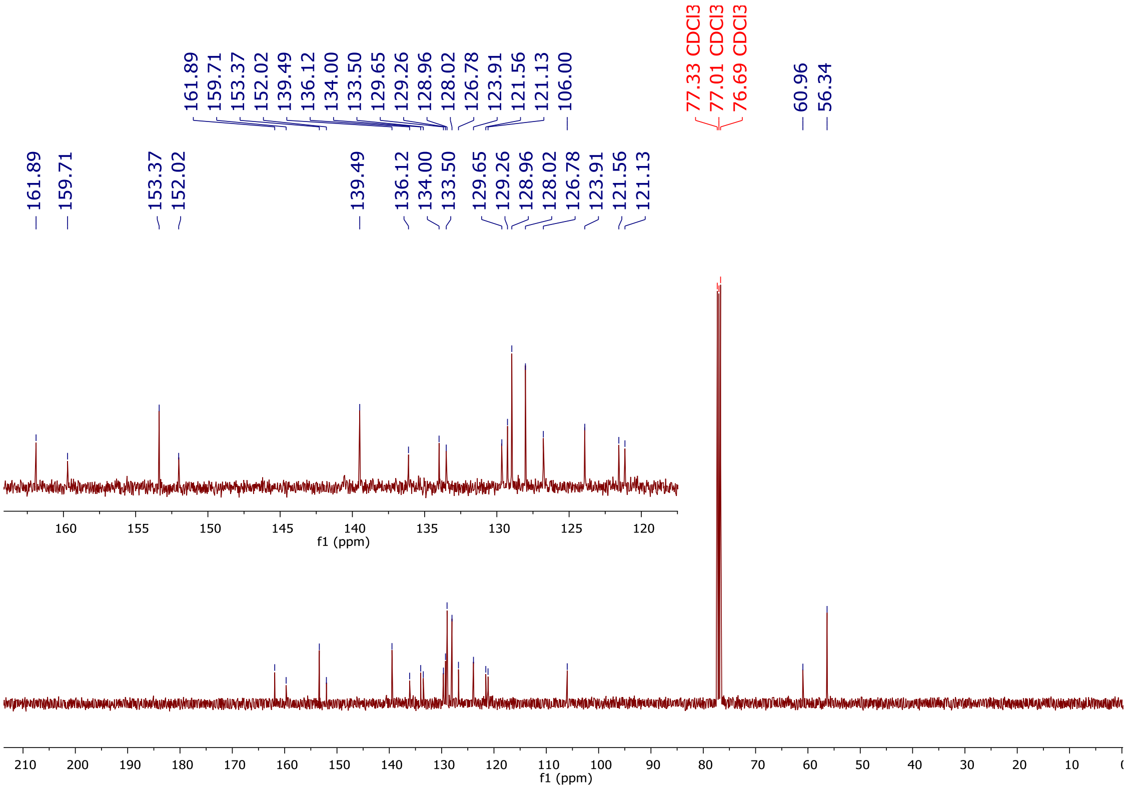

Chromatogram and HRMS spectrum of 3e

c:\xcalibur\data\220614ag\992734-31-eb 06/15/22 21:26:18

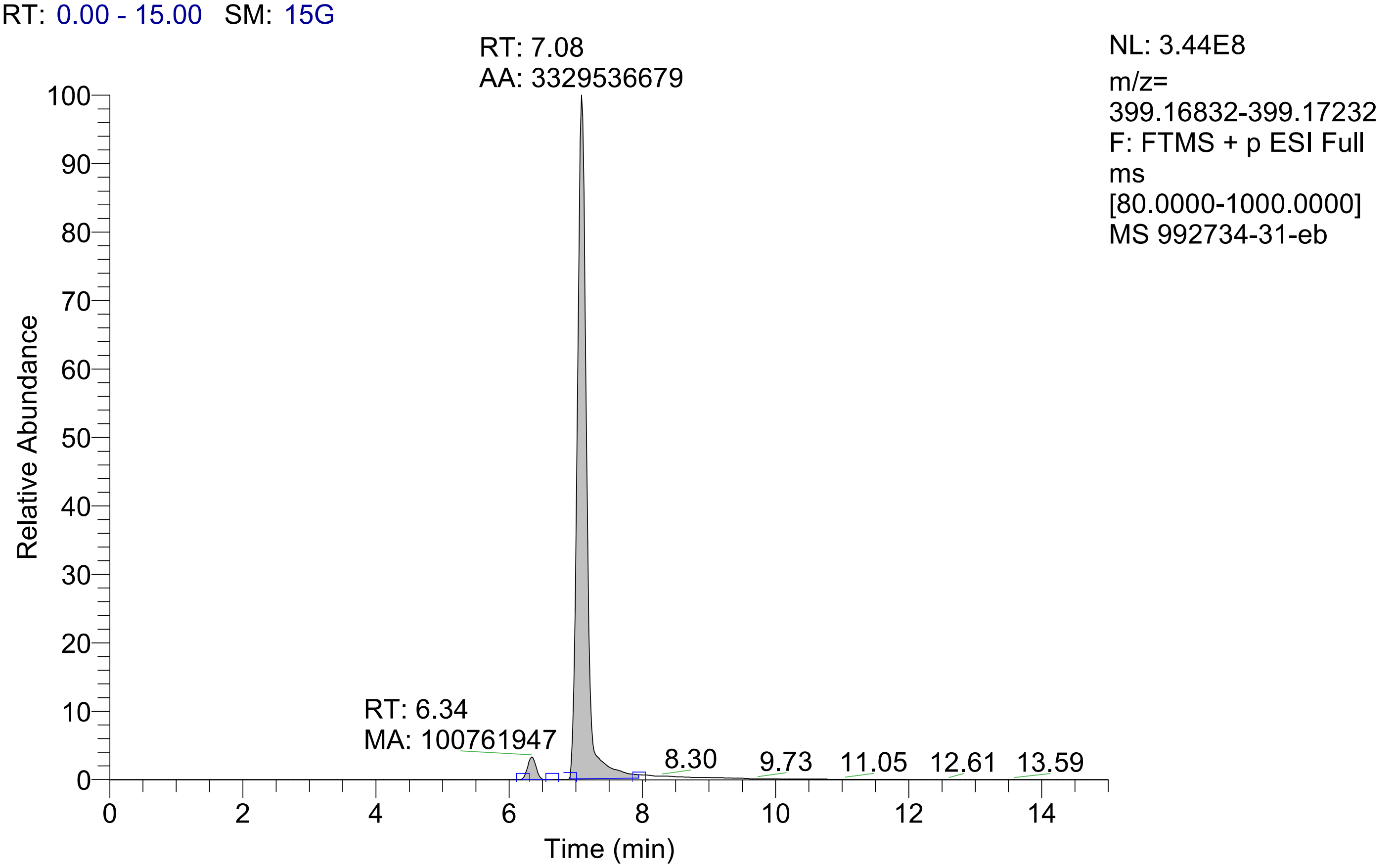

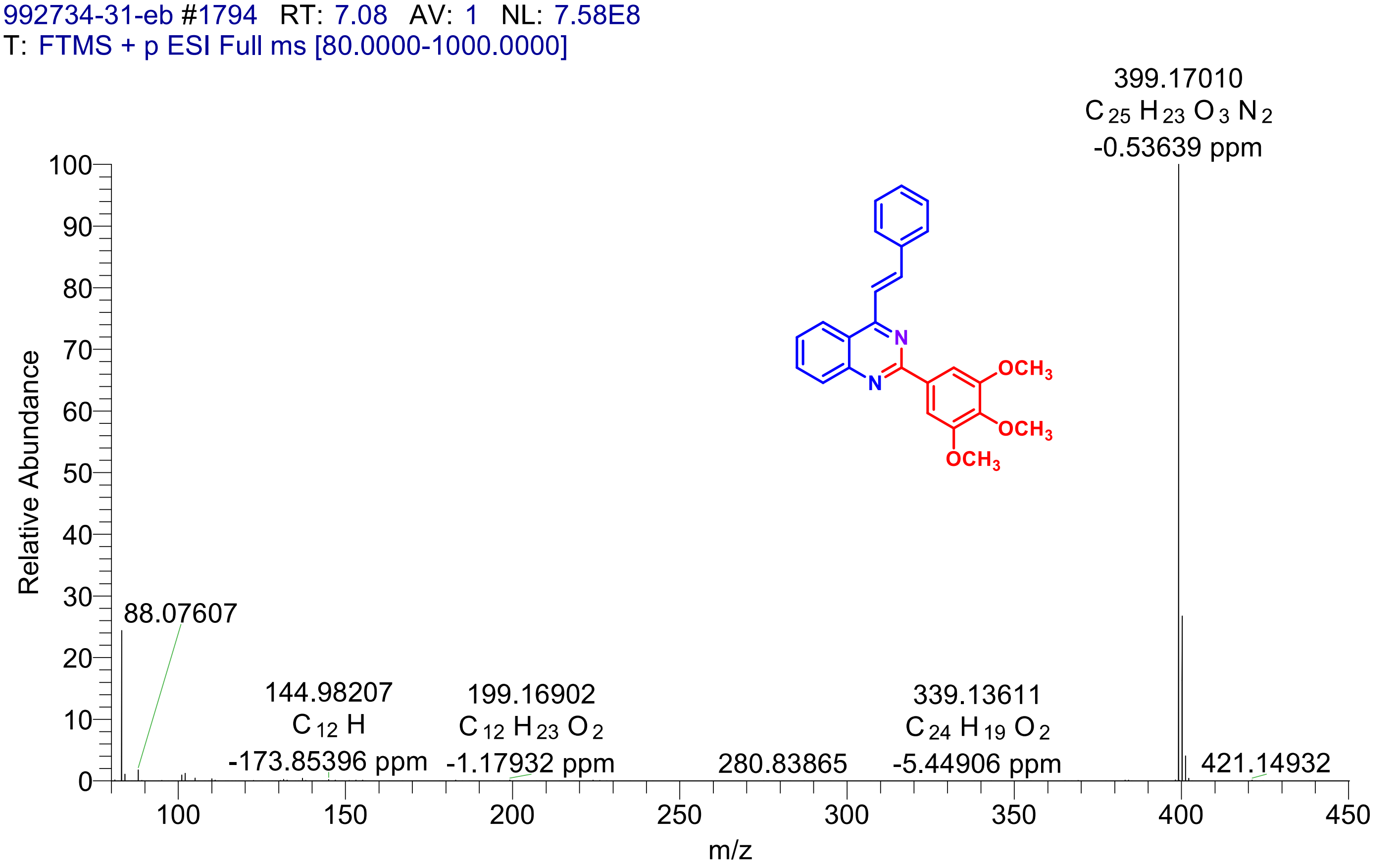

(*E*)–4–Styryl–2–(5–methylfuran–2–yl)quinazoline (3f)

^{1}H NMR spectrum of 3f

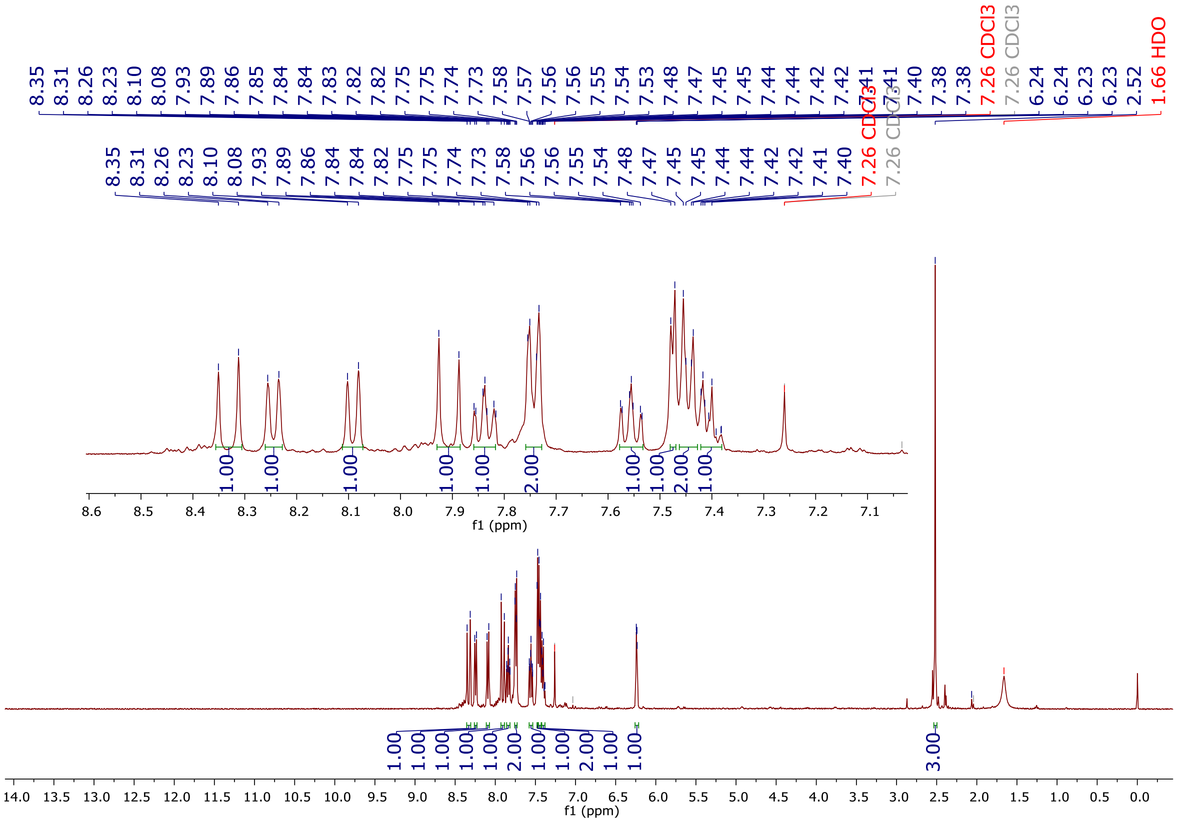

^{13}C NMR spectrum of 3f

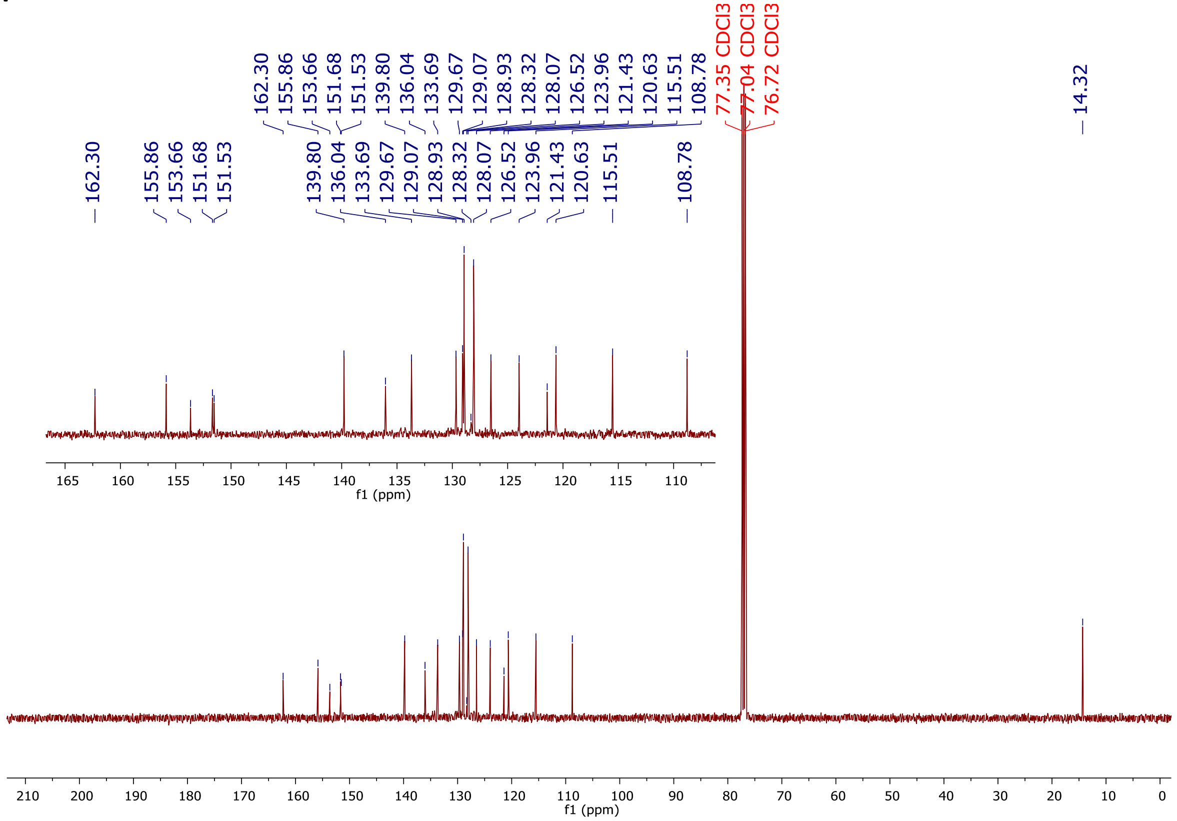

Chromatogram and HRMS spectra of 3f

c:\xcalibur\data\220614ag\992734-32-eb 06/15/22 22:19:13

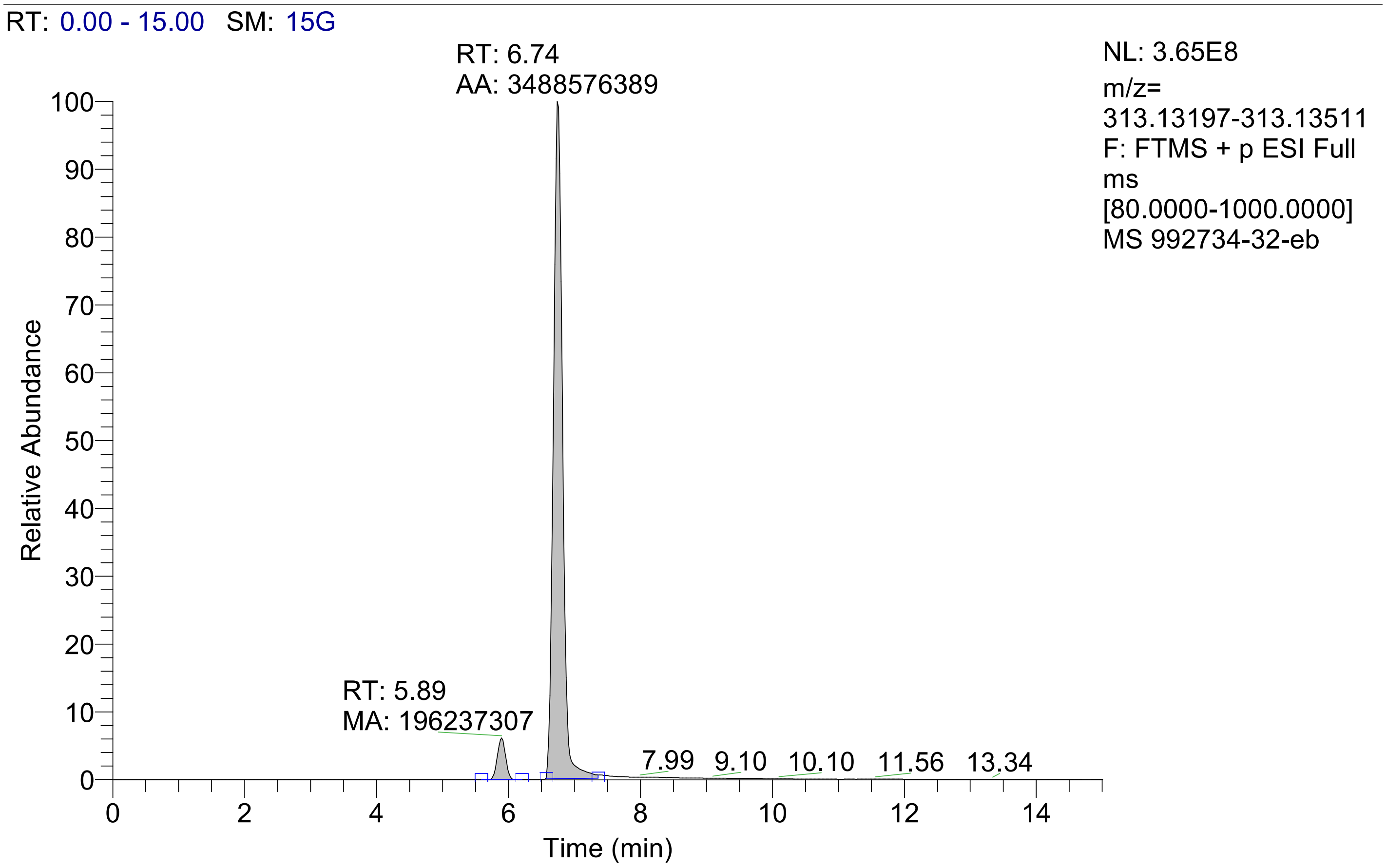

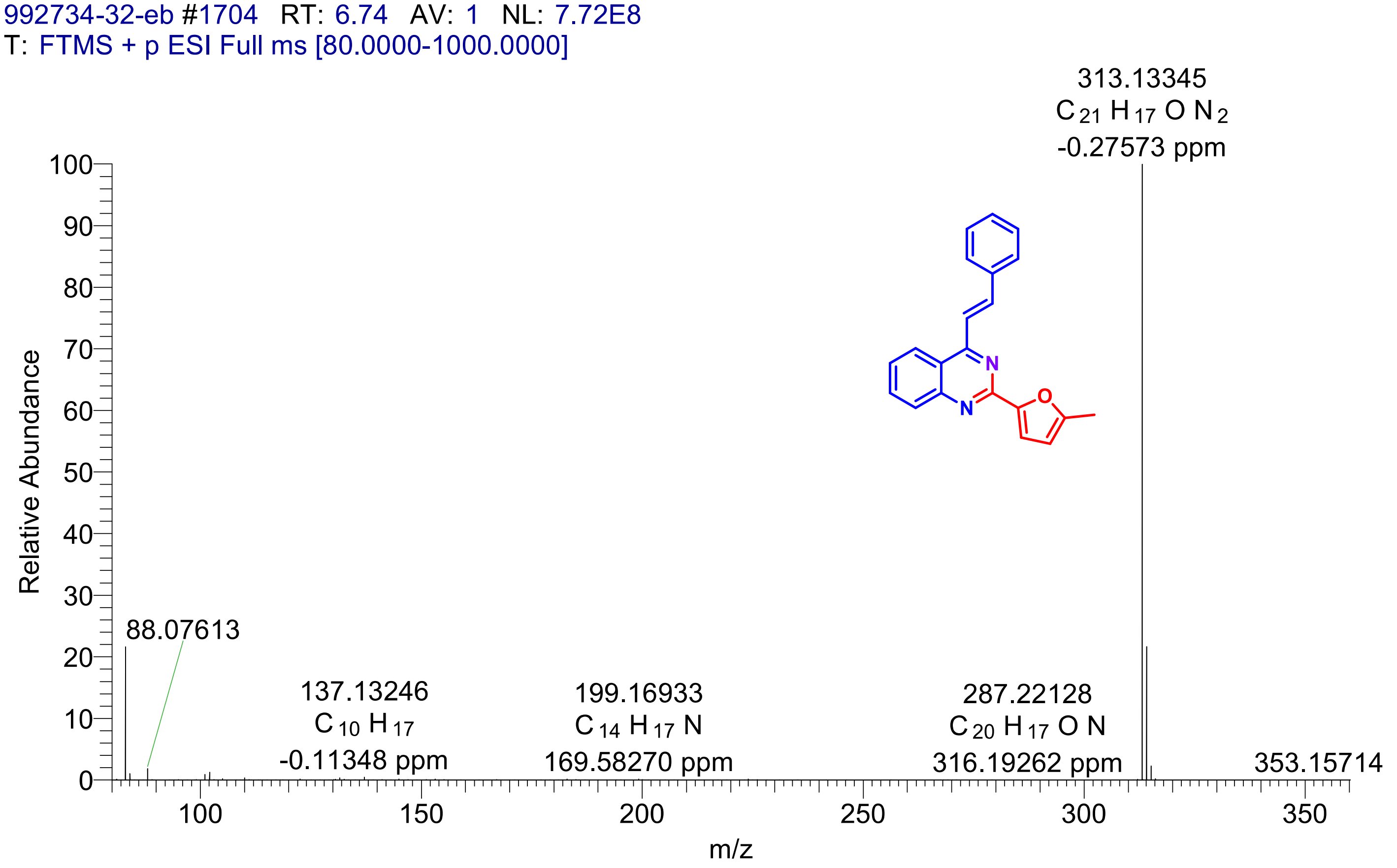

(*E*)–4–Styryl–2–(furan–3–yl)quinazoline (3g)

^{1}H NMR spectrum of 3g

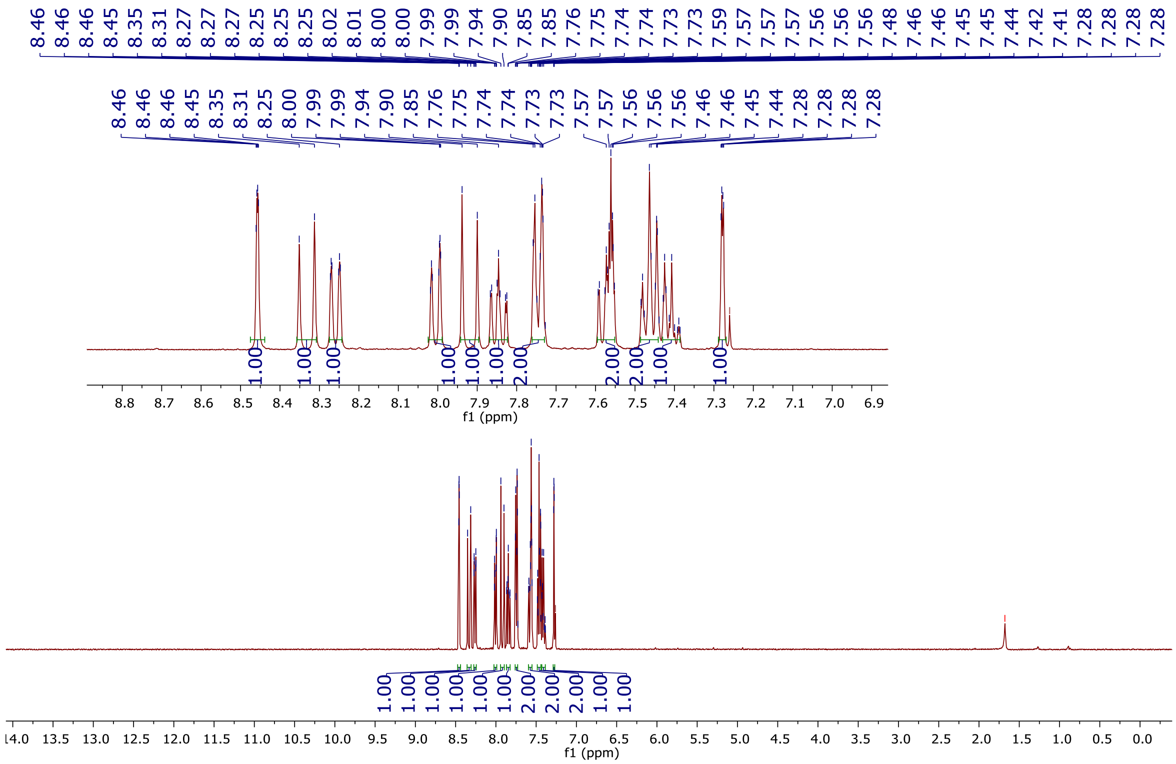

^{13}C NMR spectrum of 3g

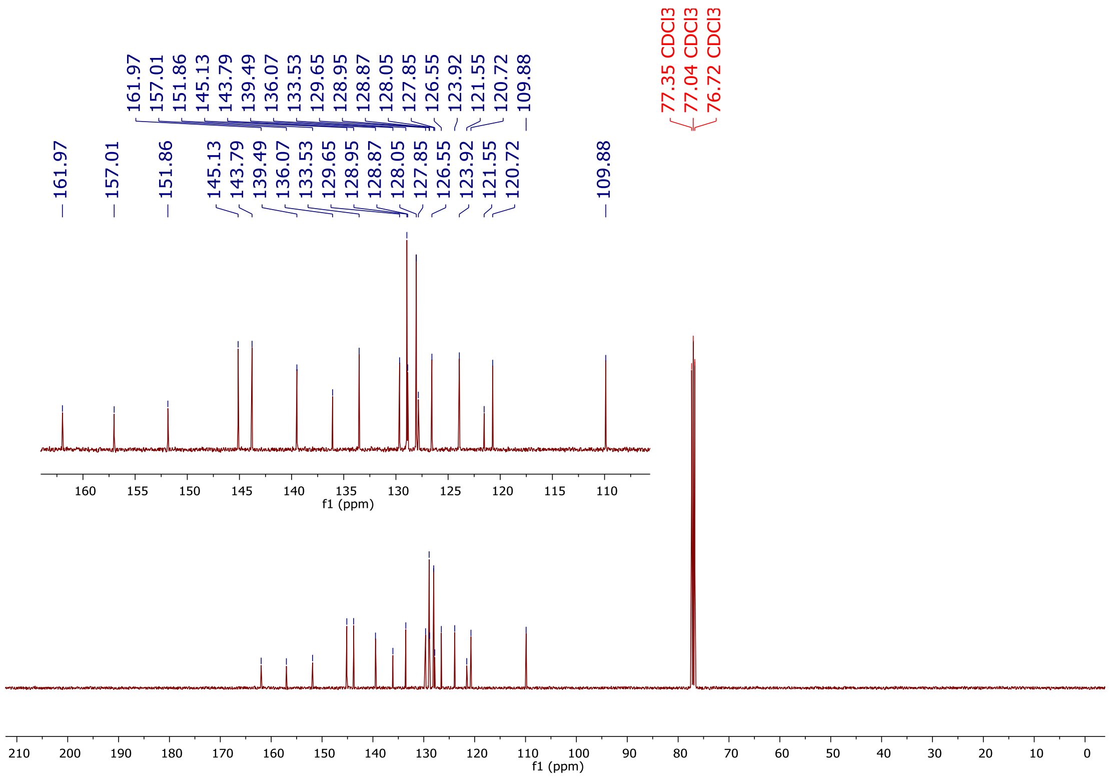

HRMS spectrum of 3g

MS Zoomed Spectrum

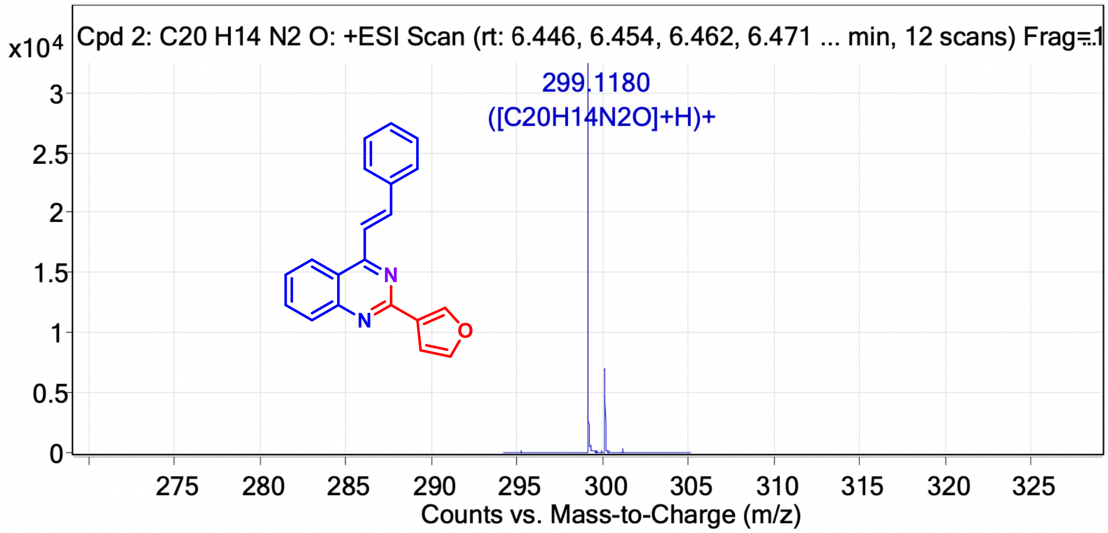

MS Spectrum Peak List

m/z	Calc *m/z*	Diff(ppm)	z	Abund	Formula	Ion
299.118	299.1179	-0.41	1	32864.48	C20H14N2O	(M+H)+
300.1212	300.1211	-0.43	1	7266.67	C20H14N2O	(M+H)+

--- End Of Report ---

(*E*)–4–(4–Chlorostyryl)–2–phenylquinazoline (3i)

^{1}H NMR spectrum of 3i

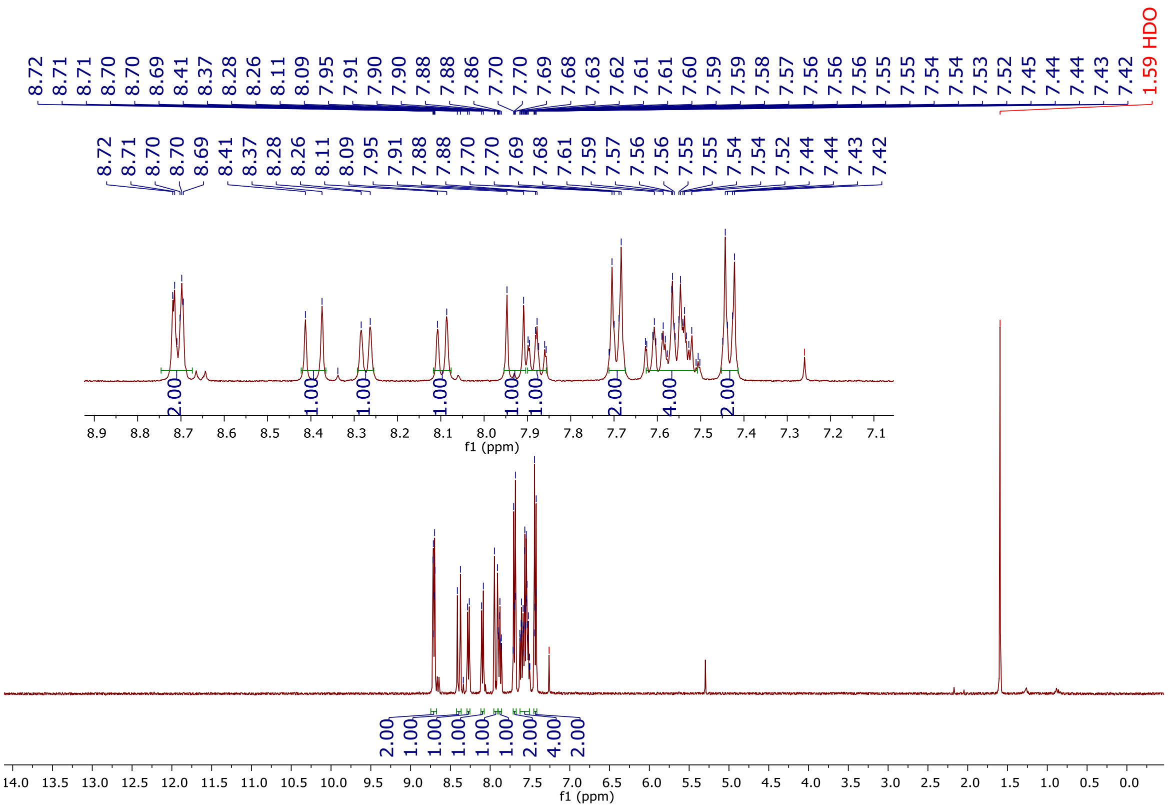

^{13}C NMR spectrum of 3i

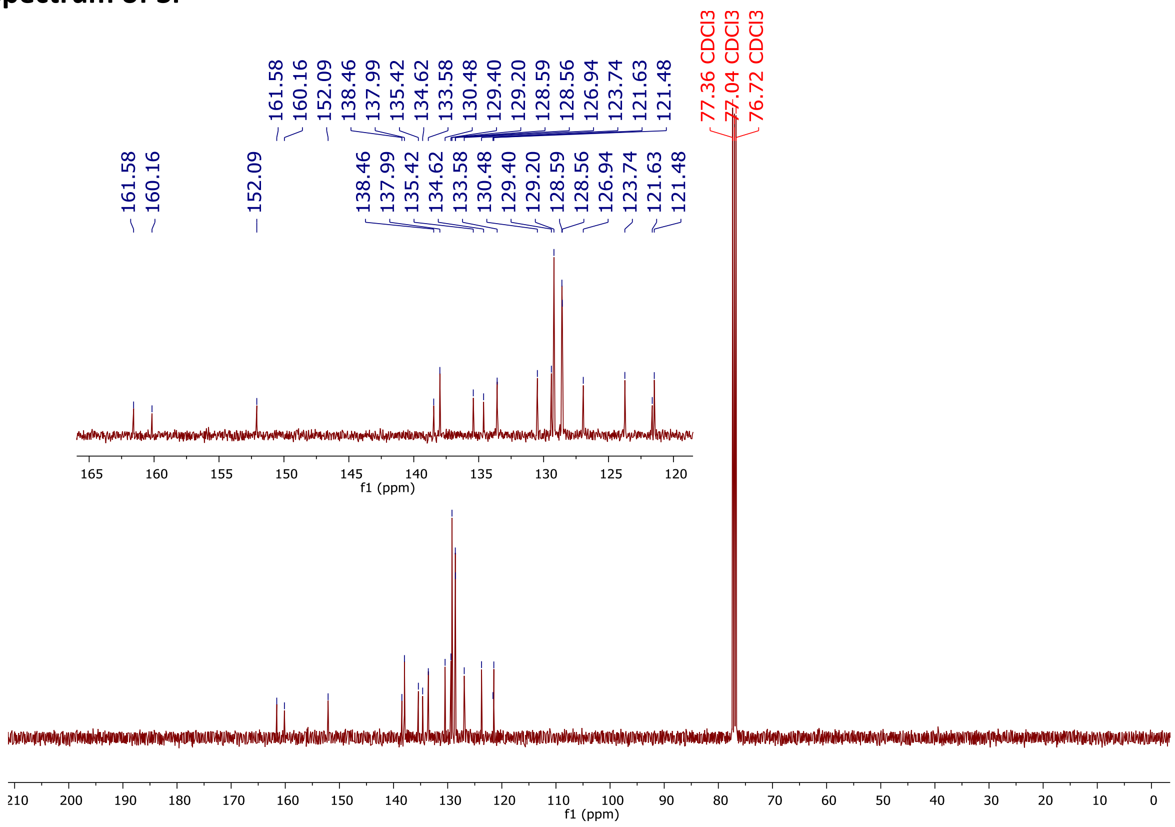

HRMS spectrum of 3i

MS Zoomed Spectrum

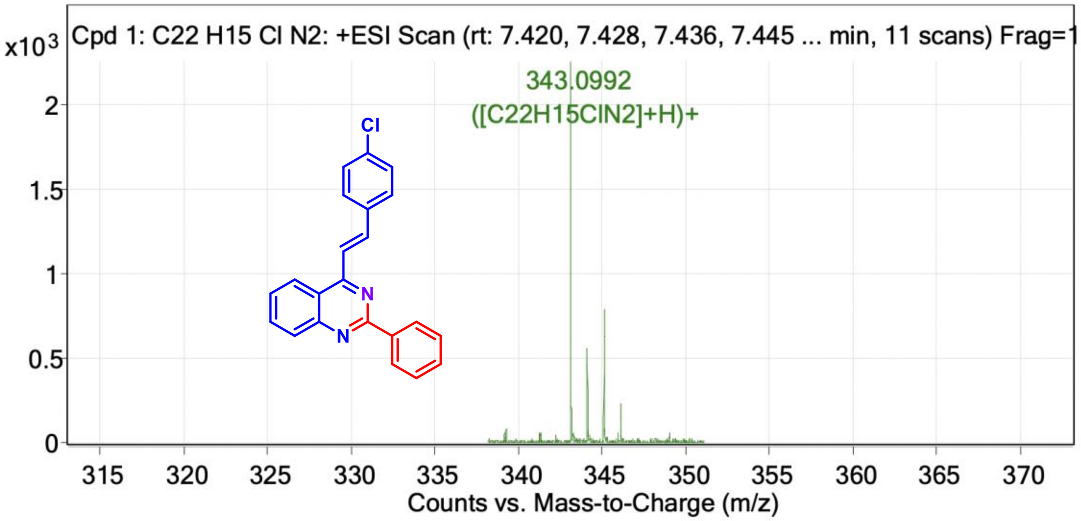

MS Spectrum Peak List

m/z	Calc *m/z*	Diff(ppm)	z	Abund	Formula	Ion
343.0992	343.0997	1.2	1	2346.7	C22H15ClN2	(M+H)+
344.1035	344.1028	-1.97	1	625.89	C22H15ClN2	(M+H)+
345.0979	345.0975	-1.11	1	854.34	C22H15ClN2	(M+H)+
346.099	346.1001	3.36	1	191.5	C22H15ClN2	(M+H)+

--- End Of Report ---

(*E*)–4–(4–Trifluoromethylstyryl)–2–phenylquinazoline (3j)

^{1}H NMR spectrum of 3j

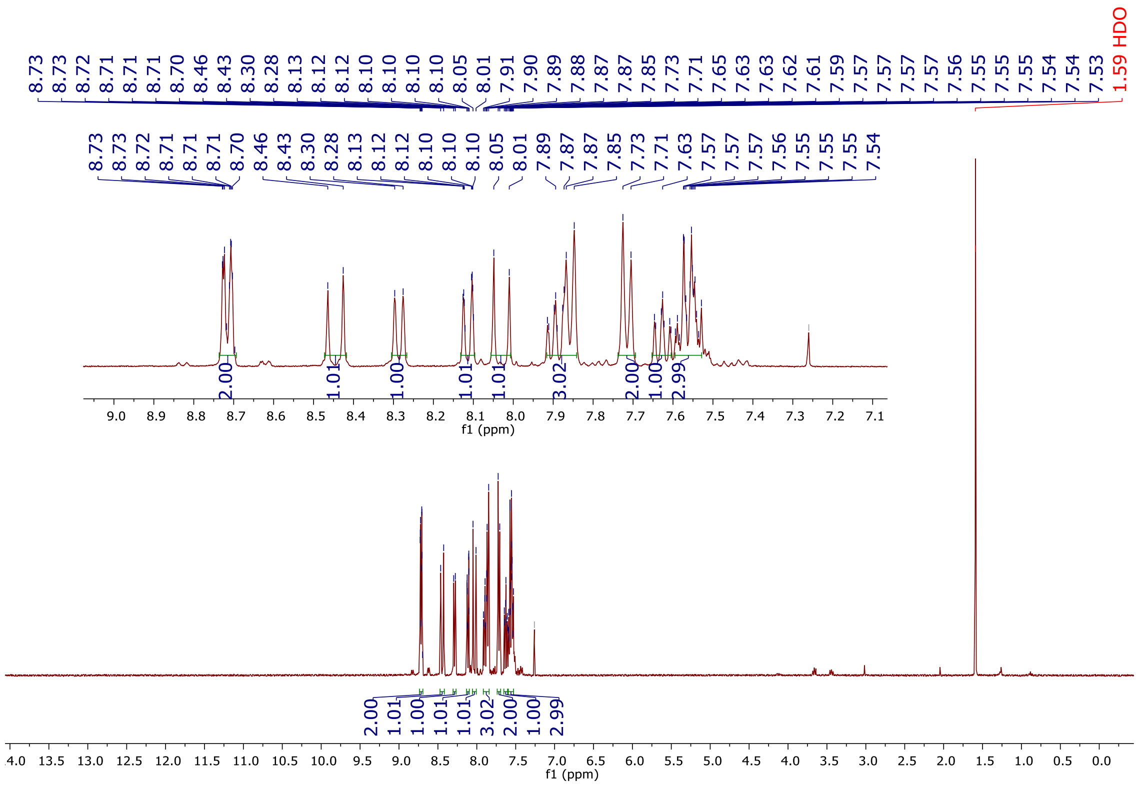

^{13}C NMR spectrum of 3j

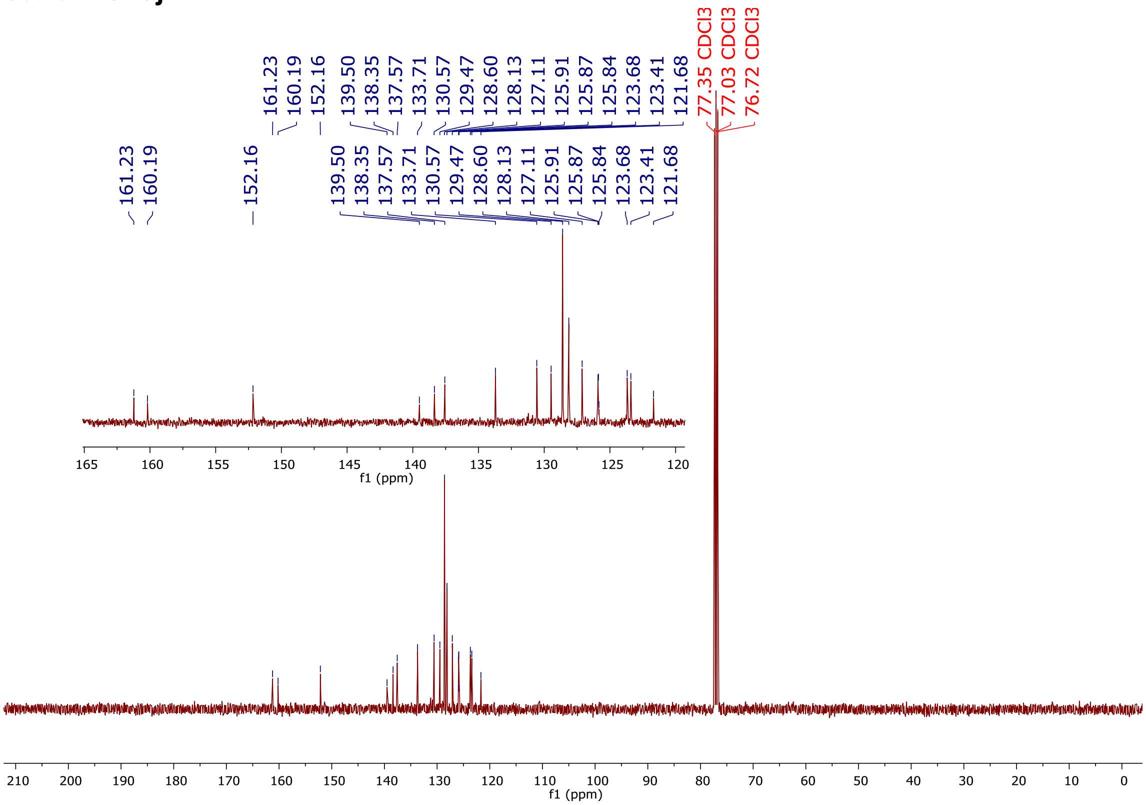

Chromatogram and HRMS spectrum of 3j

c:\xcalibur\...\221101ag\993103-13-eb 11/02/22 03:00:58

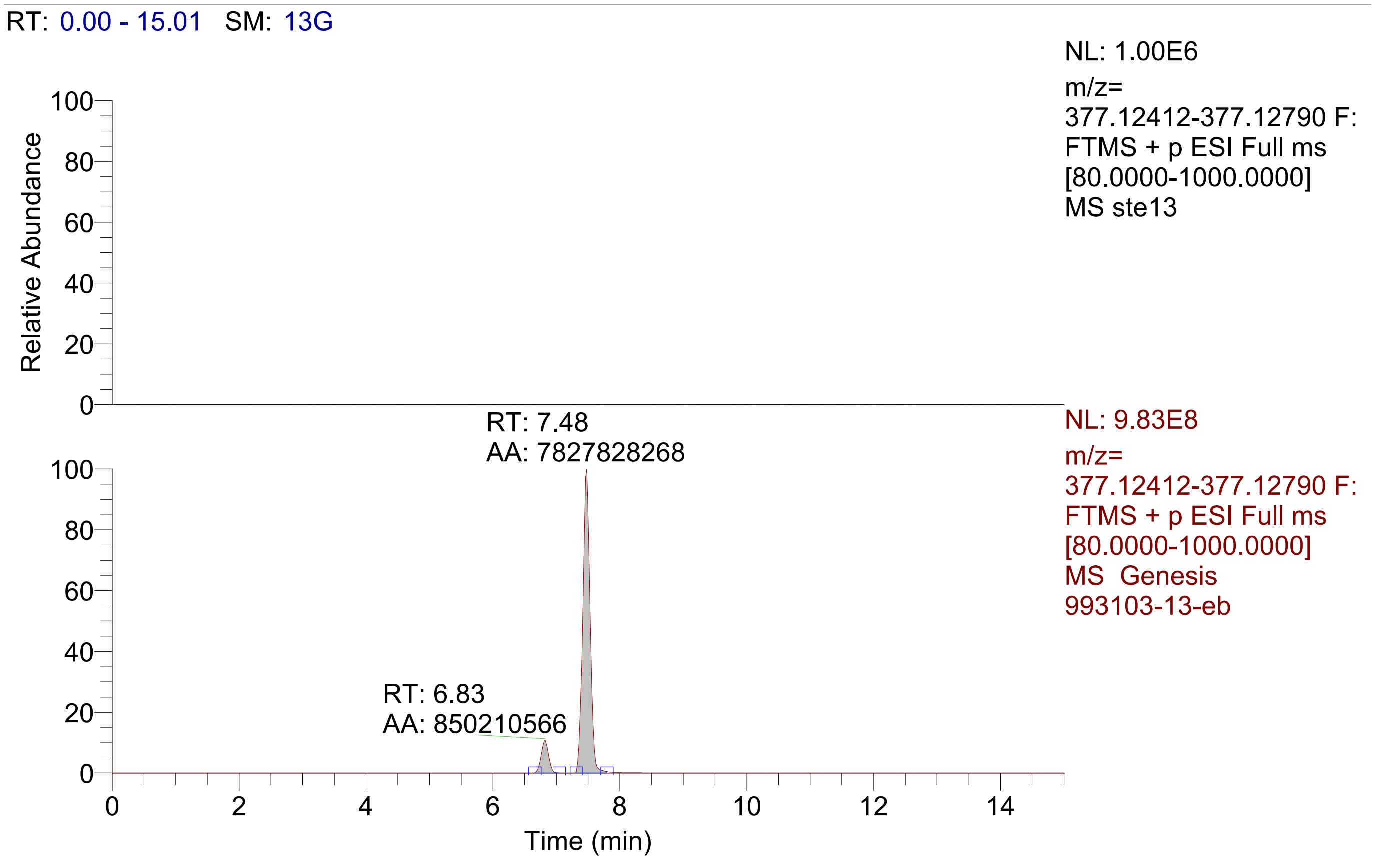

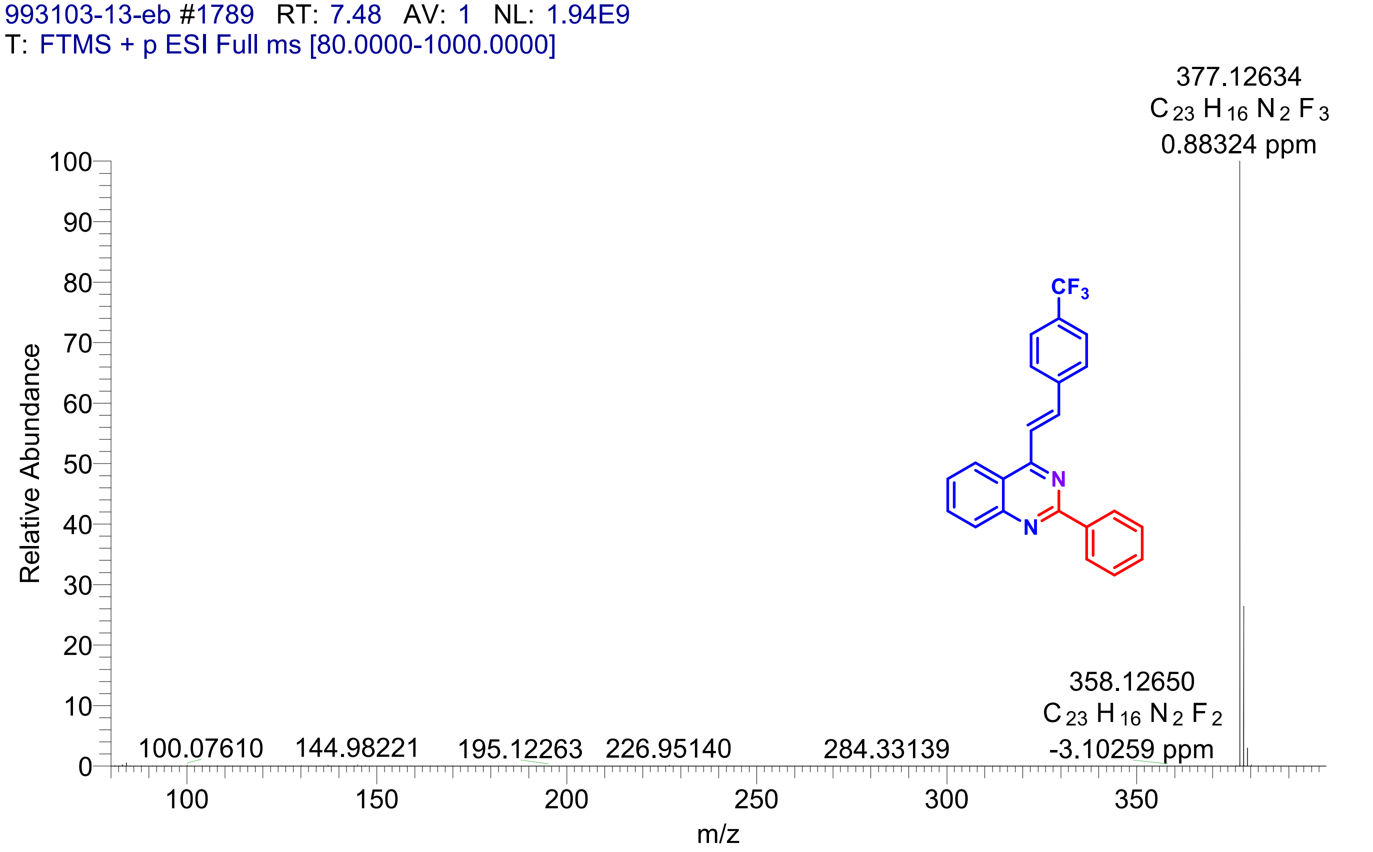

(*E*)–4–(2,3–Dichlorostyryl)–2–phenylquinazoline (3k)

^{1}H NMR spectrum of 3k

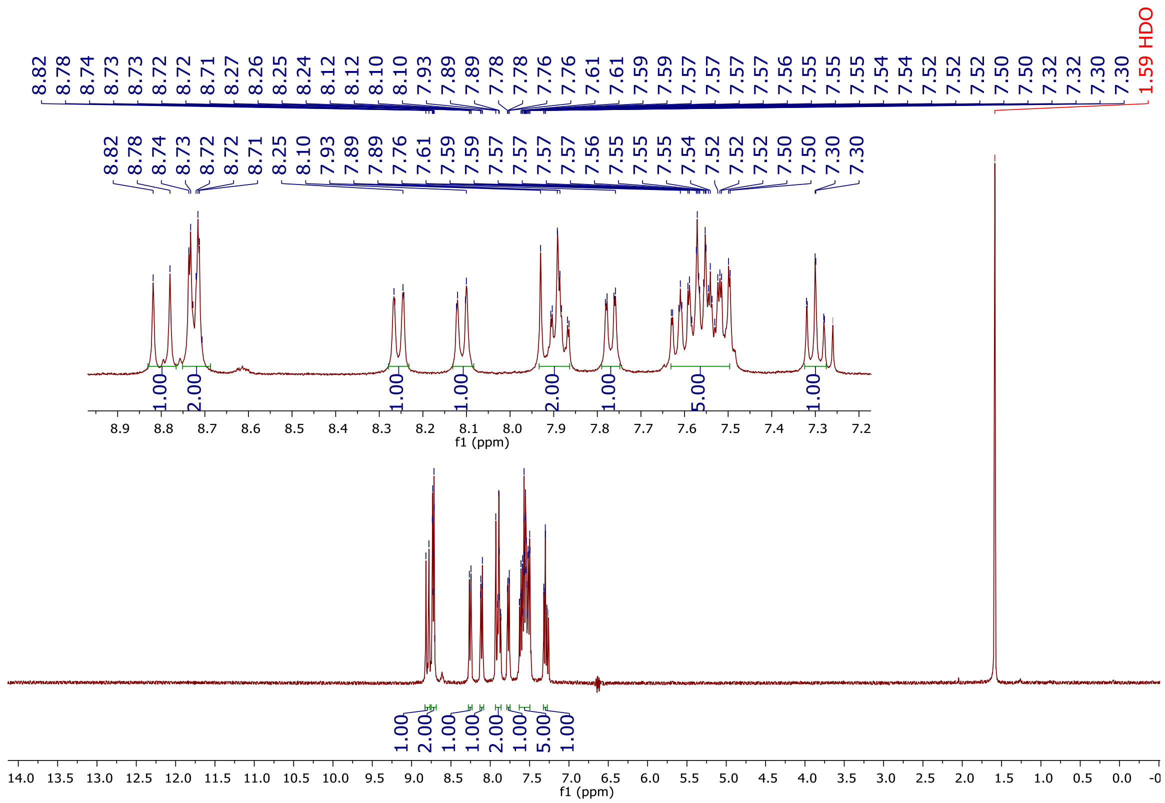

^{13}C NMR spectrum of 3k

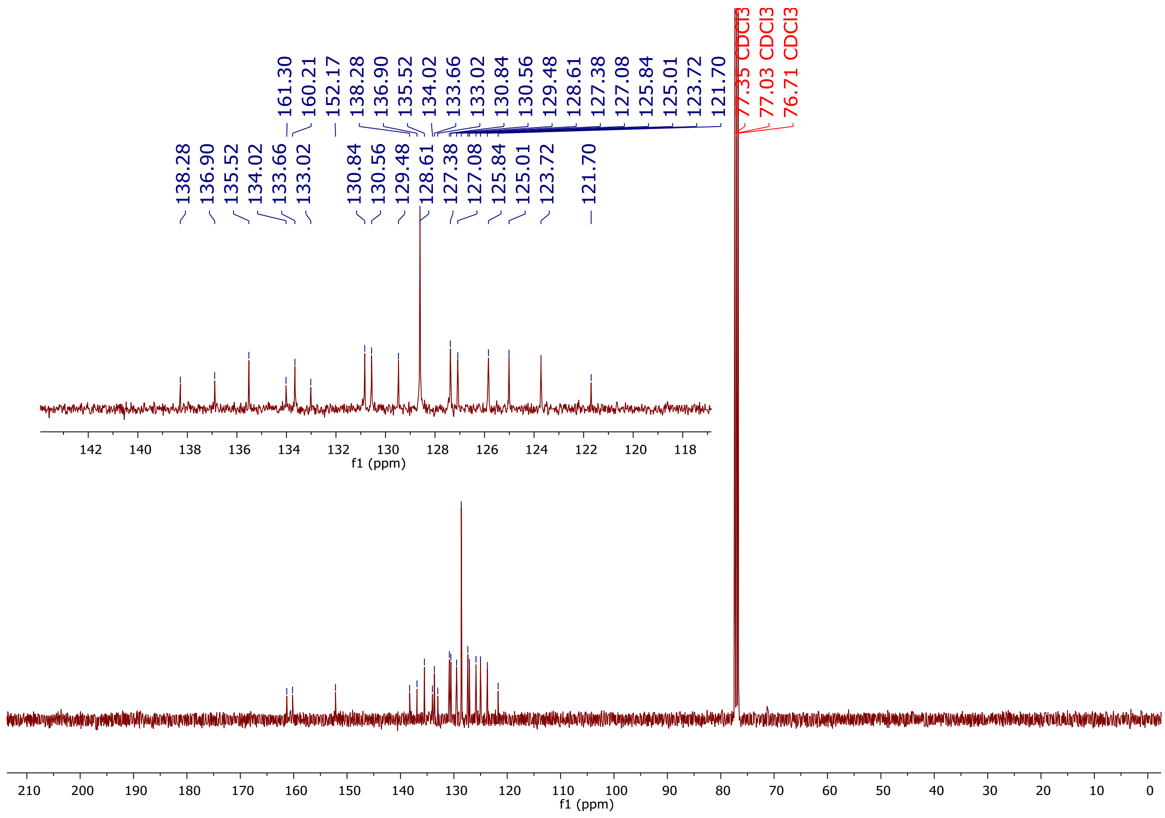

HRMS spectrum of 3k

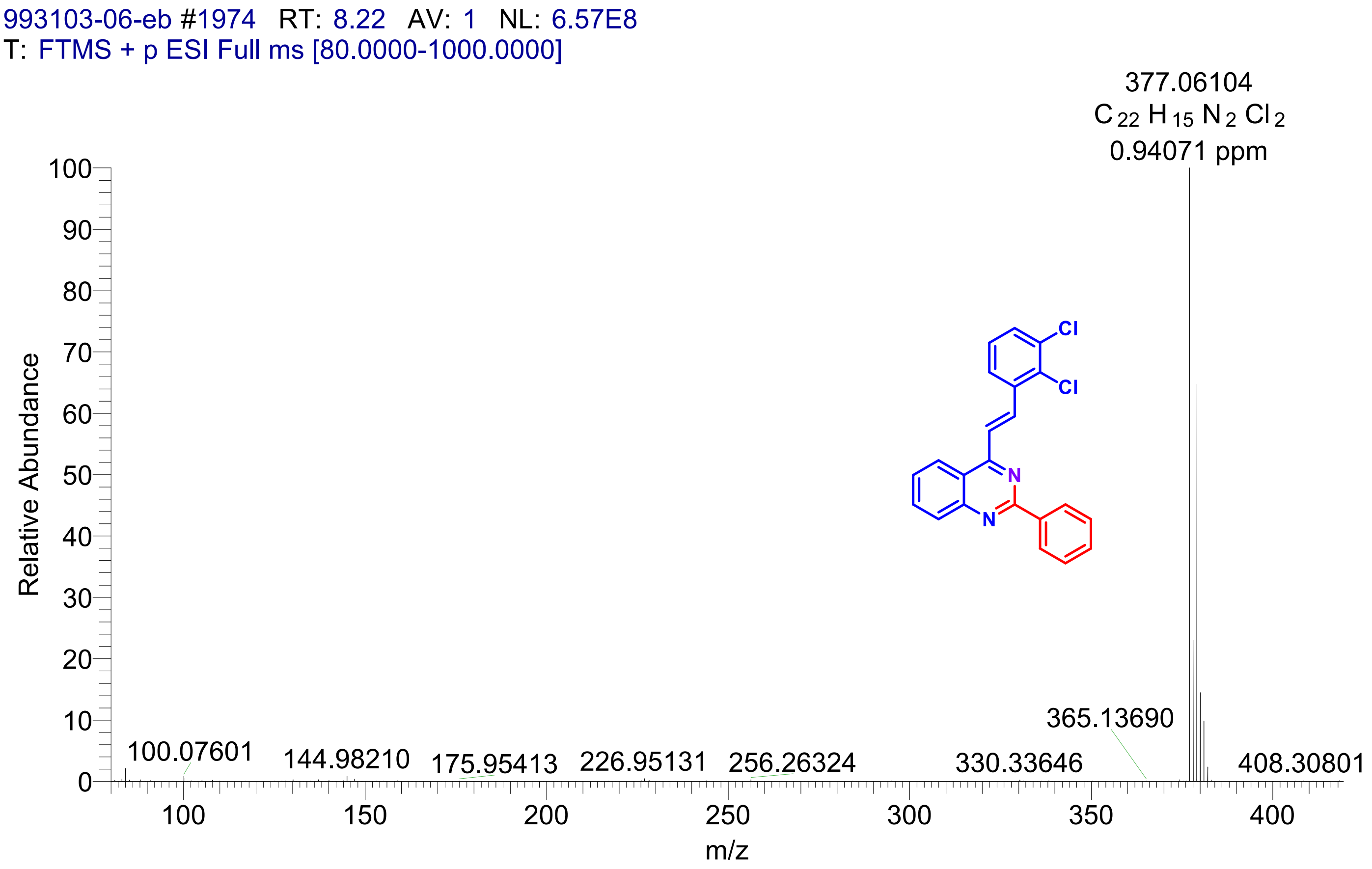

(*E*)–4–(2,4–Dichlorostyryl)–2–phenylquinazoline (3l)

^{1}H NMR spectrum of 3l

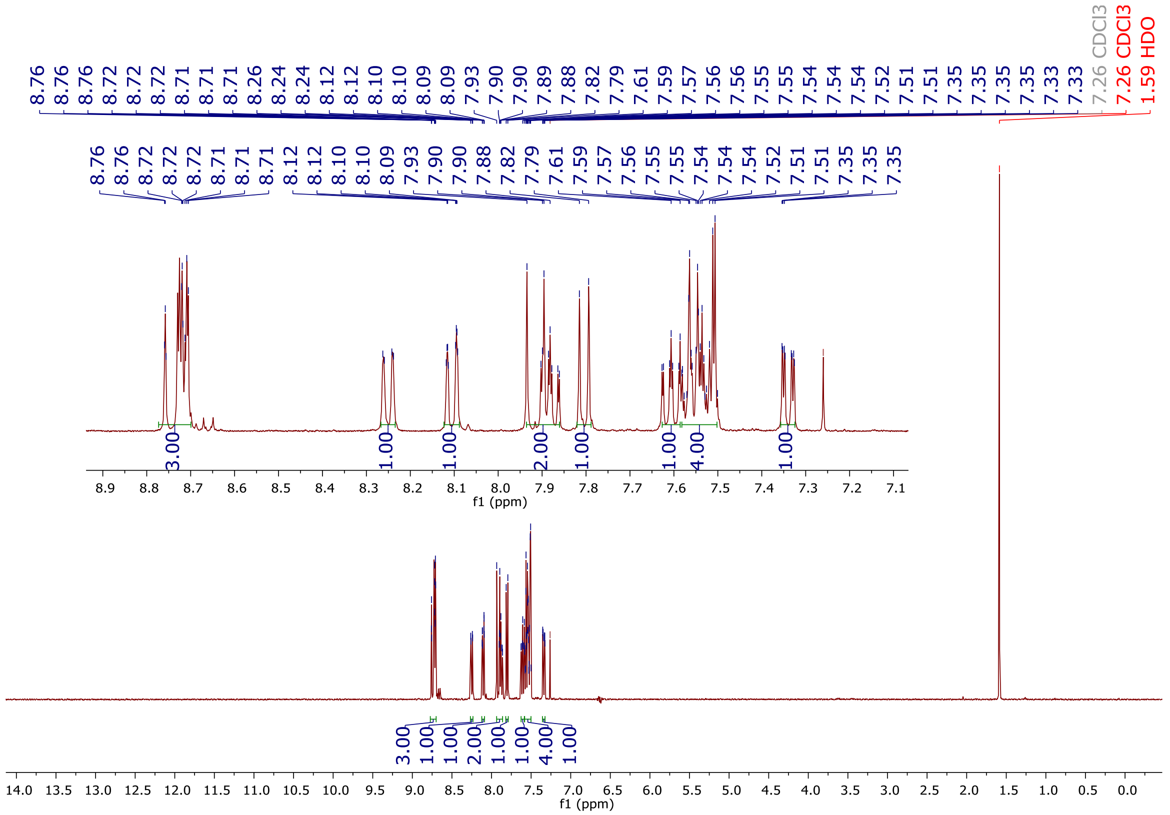

^{1}H NMR spectrum of 3l

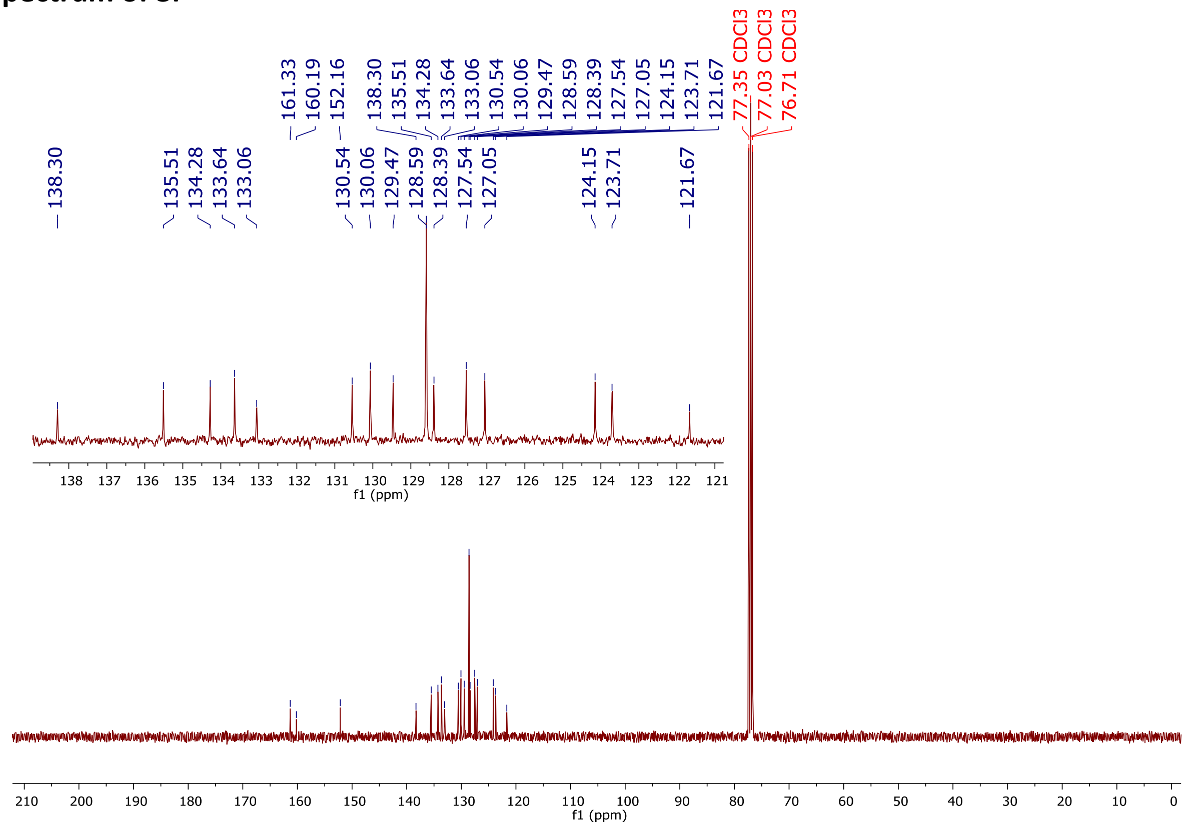

HRMS spectrum of 3l

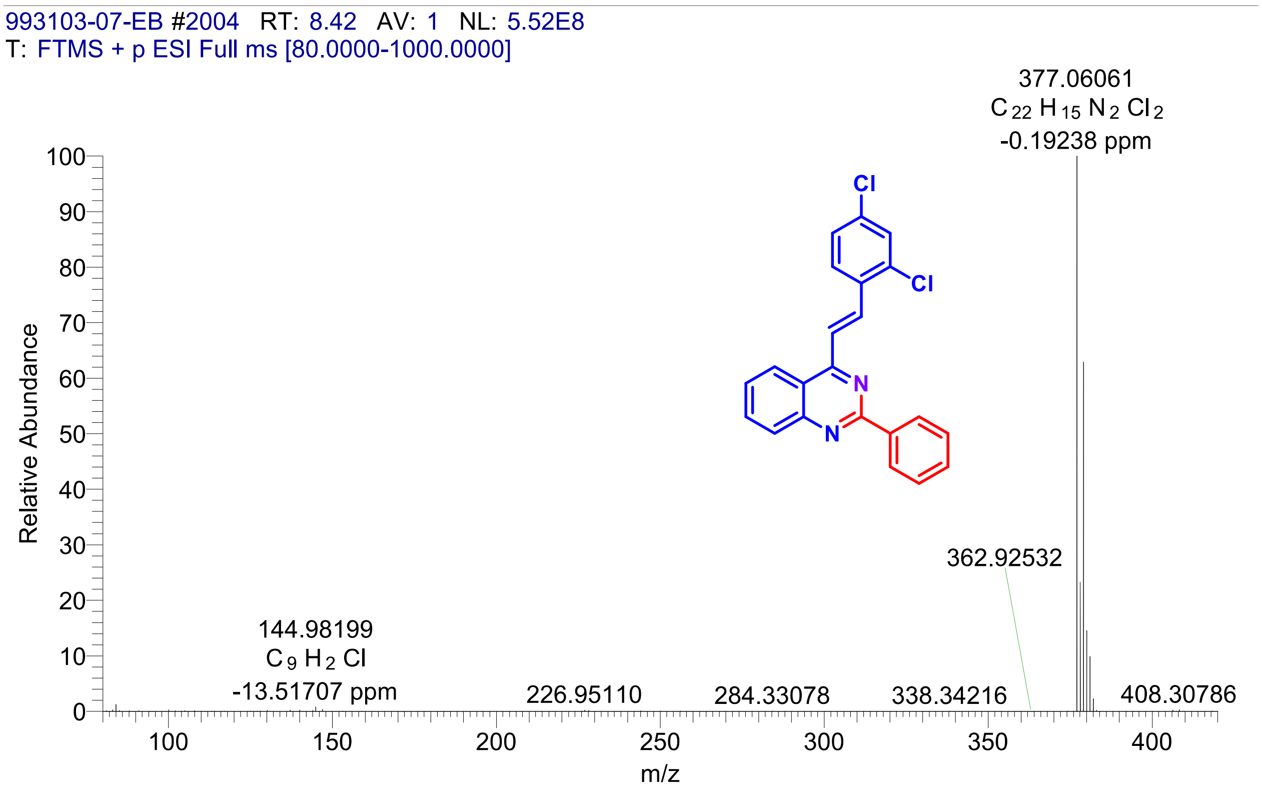

(*E*)–4–(3,4–dichlorostyryl)–2–phenylquinazoline (3m)

^{1}H NMR spectrum of 3m

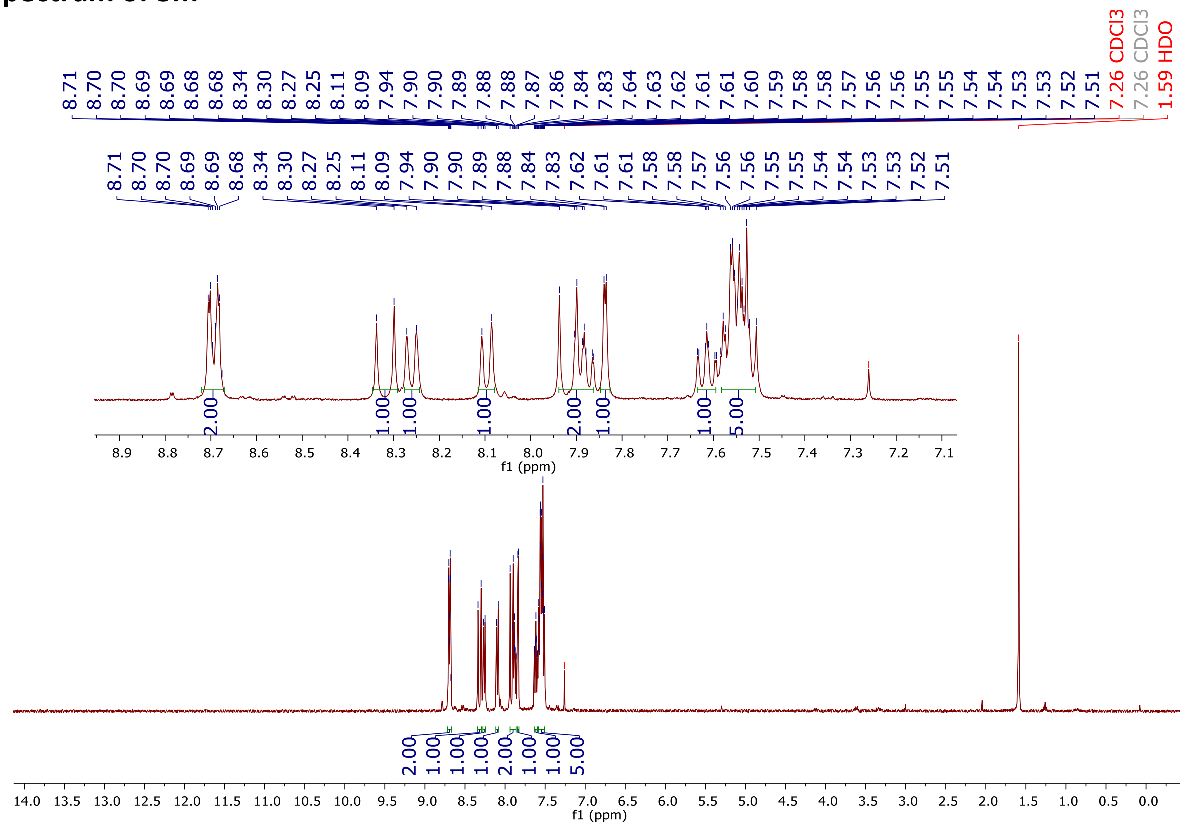

^{13}C NMR spectrum of 3m

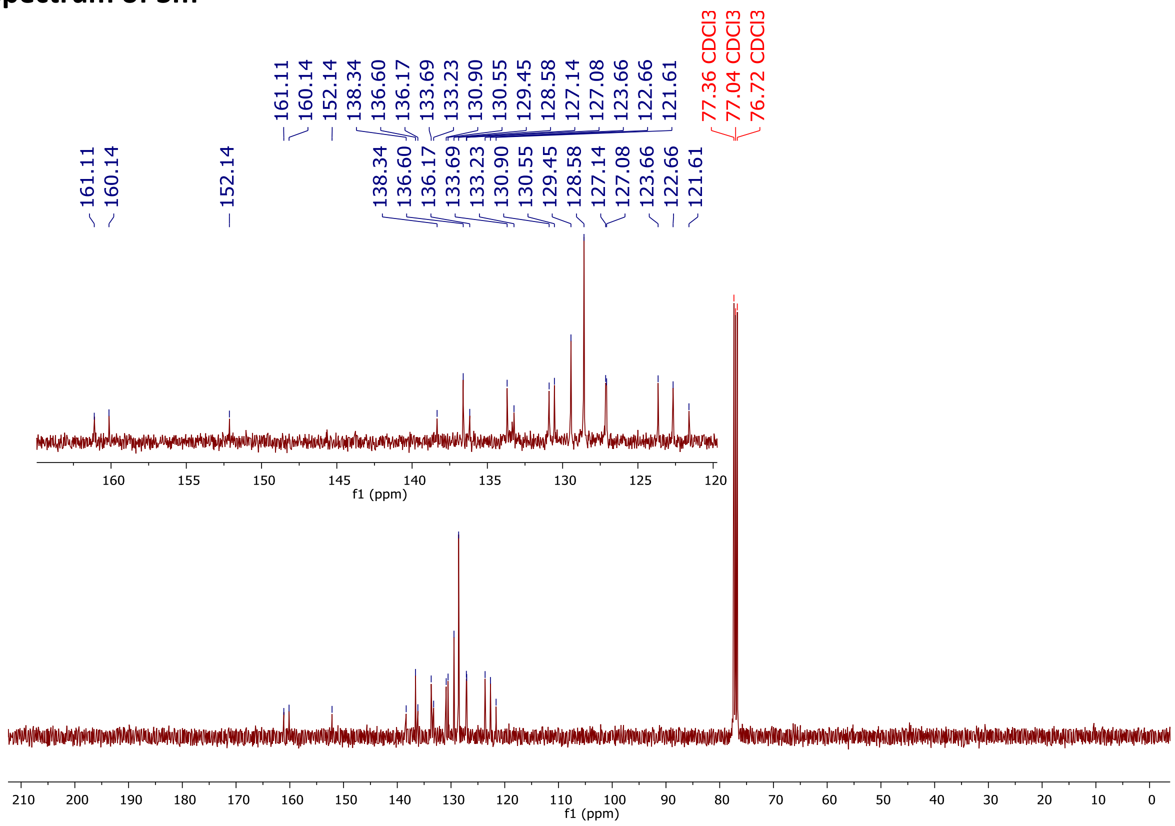

Chromatogram and HRMS spectrum of 3m

Compound Chromatograms

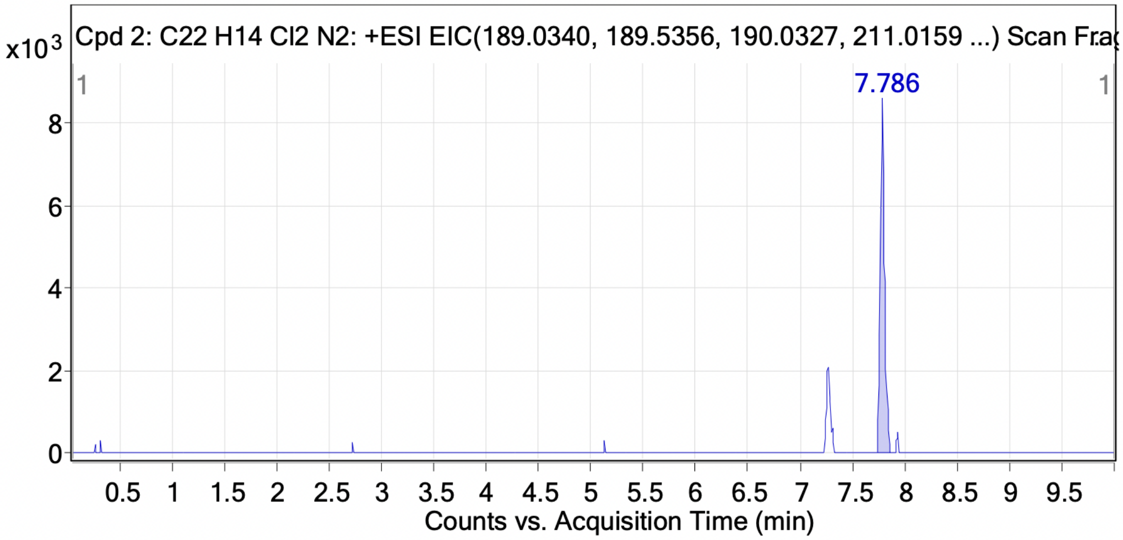

MS Zoomed Spectrum

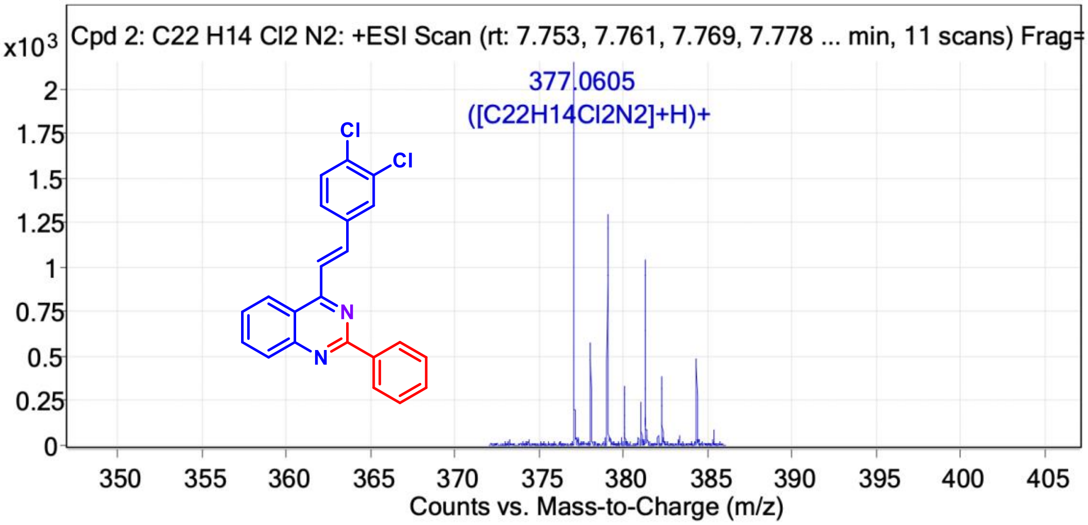

MS Spectrum Peak List

m/z	Calc *m/z*	Diff(ppm)	z	Abund	Formula	Ion
377.0605	377.0607	0.51	1	2246.73	C22H14Cl2N2	(M+H)+
378.0637	378.0639	0.32	1	613.93	C22H14Cl2N2	(M+H)+
379.0583	379.0581	-0.46	1	1376.51	C22H14Cl2N2	(M+H)+
380.0607	380.061	0.79	1	357.97	C22H14Cl2N2	(M+H)+
381.0579	381.0562	-4.34	1	229.4	C22H14Cl2N2	(M+H)+

--- End Of Report ---

(*E*)–4–(2,6–Dichlorostyryl)–2–phenylquinazoline (3n)

^{1}H NMR spectrum of 3n

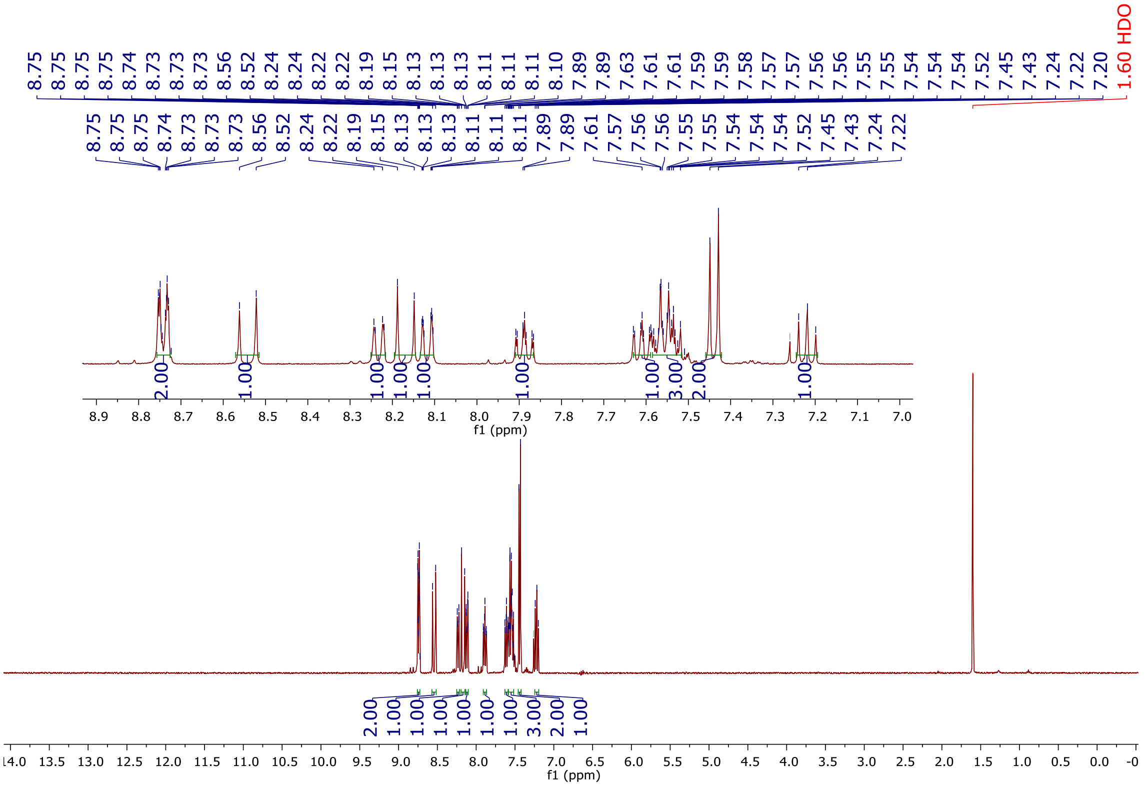

^{13}C NMR spectrum of 3n

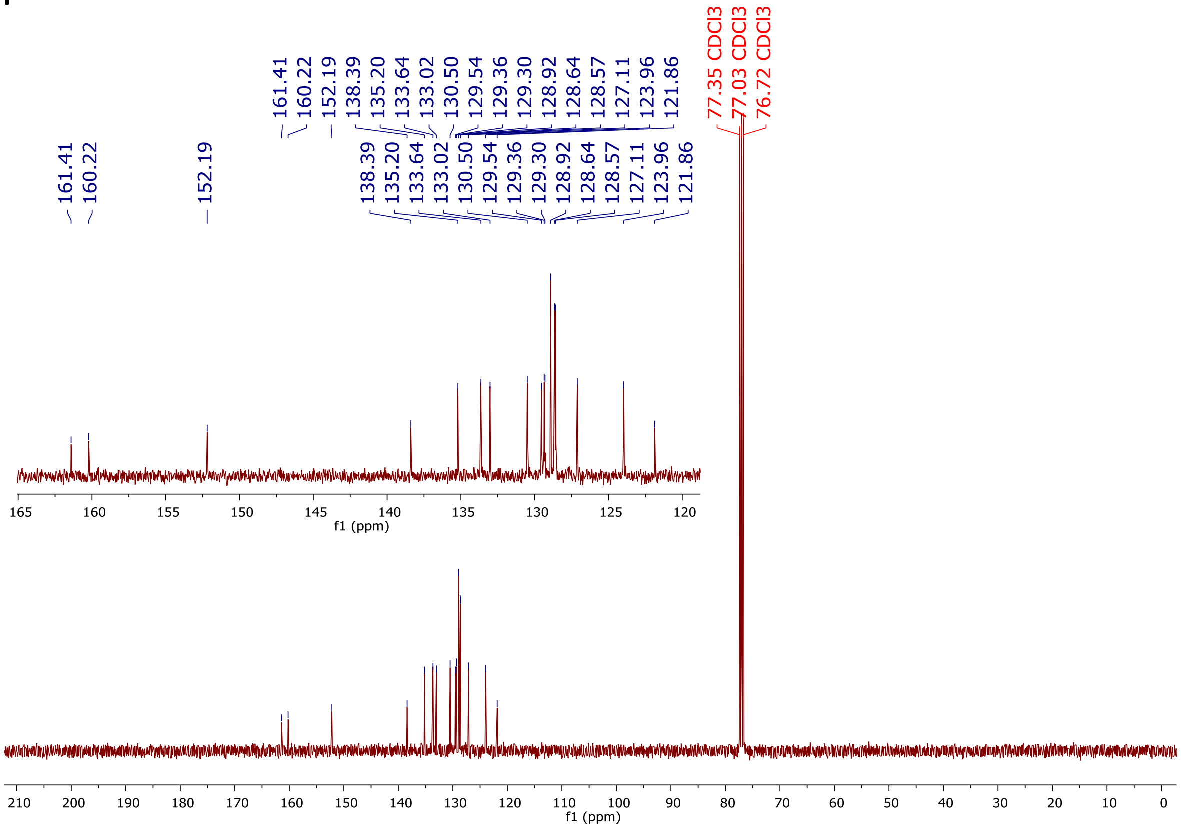

Chromatogram and HRMS spectrum of 3n

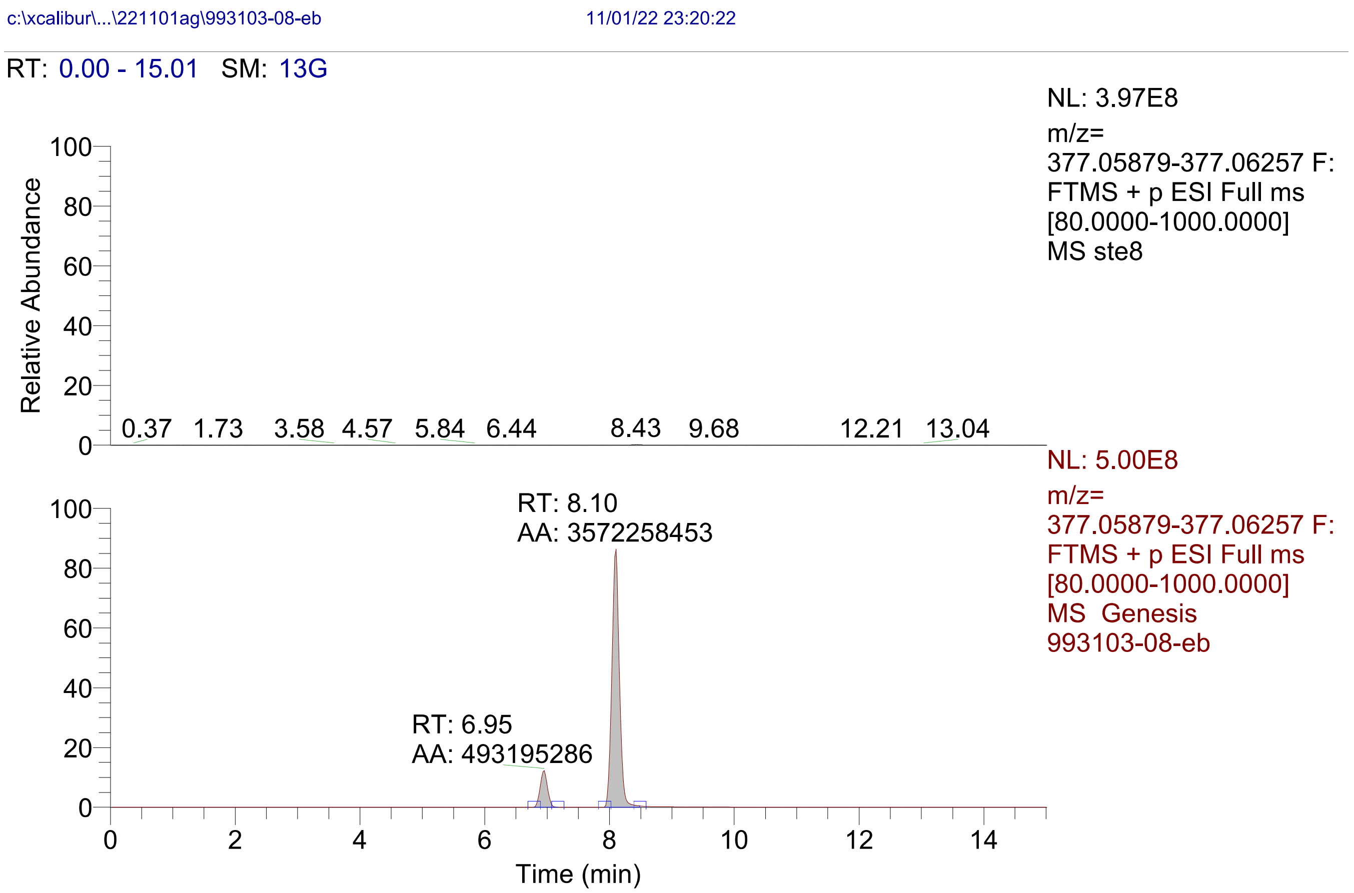

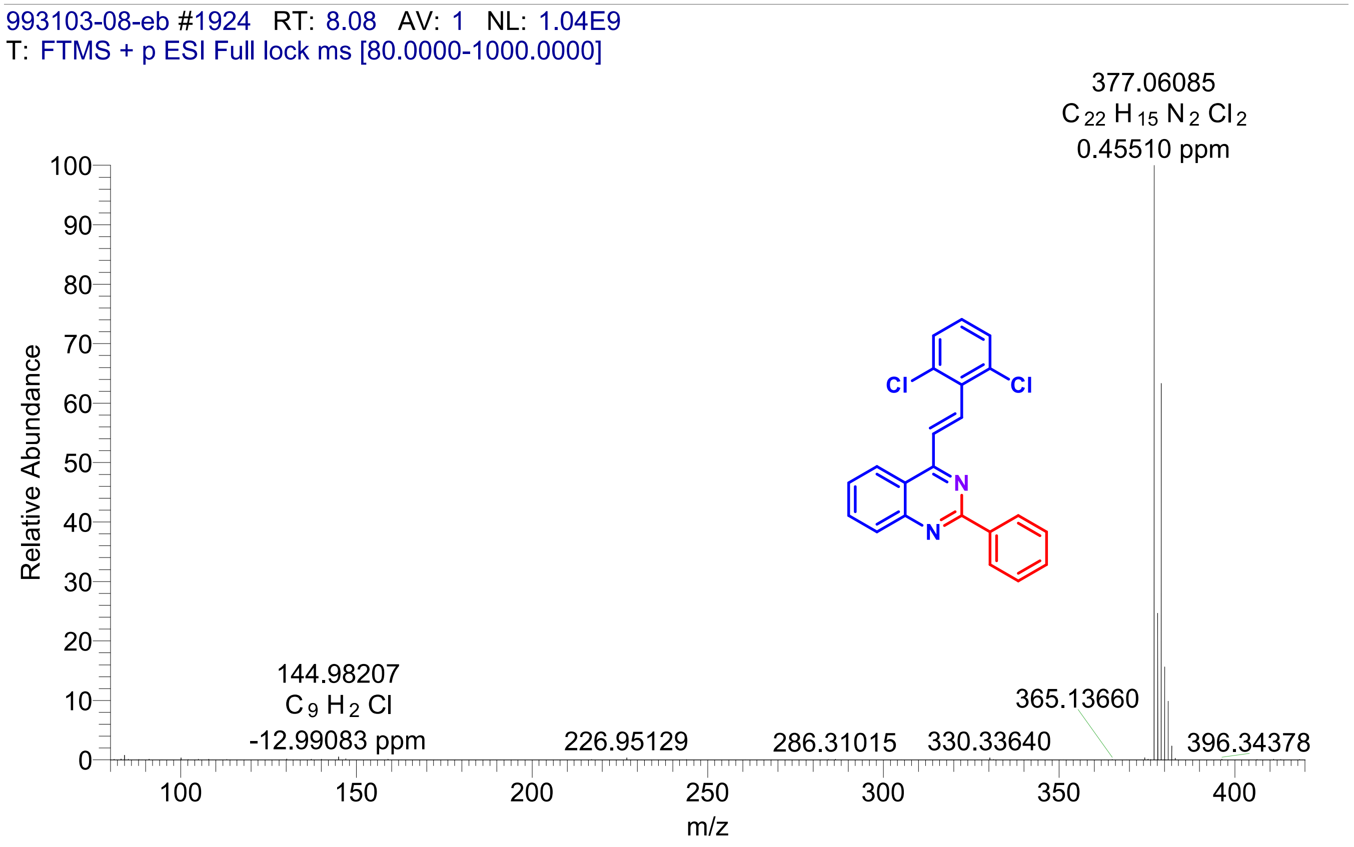

(*E*)–4–(2–Chloro–6–fluorostyryl)–2–phenylquinazoline (3o)

^{1}H NMR spectrum of 3o

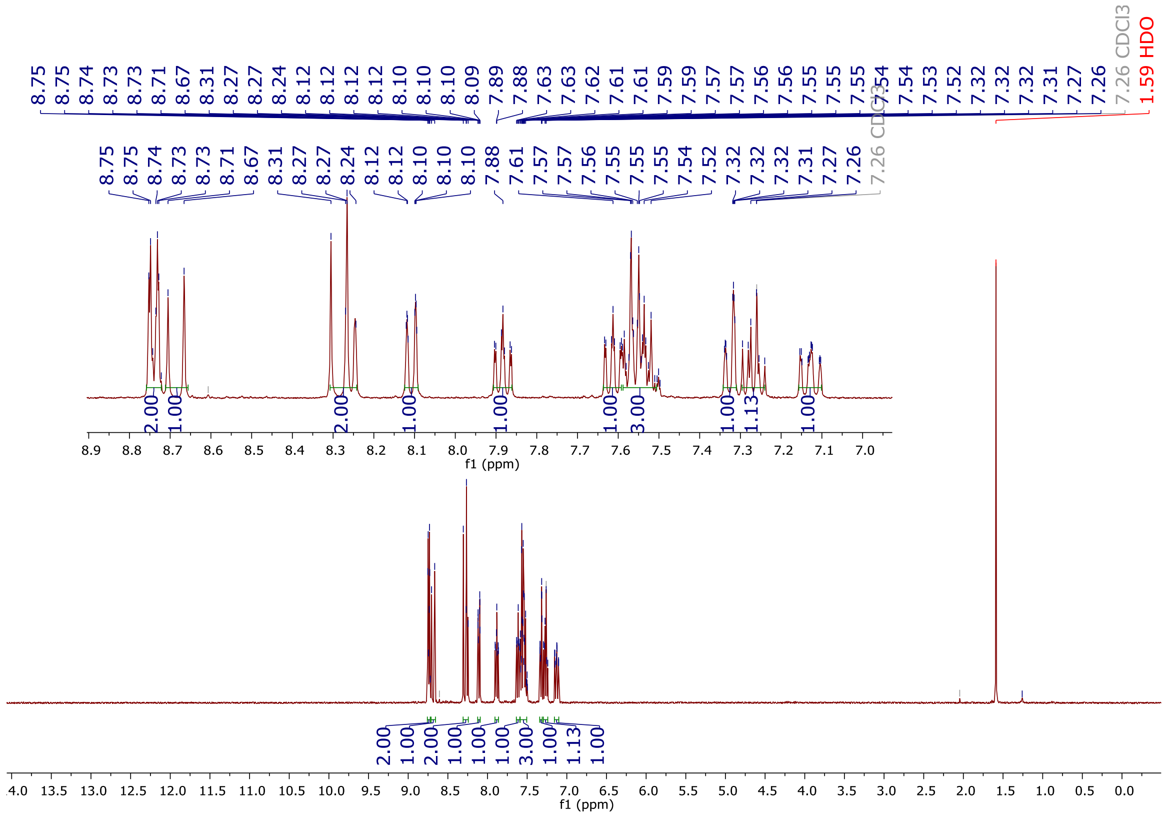

^{13}C NMR spectrum of 3o

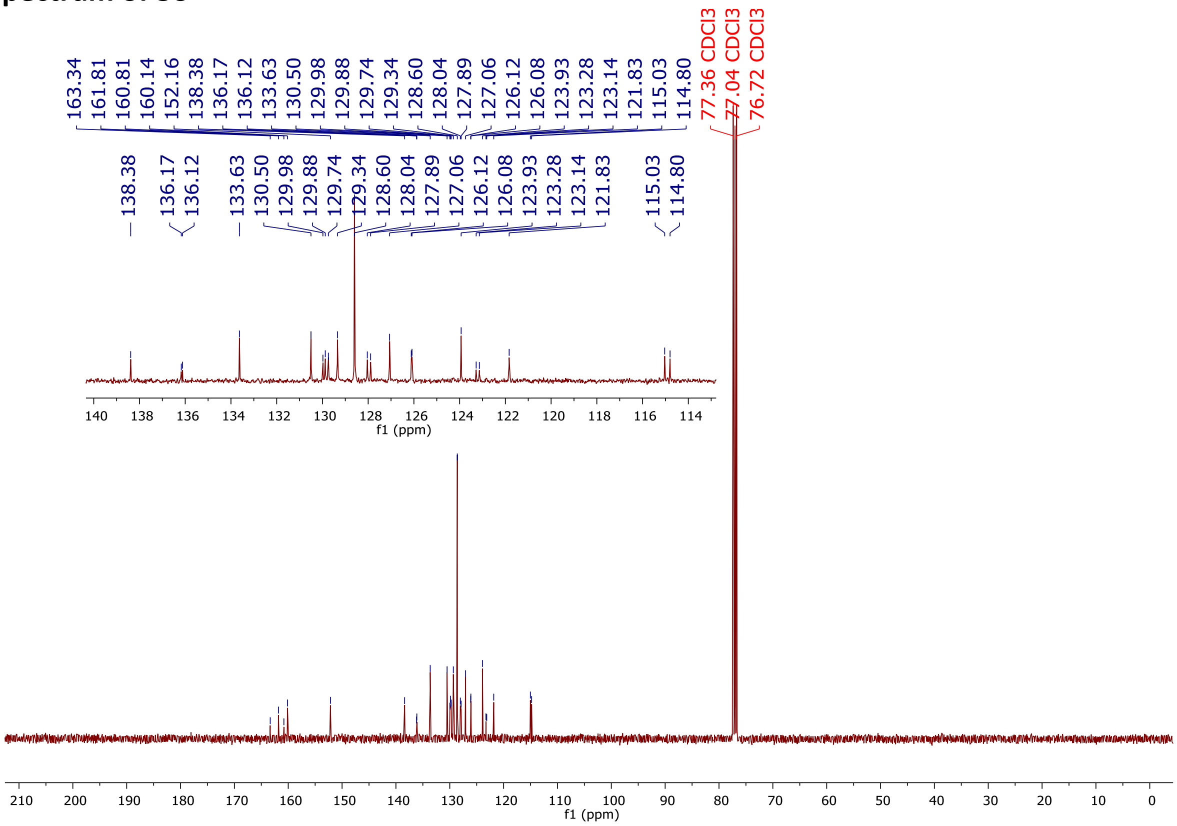

HRMS spectrum of 3o

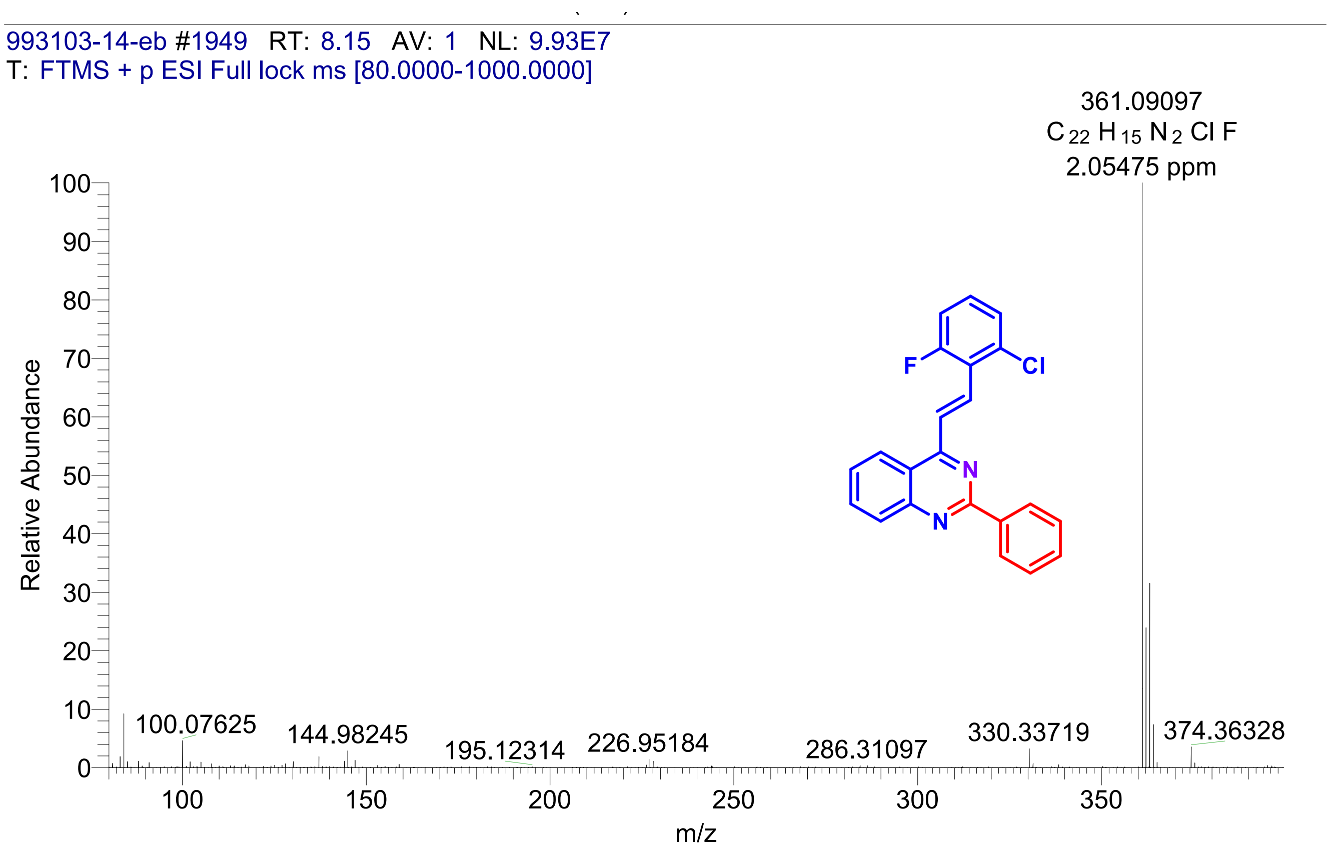

(*E*)–4–(Styryl)quinazoline (5a)

^{1}H NMR spectrum of 5a

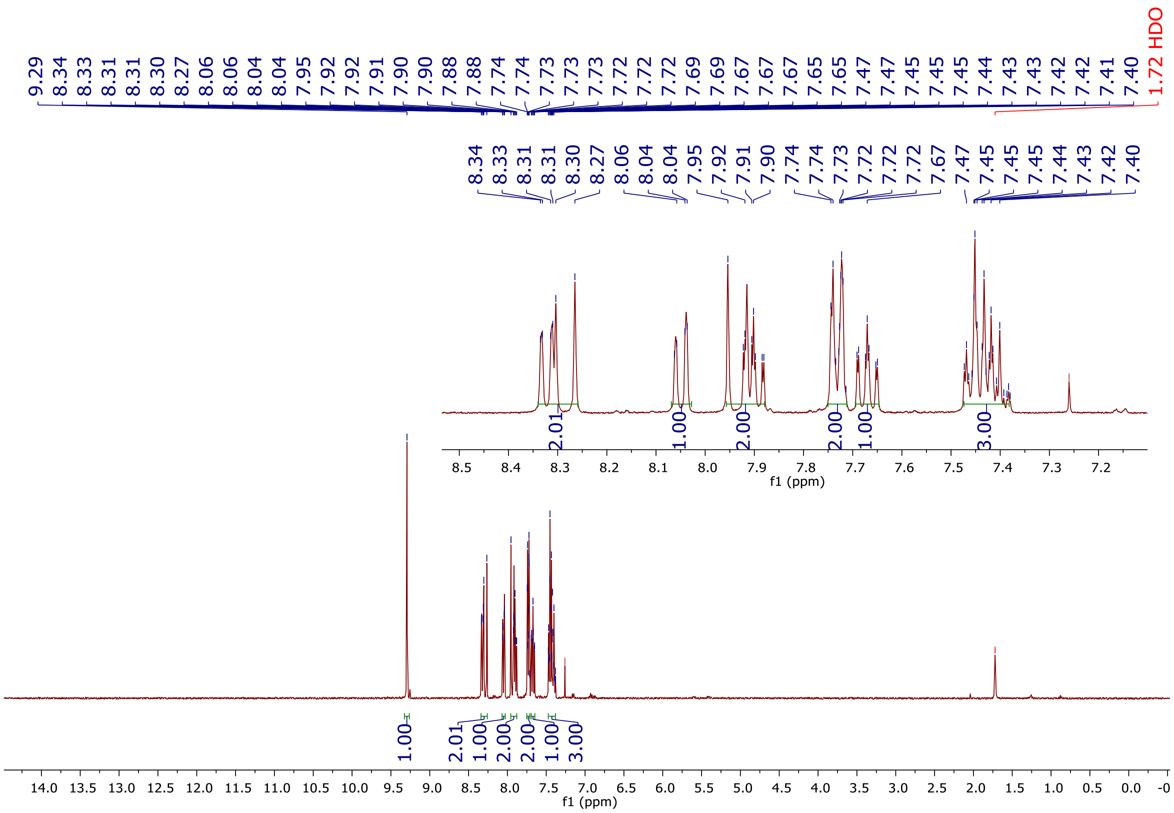

^{13}C NMR spectrum of 5a

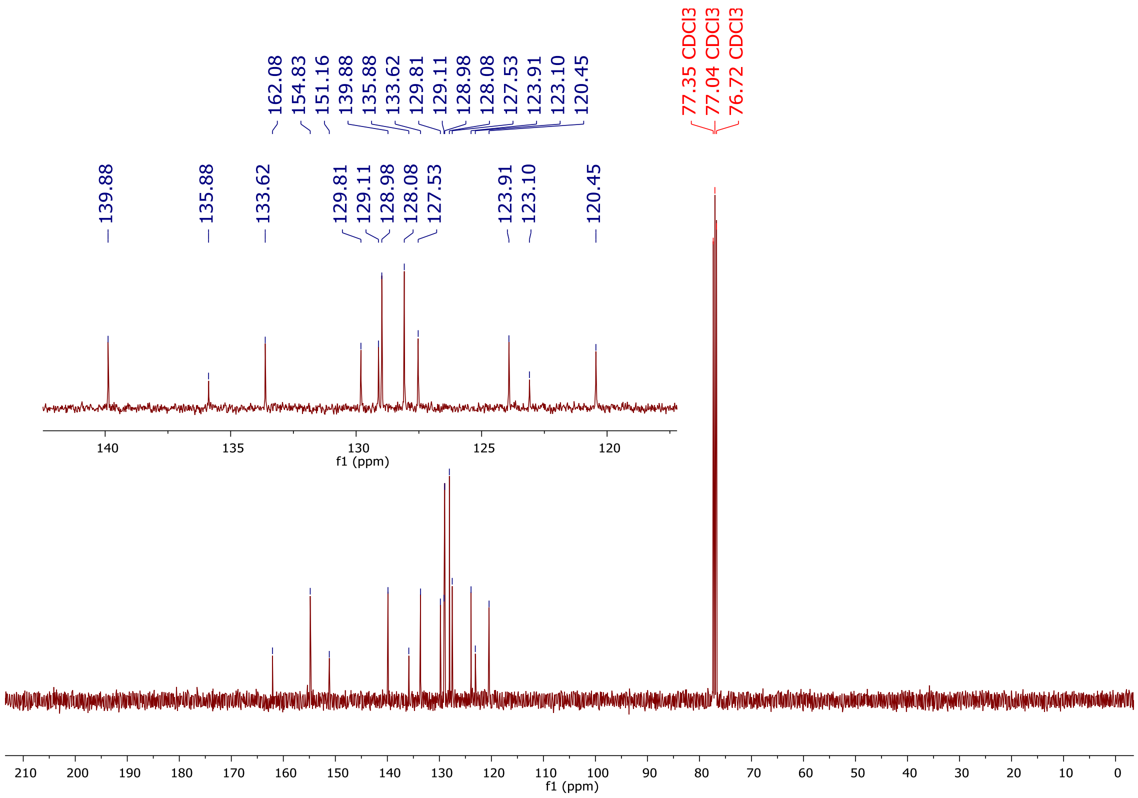

HRMS spectrum of 5a

MS Zoomed Spectrum

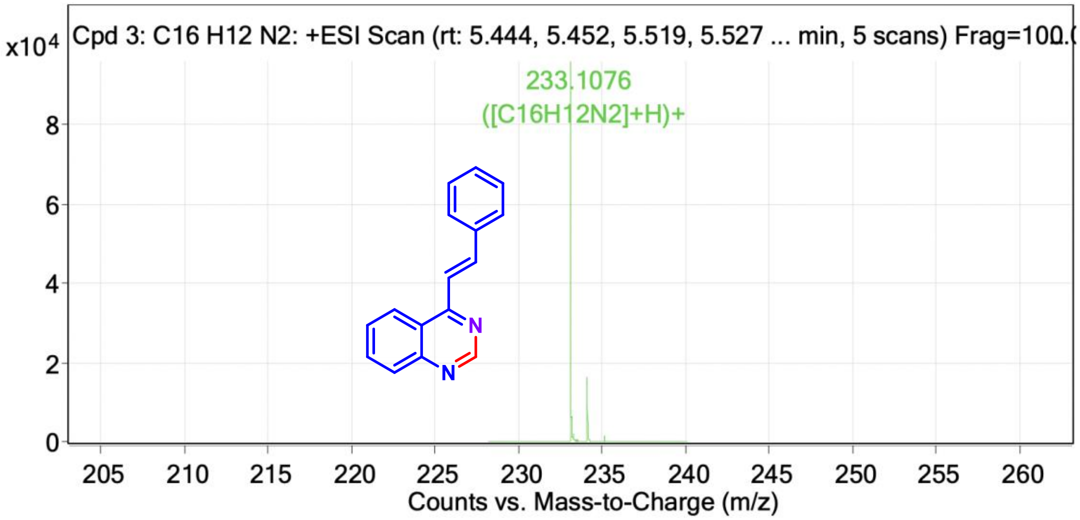

MS Spectrum Peak List

m/z	Calc *m/z*	Diff(ppm)	z	Abund	Formula	Ion
233.1076	233.1073	-1.2	1	99314.86	C16H12N2	(M+H)+
234.1107	234.1104	-1.17	1	16623.69	C16H12N2	(M+H)+
235.1143	235.1136	-3.16	1	1609.85	C16H12N2	(M+H)+

--- End Of Report ---

(*E*)–4–(4–Methoxystyryl)quinazoline (5b)

^{1}H NMR spectrum of 5b

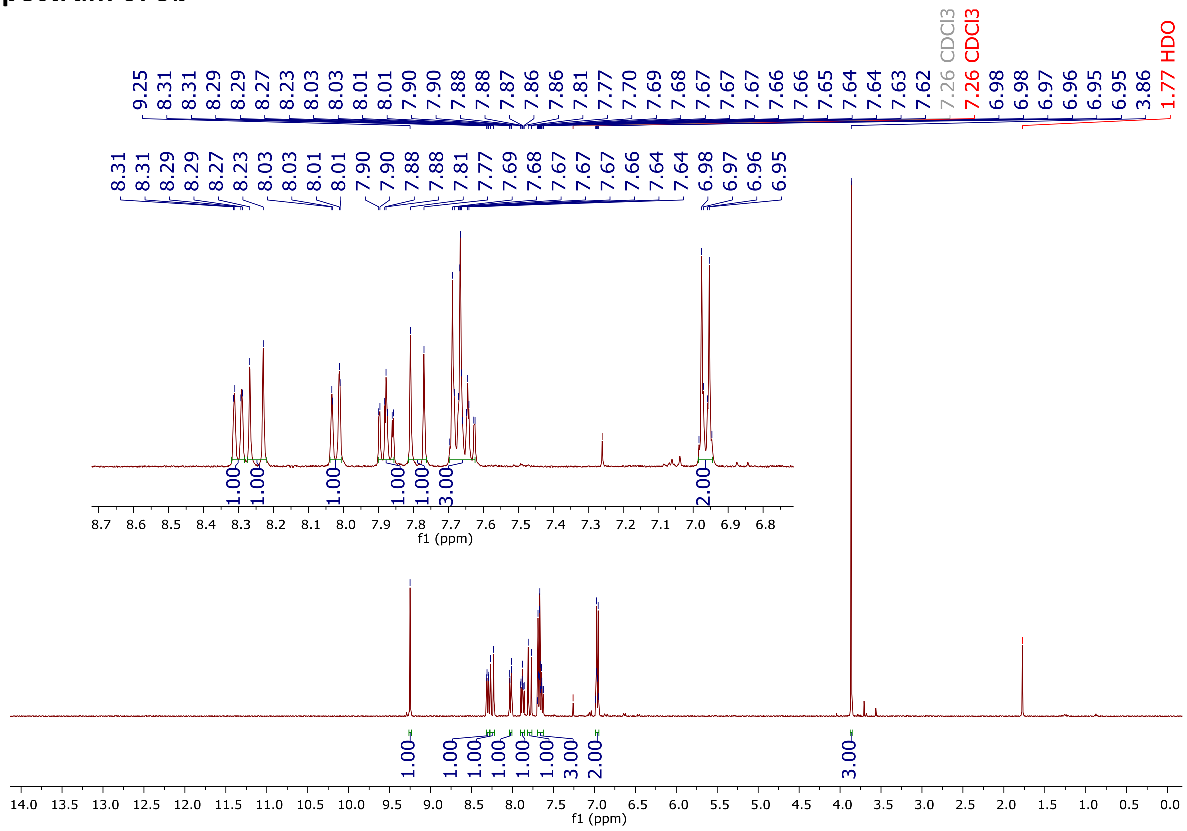

^{13}C NMR spectrum of 5b

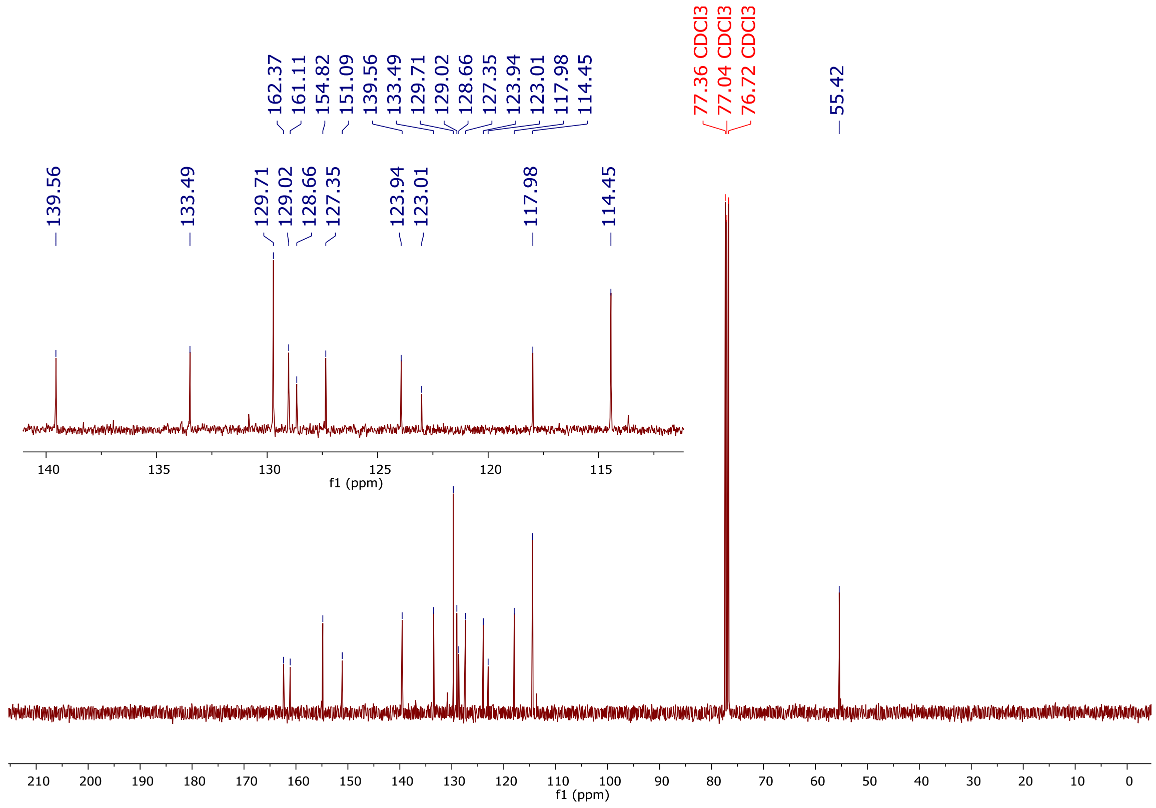

HRMS spectrum of 5b

MS Zoomed Spectrum

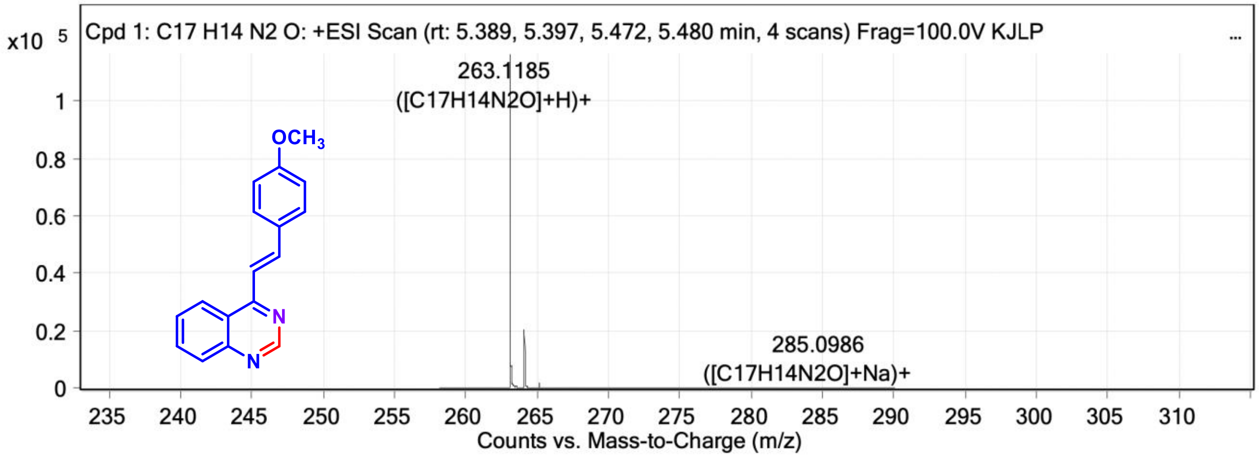

MS Spectrum Peak List

m/z	Calc *m/z*	Diff(ppm)	z	Abund	Formula	Ion
263.1185	263.1179	-2.23	1	116581.25	C17H14N2O	(M+H)+
264.1215	264.121	-1.8	1	21084.46	C17H14N2O	(M+H)+
265.1246	265.124	-2.43	1	2069.32	C17H14N2O	(M+H)+
285.0986	285.0998	4.5	1	63.75	C17H14N2O	(M+Na)+

--- End Of Report ---

(*E*)–4–(4–Bromostyryl)quinazoline (5c)

^{1}H NMR spectrum of 5c

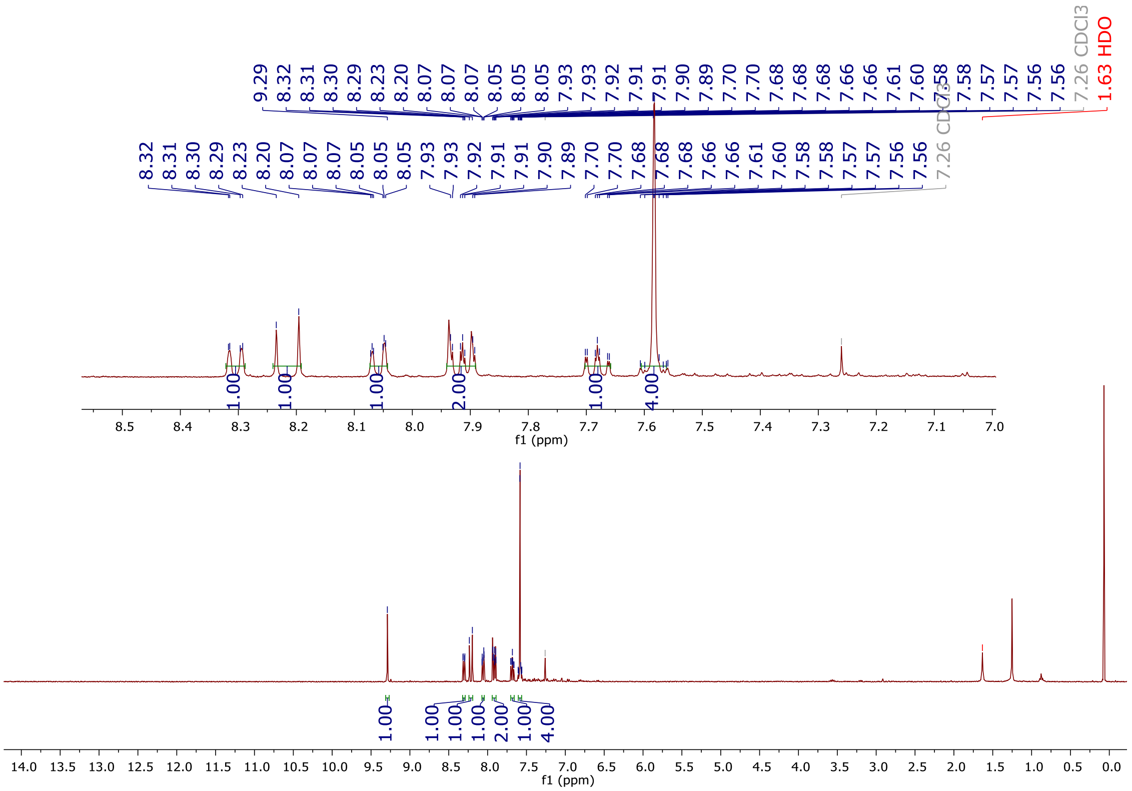

^{13}C NMR spectrum of 5c

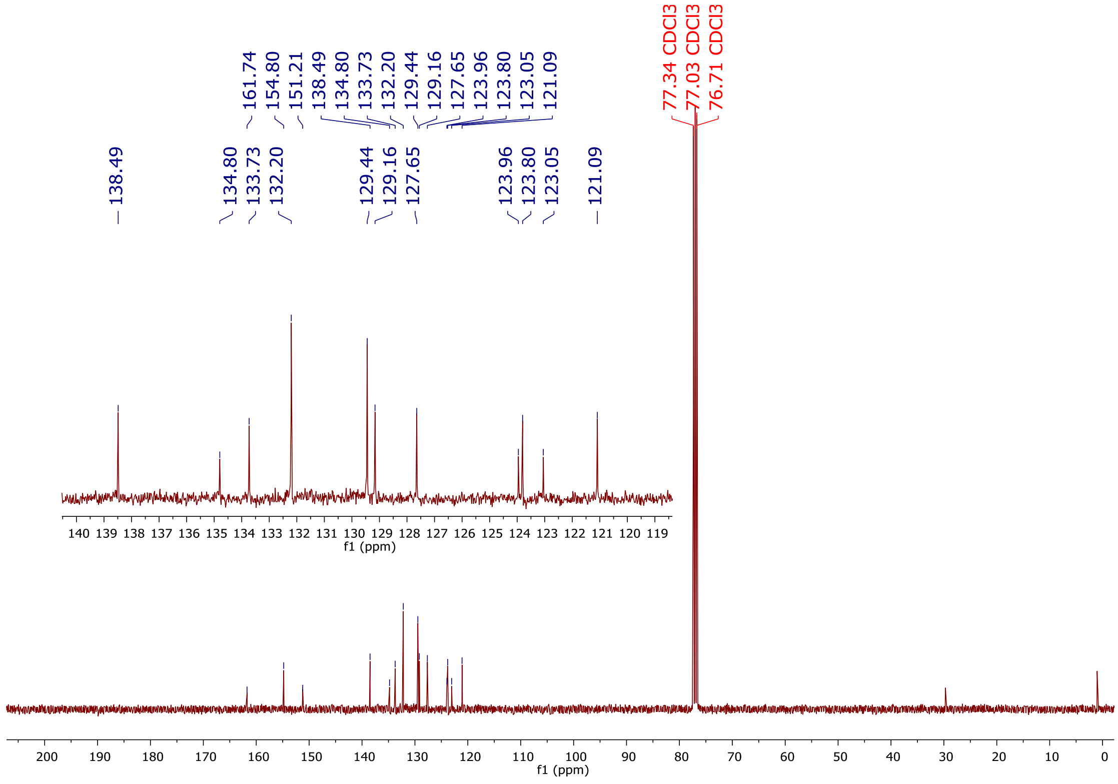

HRMS spectrum of 3c

MS Zoomed Spectrum

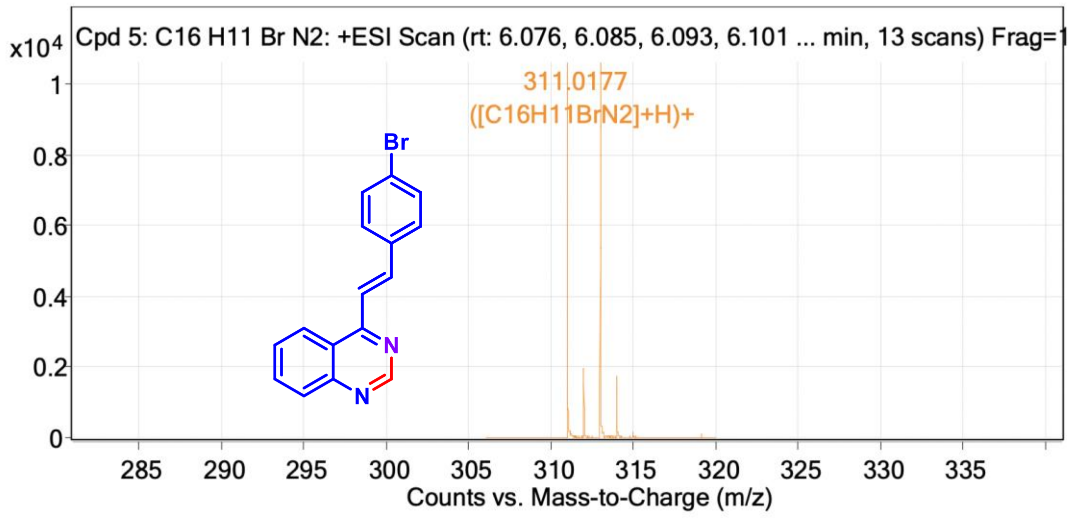

MS Spectrum Peak List

m/z	Calc *m/z*	Diff(ppm)	z	Abund	Formula	Ion
311.0177	311.0178	0.51	1	10909.16	C16H11BrN2	(M+H)+
312.0204	312.021	1.76	1	2062.76	C16H11BrN2	(M+H)+
313.0157	313.0159	0.55	1	10670.34	C16H11BrN2	(M+H)+
314.0189	314.019	0.13	1	1836.99	C16H11BrN2	(M+H)+
315.0214	315.022	2.14	1	126.85	C16H11BrN2	(M+H)+

--- End Of Report ---

(*E*)–4–(4–Chlorostyryl)quinazoline (5d)

^{1}H NMR spectrum of 5d

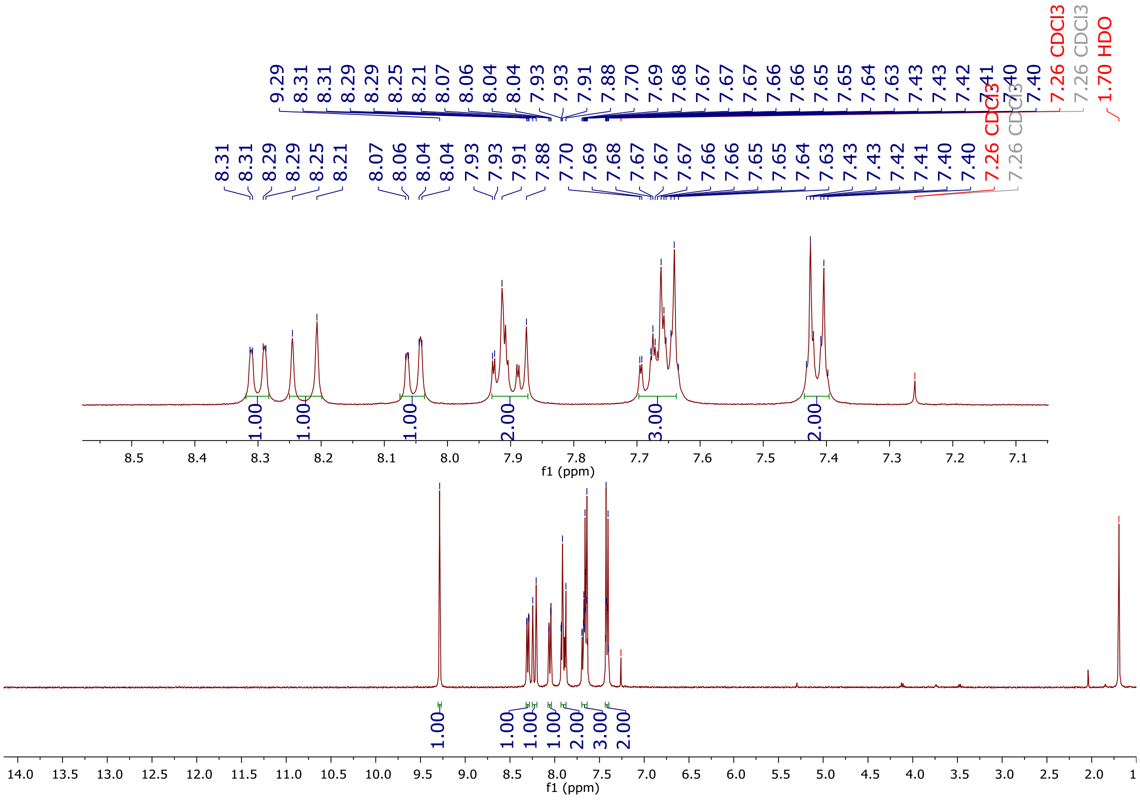

^{13}C NMR spectrum of 5d

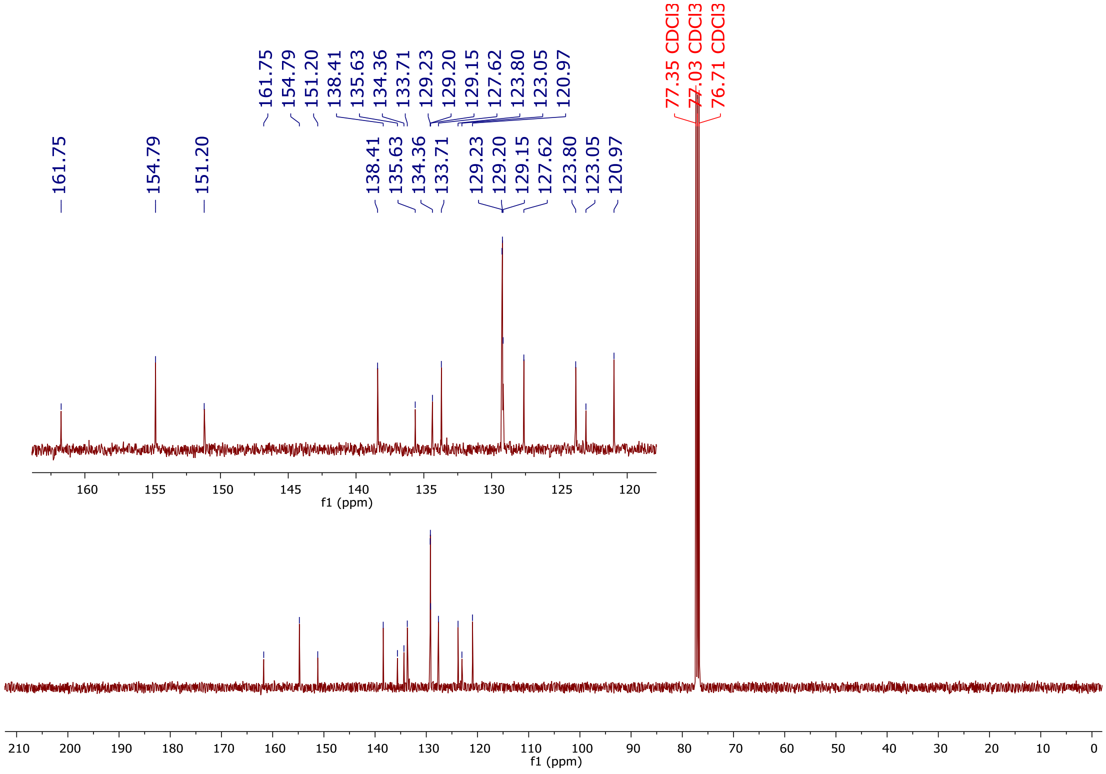

HRMS spectrum of 5d

MS Zoomed Spectrum

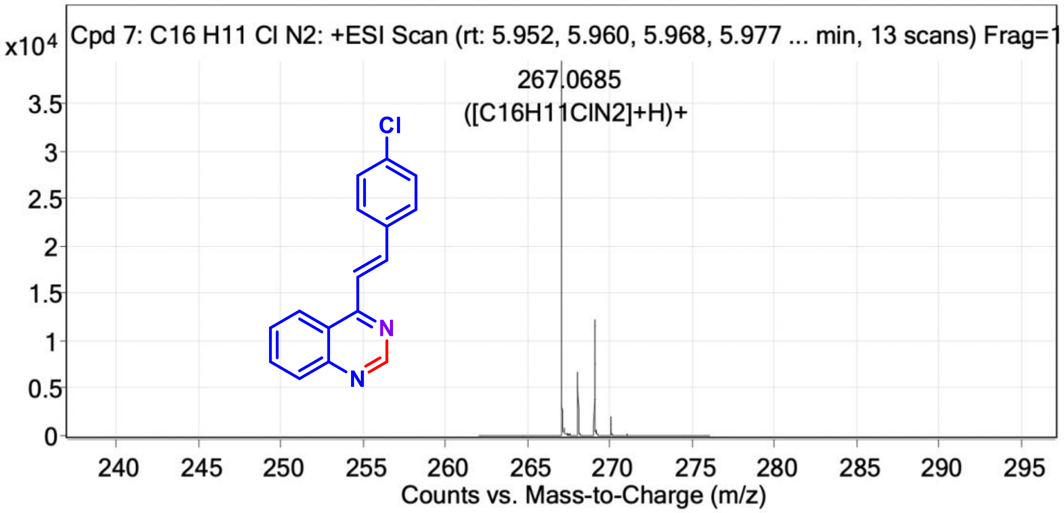

MS Spectrum Peak List

m/z	Calc *m/z*	Diff(ppm)	z	Abund	Formula	Ion
267.0685	267.0684	-0.49	1	39671.45	C16H11ClN2	(M+H)+
268.0718	268.0715	-1.37	1	6808.85	C16H11ClN2	(M+H)+
269.0659	269.0658	-0.22	1	12614.09	C16H11ClN2	(M+H)+
270.0683	270.0687	1.31	1	2187.28	C16H11ClN2	(M+H)+
271.0738	271.0717	-7.87	1	155.37	C16H11ClN2	(M+H)+

--- End Of Report ---

(*E*)–4–(4–Fluorostyryl)quinazoline (5e)

^{1}H NMR spectrum of 5e

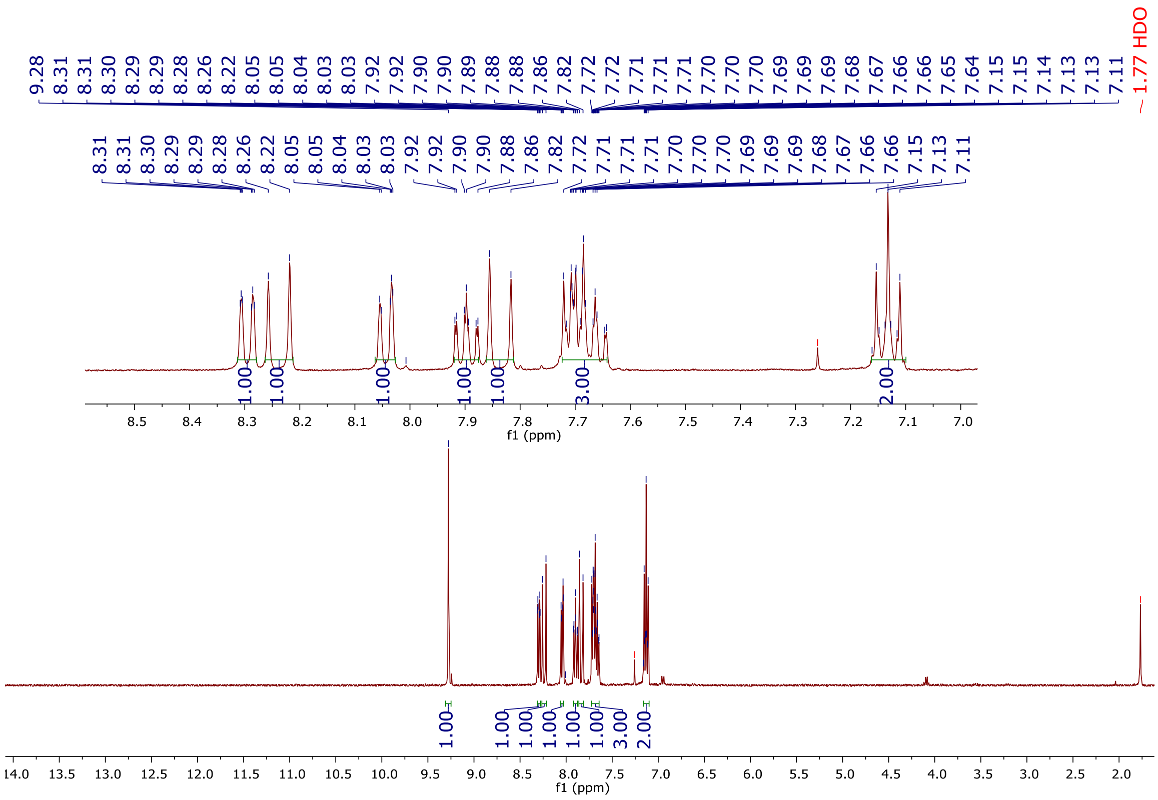

^{13}C NMR spectrum of 5e

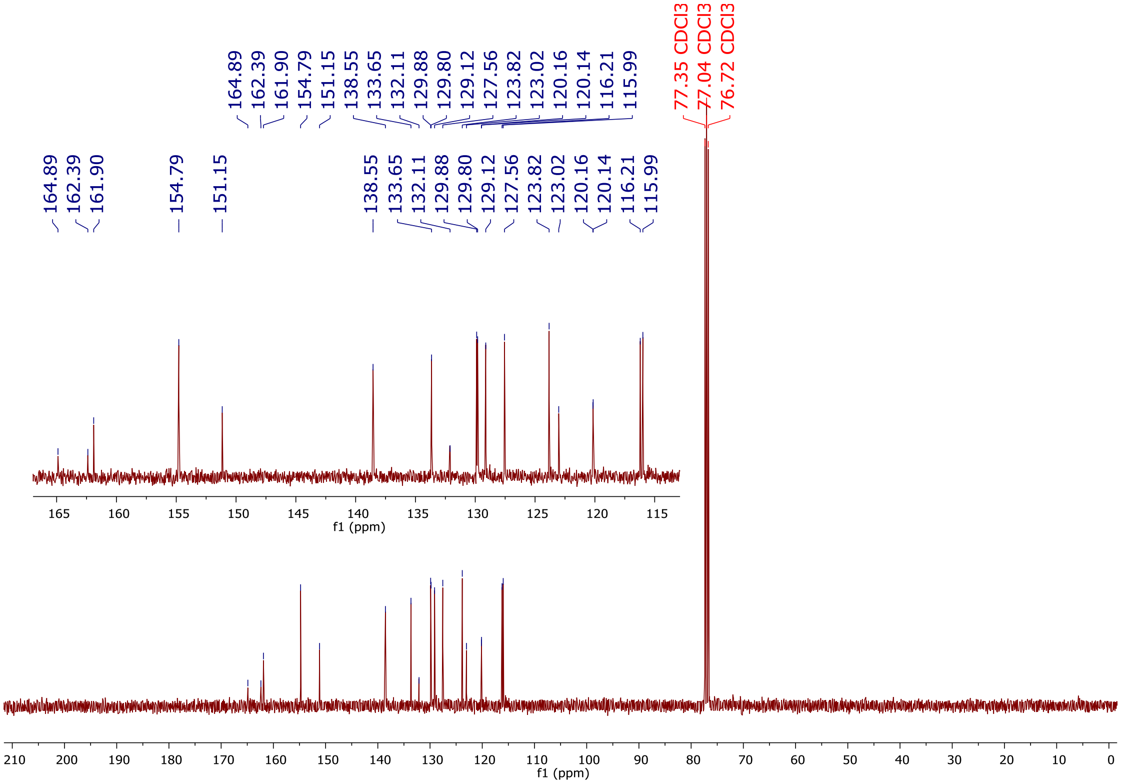

HRMS spectrum of 5e

MS Zoomed Spectrum

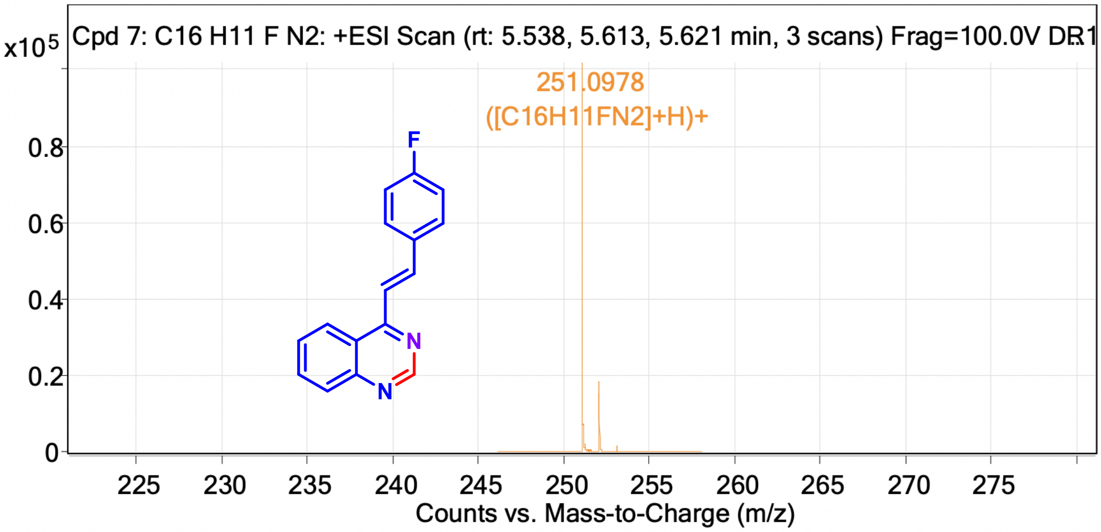

MS Spectrum Peak List

m/z	Calc *m/z*	Diff(ppm)	z	Abund	Formula	Ion
251.0978	251.0979	0.51	1	106000.8	C16H11FN2	(M+H)+
252.1009	252.101	0.64	1	18572.72	C16H11FN2	(M+H)+
253.1053	253.1041	-4.73	1	1742.95	C16H11FN2	(M+H)+

--- End Of Report ---

(*E*)–4–(4–(Trifluoromethyl)styryl)quinazoline (5f)

^{1}H NMR spectrum of 5f

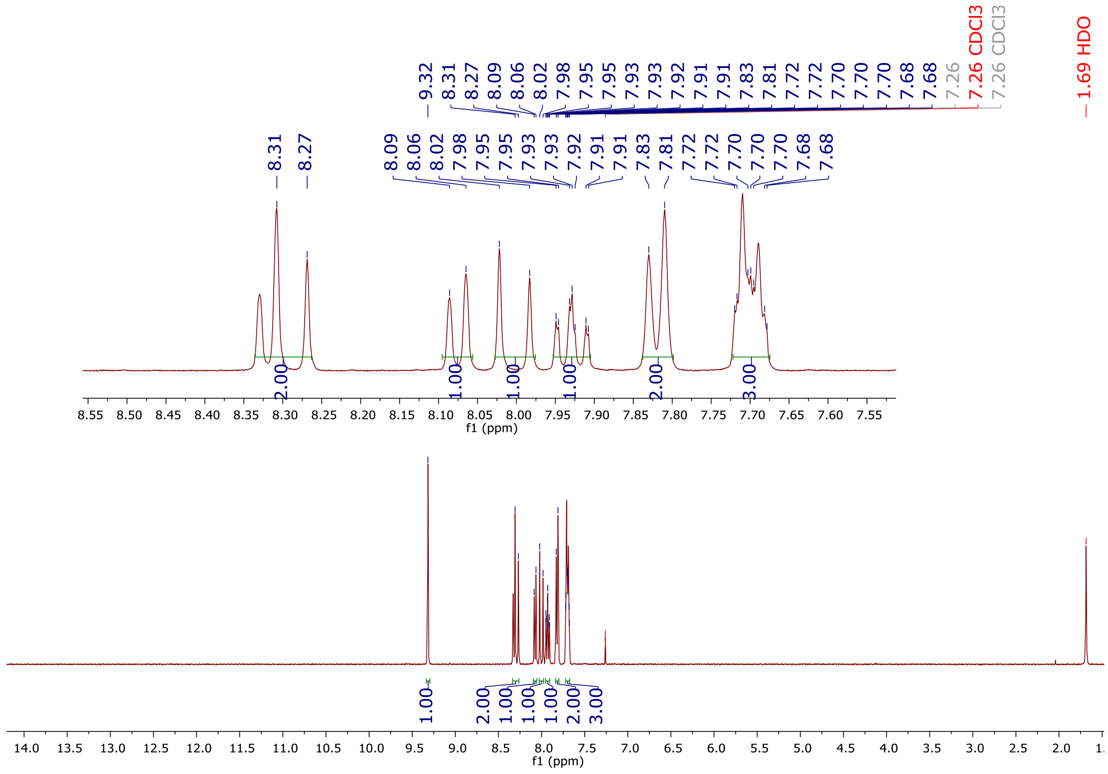

^{13}C NMR spectrum of 5f

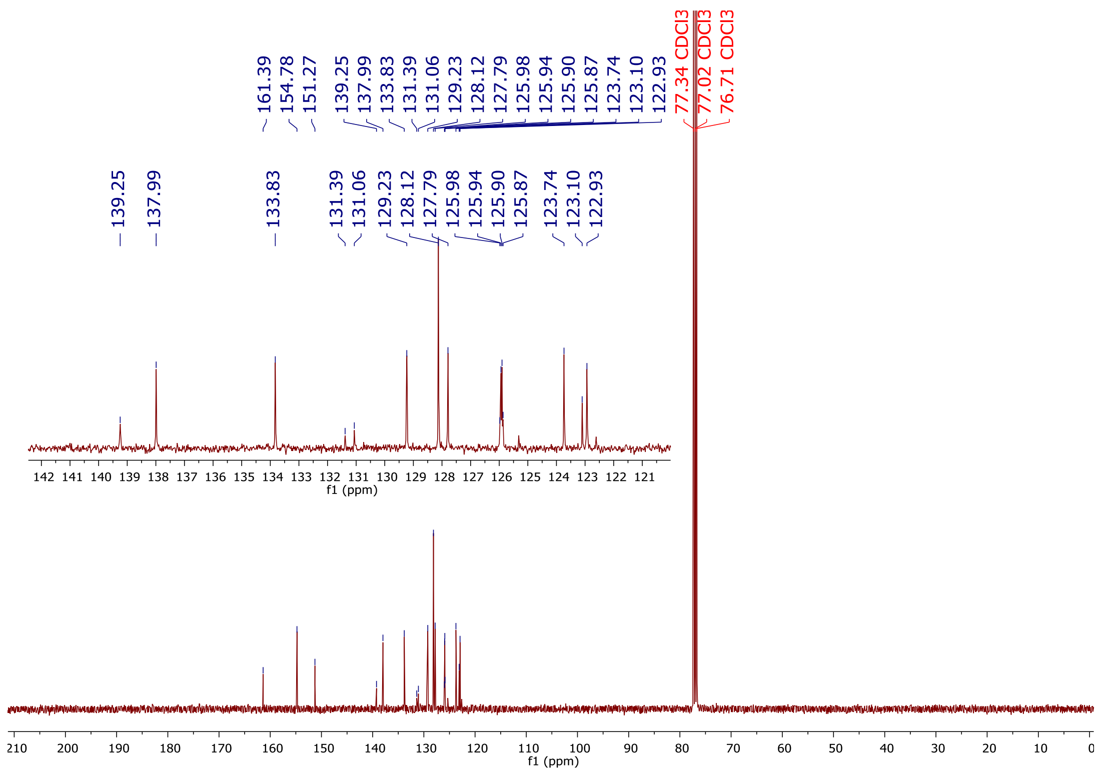

HRMS spectrum of 5f

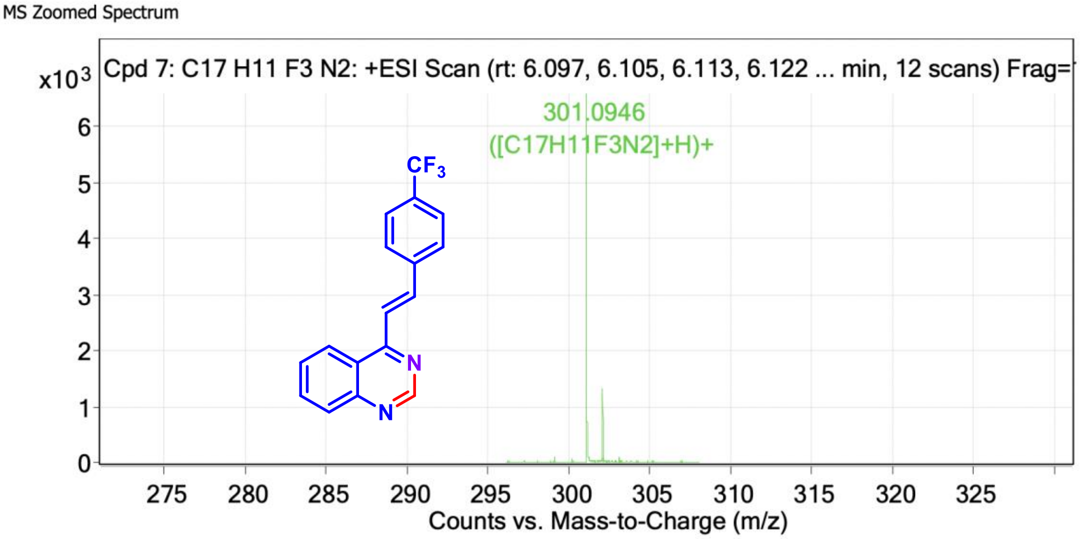

MS Spectrum Peak List

m/z	Calc *m/z*	Diff(ppm)	z	Abund	Formula	Ion
301.0946	301.0947	0.32	1	6880.04	C17H11F3N2	(M+H)+
302.0981	302.0978	-0.69	1	1419.71	C17H11F3N2	(M+H)+
303.1053	303.101	-14.22	1	70.38	C17H11F3N2	(M+H)+

--- End Of Report ---

(*E*)–4–(2,6–Dichlorostyryl)quinazoline (5g)

^{1}H NMR spectrum of 5g

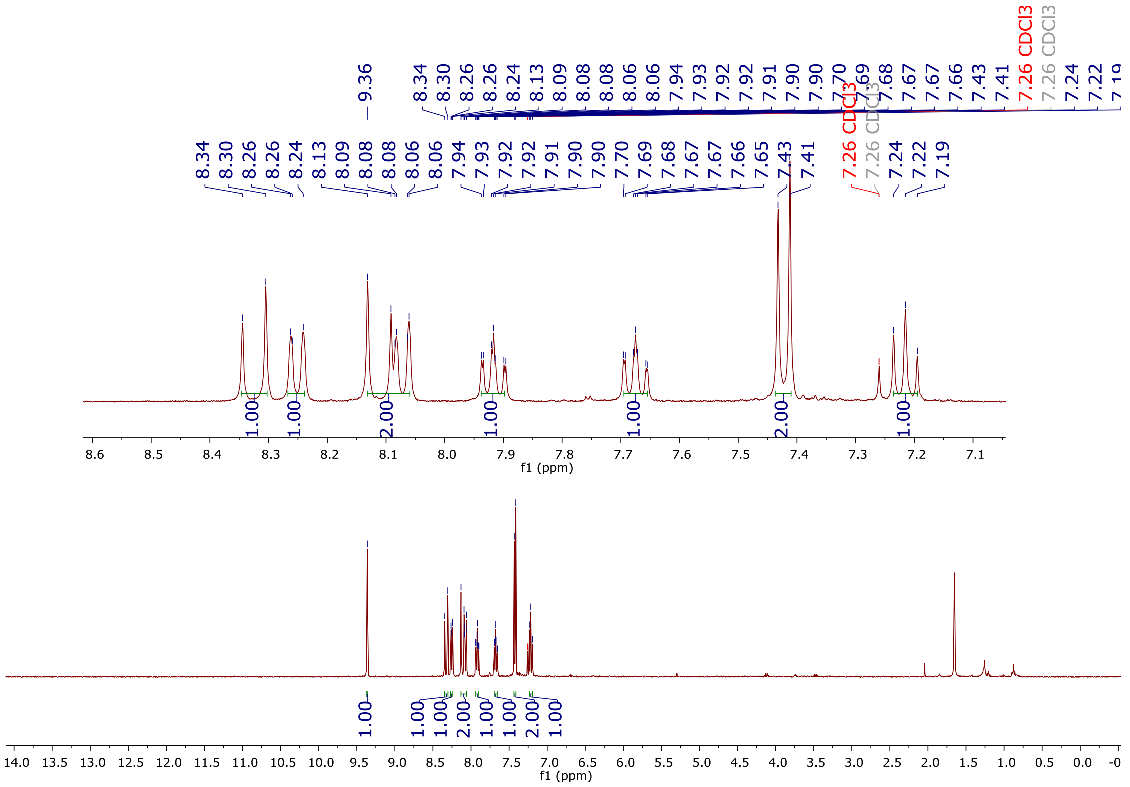

^{13}C NMR spectrum of 5g

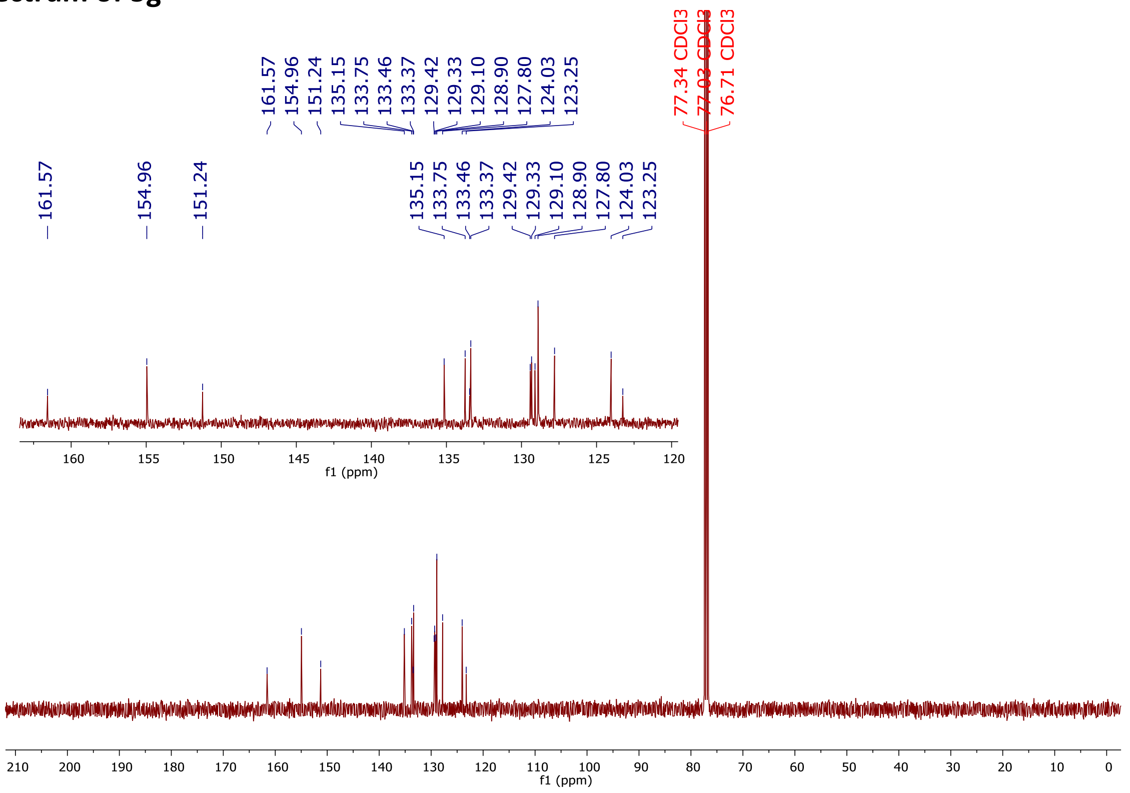

HRMS spectrum of 5g

MS Zoomed Spectrum

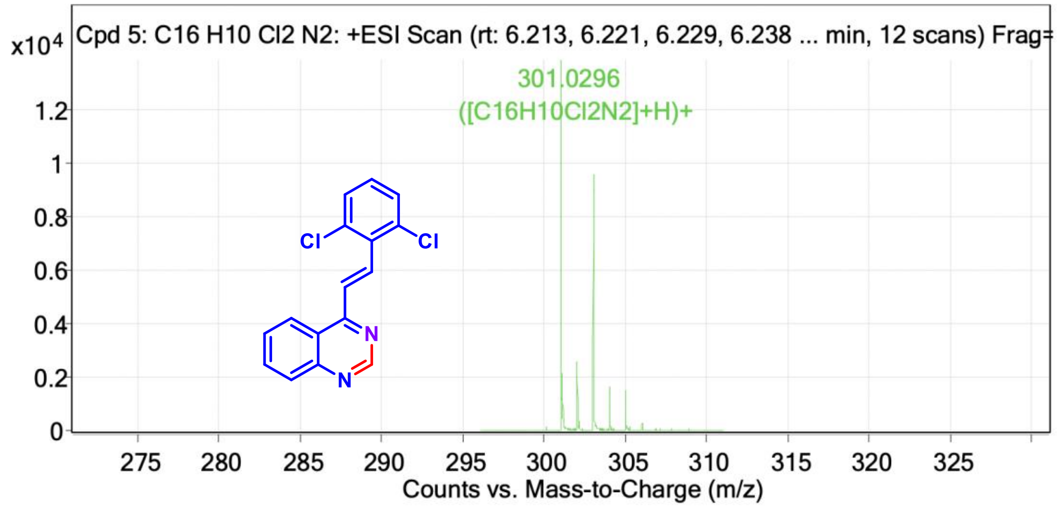

MS Spectrum Peak List

m/z	Calc *m/z*	Diff(ppm)	z	Abund	Formula	Ion
301.0296	301.0294	-0.9	1	14337.67	C16H10Cl2N2	(M+H)+
302.0319	302.0325	2.04	1	2640.04	C16H10Cl2N2	(M+H)+
303.0267	303.0266	-0.25	1	9794.85	C16H10Cl2N2	(M+H)+
304.03	304.0296	-1.33	1	1674	C16H10Cl2N2	(M+H)+
305.0245	305.0243	-0.6	1	1580.69	C16H10Cl2N2	(M+H)+
306.0279	306.0269	-3.43	1	289.01	C16H10Cl2N2	(M+H)+

--- End Of Report ---

(*E*)–4–(2–Chloro–6–fluorostyryl)quinazoline (5h)

^{1}H NMR spectrum of 5h

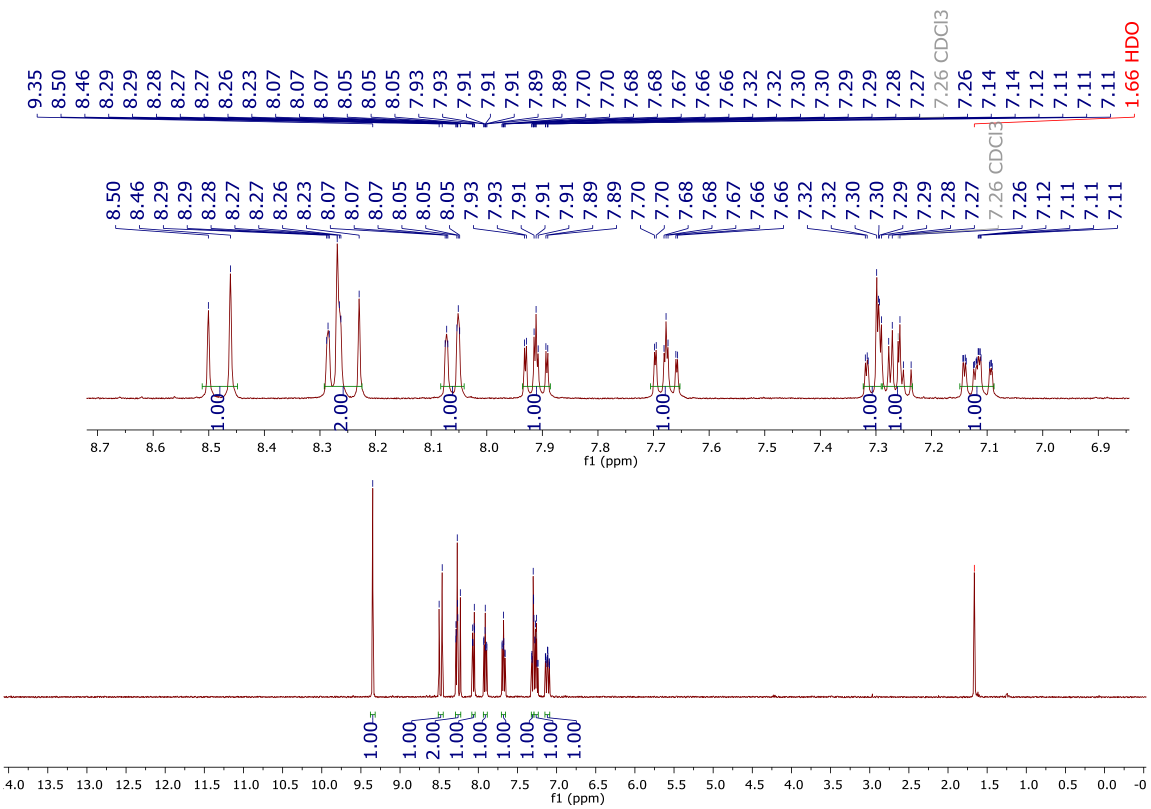

^{13}C NMR spectrum of 5h

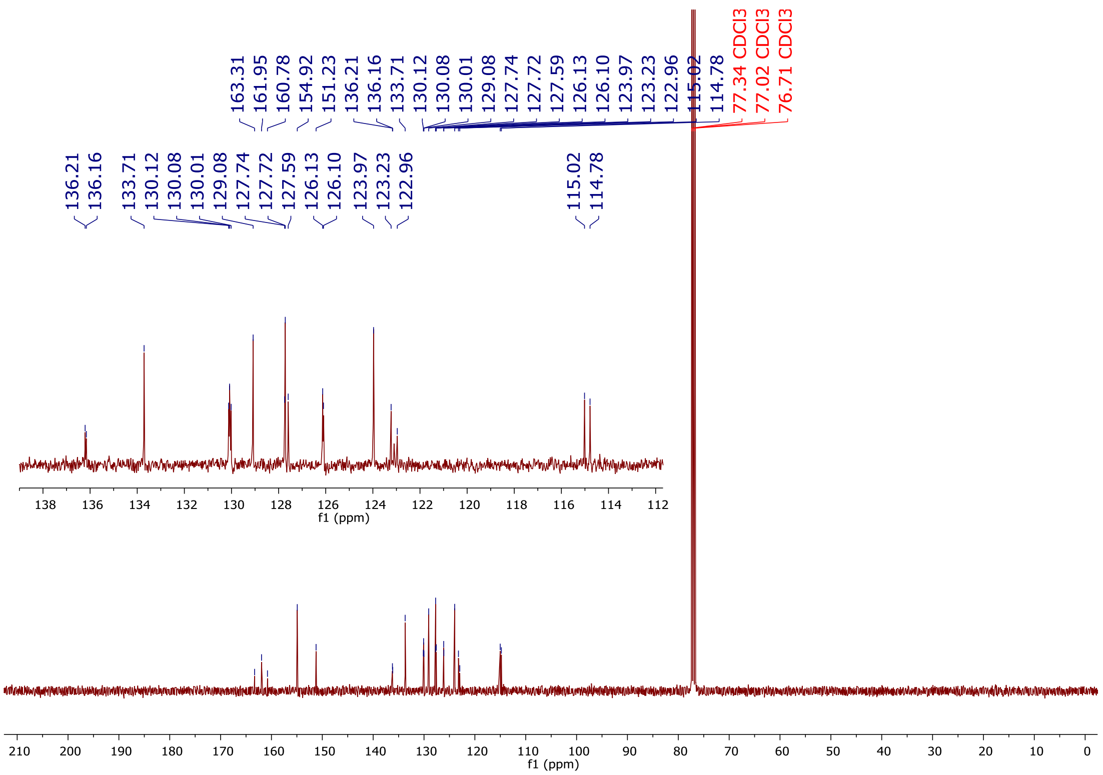

HRMS spectrum of 5h

MS Zoomed Spectrum

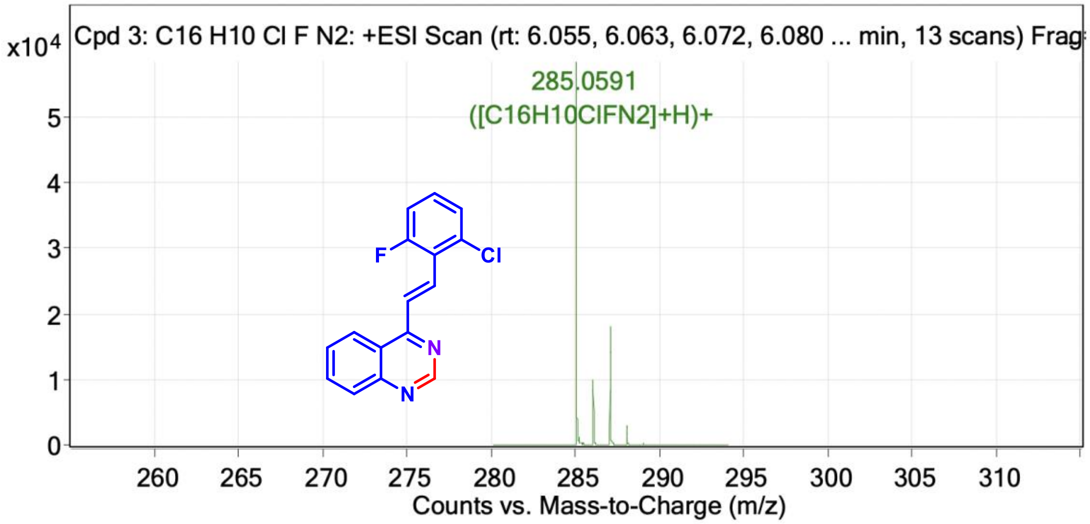

MS Spectrum Peak List

m/z	Calc *m/z*	Diff(ppm)	z	Abund	Formula	Ion
285.0591	285.0589	-0.75	1	58451.52	C16H10ClFN2	(M+H)+
286.0624	286.0621	-1.06	1	10025.93	C16H10ClFN2	(M+H)+
287.0564	287.0564	-0.06	1	18078.54	C16H10ClFN2	(M+H)+
288.0597	288.0592	-1.48	1	3233.74	C16H10ClFN2	(M+H)+
289.062	289.0623	0.81	1	259.28	C16H10ClFN2	(M+H)+

--- End Of Report ---

(*E*)–4–(3,4,5–Trimethoxystyryl)quinazoline (5i)

^{1}H NMR spectrum of 5i

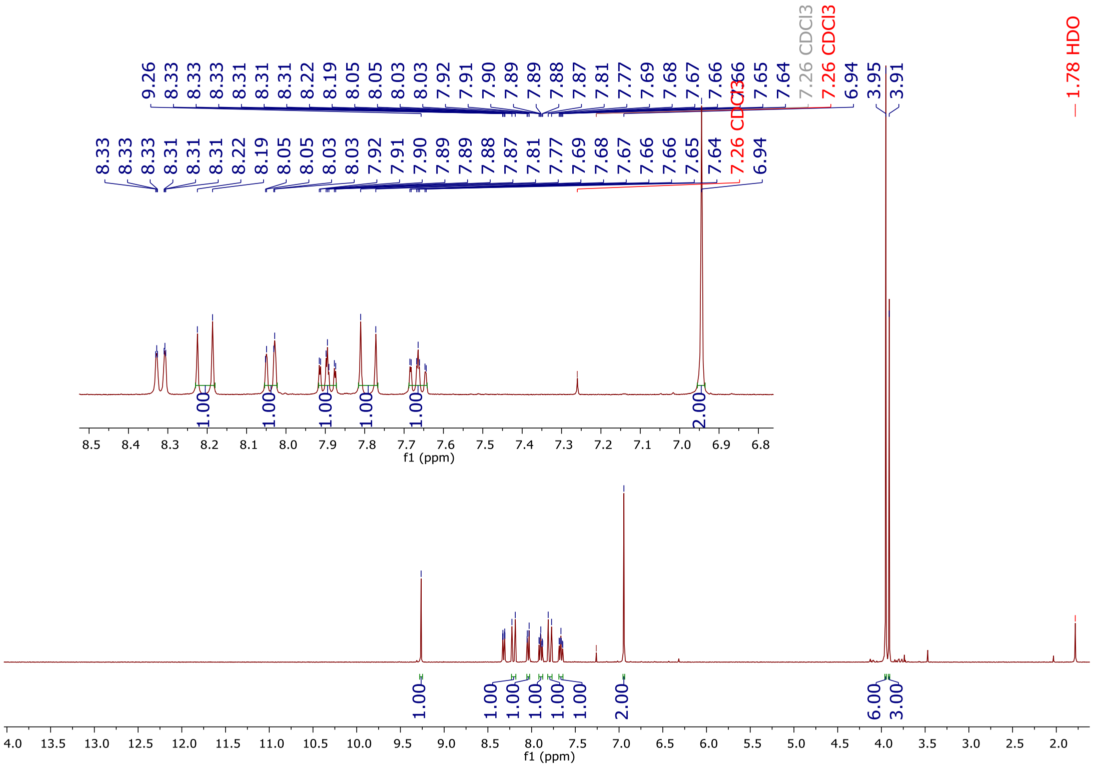

^{13}C NMR spectrum of 5i

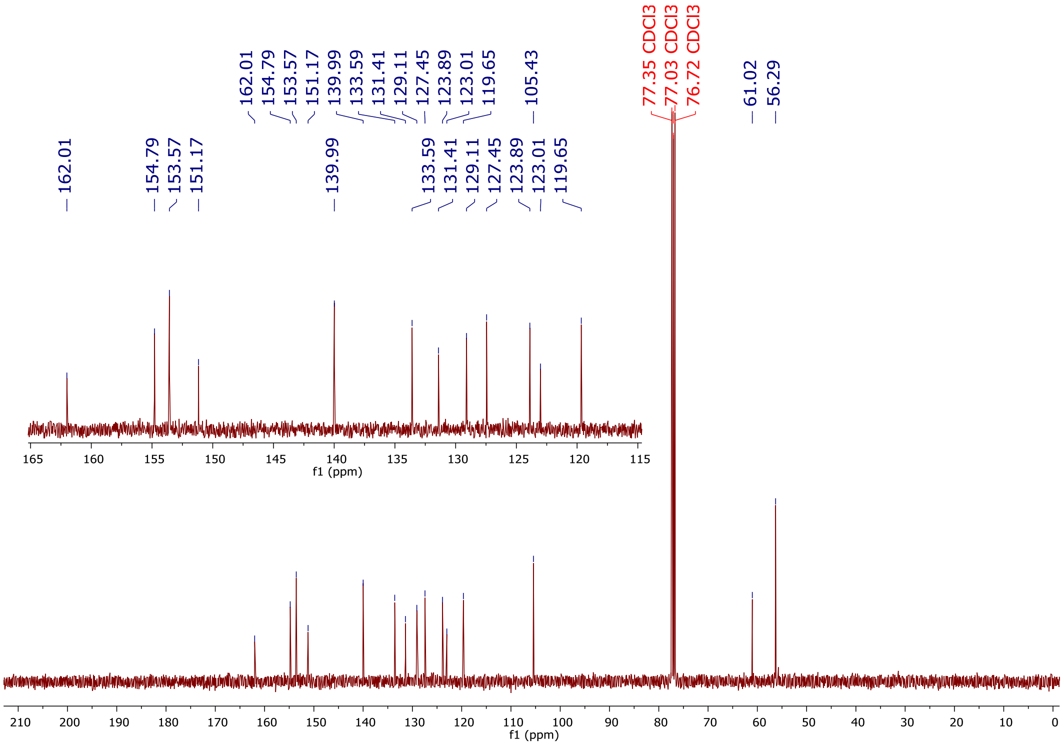

Chromatogram and HRMS spectrum of 5i

Compound Chromatograms

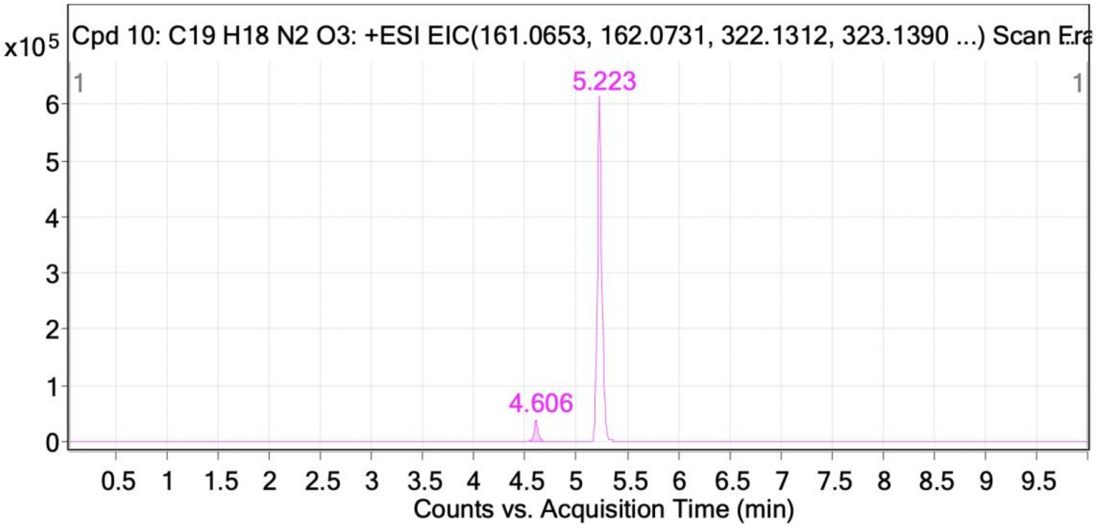

MS Zoomed Spectrum

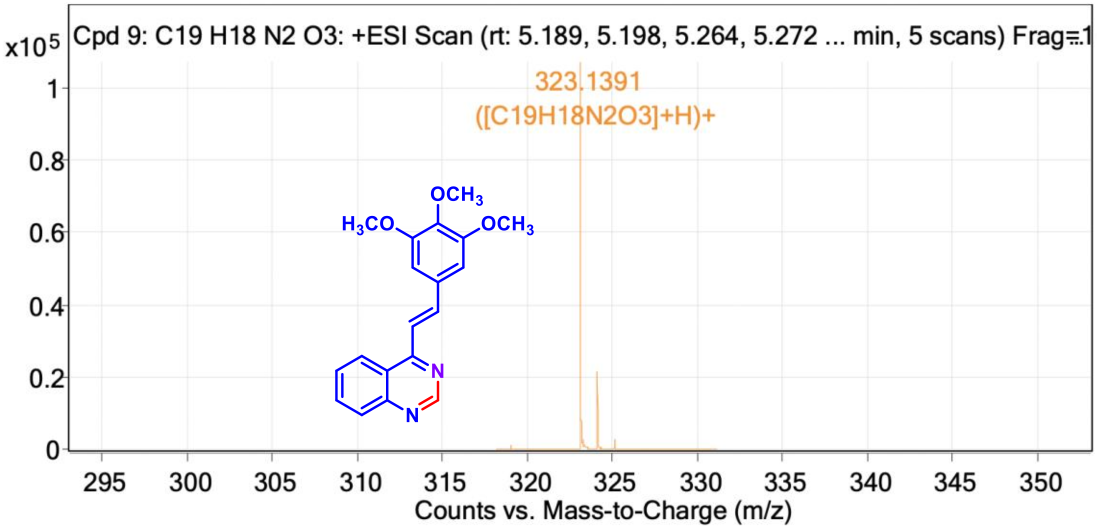

MS Spectrum Peak List

m/z	Calc *m/z*	Diff(ppm)	z	Abund	Formula	Ion
323.1391	323.139	-0.34	1	111646	C19H18N2O3	(M+H)+
324.1423	324.1422	-0.36	1	21860.79	C19H18N2O3	(M+H)+
325.145	325.1449	-0.38	1	2756.37	C19H18N2O3	(M+H)+
326.1483	326.1475	-2.3	1	274.53	C19H18N2O3	(M+H)+

--- End Of Report ---

(*E*)–4–(2–(Thiophen–2–yl)vinyl)quinazoline (5j)

^{1}H NMR spectrum of 5j

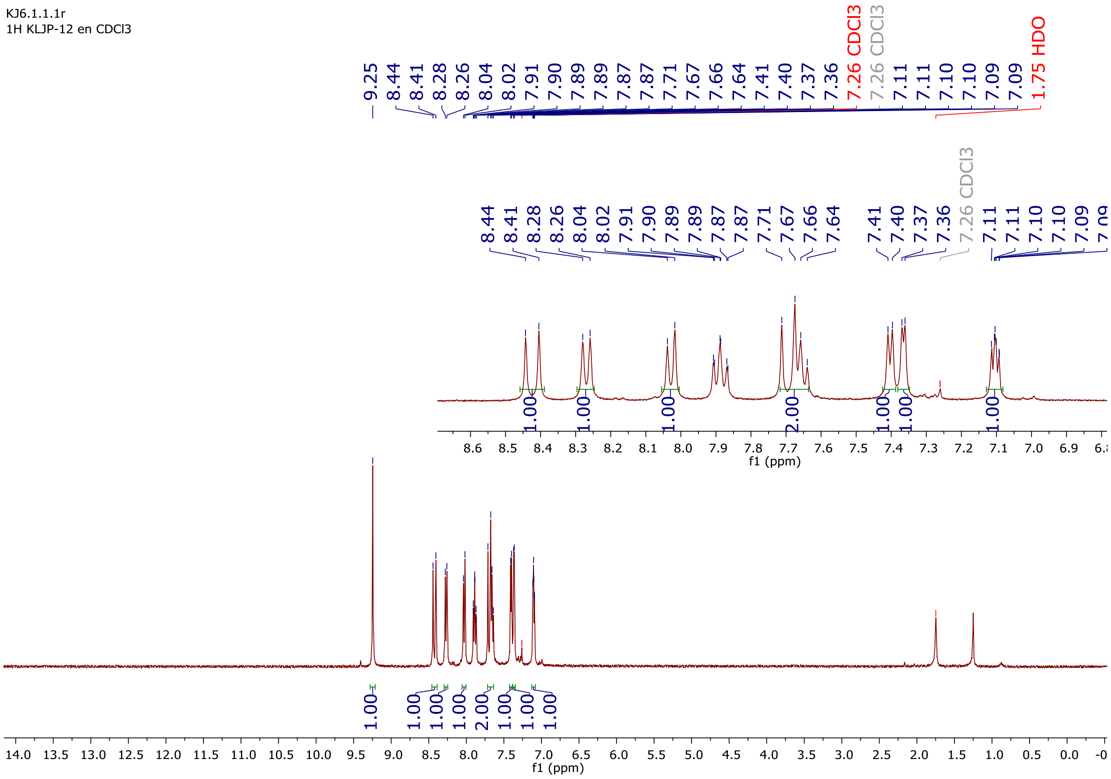

^{13}C NMR spectrum of 5j

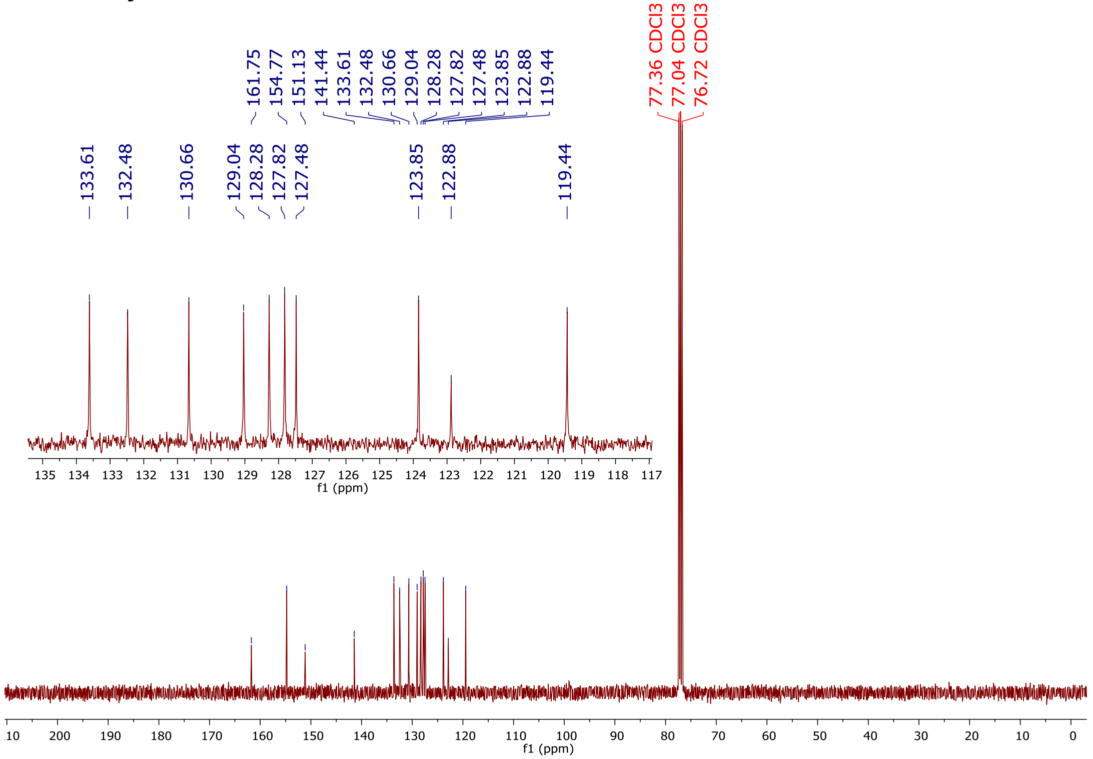

HRMS spectrum of 5j

MS Zoomed Spectrum

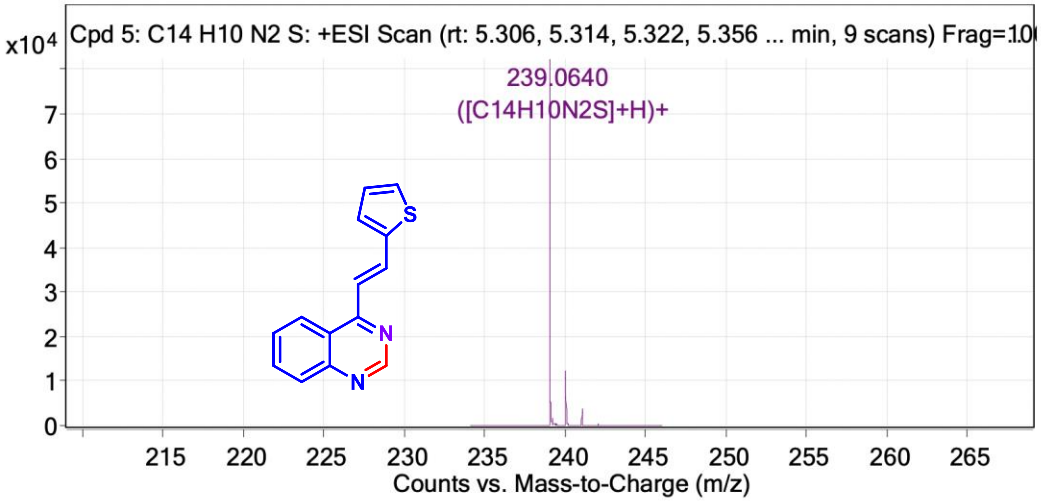

MS Spectrum Peak List

m/z	Calc *m/z*	Diff(ppm)	z	Abund	Formula	Ion
239.064	239.0637	-1.24	1	82875.63	C14H10N2S	(M+H)+
240.0671	240.0667	-1.88	1	12353.96	C14H10N2S	(M+H)+
241.0622	241.0618	-1.4	1	3852.07	C14H10N2S	(M+H)+

--- End Of Report ---

Table 1S Growth inhibitory percentage (GI%) of compounds **3** over all tested *in vitro* tumor cell lines.[a]

Cancer cell Lines Panel	Growth Inhibition Percentage (GI %) of compounds 3														
	**3a**[b]	**3b**[c]	**3c**[d]	**3d**[e]	**3e**[f]	**3f**[g]	**3g**[h]	**3h**[i]	**3i**[j]	**3j**[k]	**3k**[l]	**3l**[m]	**3m**[n]	**3n**[o]	**3o**[p]
**Leukemia**															
CCRF–CEM	4.26	17.37	9.78	0.00	14.58	30.15	29.80	0.00	3.75	0.00	0.00	0.00	0.00	6.03	0.00
HL–60(TB)	9.25	24.15	12.11	0.00	17.11	29.18	30.19	12.96	0.00	0.00	0.00	9,9	0.00	0.00	0.00
K–562	8,86	11.02	12.18	5.38	39.97	31.10	35.38	12.28	11.37	3.60	0.00	0.00	1.89	9.42	1.69
MOLT–4	14.59	33.11	6.29	7.85	37.85	42.23	41.90	8.32	17.25	0.00	14.17	0.00	3.38	7.25	0.00
RPMI–8226	0.00	1.27	13.42	4.10	28.47	19.90	35.29	12.12	1.24	9.32	0.00	0.00	0.00	0.00	0.00
SR	20.48	7.77	NT	0.00	33.83	34.20	46.53	26.31	4.90	5.91	10.93	0.01	0.00	10.56	0.00
**Non-Small Cell Lung Cancer**															
A549/ATCC	12.3	11.75	18.30	0.00	24.07	40.18	45.63	10.84	0.00	9.54	9.42	0.00	0.00	0.00	15.17
EKVX	0.00	9.45	15.83	12.90	9.16	39.19	48.72	2.68	8.19	0.00	0.00	10.29	7.77	8.88	0.40
HOP–62	0.00	0.00	67.96	4.01	0.00	43.23	47.07	0.00	0.00	0.00	0.00	3.95	0.00	0.00	0.00
HOP–92	0.00	0.00	57.59	0.00	0.00	59.98	61.72	0.00	0.00	0.00	0.00	0.00	0.00	4.57	0.00
NCI–H226	0.00	0.00	78.69	3.40	2.97	45.35	59.23	6.71	0.03	5.15	6.31	0.00	2.85	0.00	0.00
NCI–H23	6.72	0.54	38.62	10.41	11.12	41.24	43.21	4.48	3.65	3.97	0.00	0.00	5.16	0.00	2.76
NCI–H322M	3.46	0.00	26.95	1.18	5.15	14.34	19.36	0.00	6.34	0.00	0.00	6.13	6.9	0.00	0.00
NCI–H460	1.65	4.59	50.95	0.00	17.51	40.01	40.08	0.00	0.00	0.00	0.00	0.00	0.00	0.00	0.00
NCI–H522	8.48	11.58	36.84	4.43	20.15	45.13	46.42	29.67	4.60	0.00	28.46	4.16	3.35	–26.93	25.97
**Colon Cancer**															
COLO 205	0.00	0.00	0.00	21.15	15.06	40.76	43.16	0.21	24.80	0.00	0.00	0.00	0.00	0.00	0.00
HCC–2998	0.00	0.00	9.87	0.00	0.23	18.92	17.41	0.00	0.00	0.00	0.00	4.56	0.00	0.00	0.00
HCT–116	5.37	13.30	63.36	5.89	23.24	40.23	44.03	11.01	2.95	0.28	10.14	6.03	0.00	24.56	6.60
HCT–15	15.02	12.23	11.69	20.90	42.31	40.34	38.24	0.08	9.73	0.00	0.00	0.00	4.66	16.53	0.00
HT29	13.68	14.94	26.33	0.00	20.35	43.34	41.23	13.51	0.00	11.24	19.46	0.00	0.00	6.07	16.85
KM12	7.22	2.16	16.64	13.43	39.39	10.23	12.41	9.25	6.50	2.25	1.40	0.00	3.81	2.73	0.00
SW–620	3.46	0.80	8.43	0.05	0.00	21.56	22.38	7.20	0.00	0.00	0.00	0.00	0.06	4.34	0.00
**CNS Cancer**															

SF–268	3.23	0.65	48.77	0.15	3.05	27.45	29.91	2.44	2.24	0.00	6.99	0.00	4.63	0.00	0.00
SF–295	0.00	5.25	85.18	0.00	0.00	45.17	49.81	0.00	0.00	0.00	0.00	0.00	0.00	0.00	0.00
SF–539	2.33	0.00	73.02	1.54	2.40	30.14	31.57	6.58	0.00	6.79	5.05	4.13	1.79	1.38	1.21
SNB–19	0.00	0.00	76.78	0.00	5.14	30.24	29.15	0.00	1.15	3.64	0.00	0.00	0.25	0.00	0.00
SNB–75	NT	5.37	98.89	NT	NT	NT	NT	13.93	NT	10.47	13.02	0.00	NT	0.00	12.36
U251	0.00	0.00	54.18	0.00	10.21	41.78	34.13	3.48	0.00	0.00	0.00	0.00	0.00	0.00	0.00
**Melanoma**															
LOX IMVI	5.25	12.5	22.14	15.28	27.11	50.13	56.80	7.10	12.81	3.30	8.48	11.82	8.03	8.81	7.73
MALME–3M	0.00	0.00	21.75	3.09	4.98	6.43	3.41	0.00	6.46	0.00	0.00	0.13	1.66	0.00	0.00
M14	0.00	6.42	20.02	0.00	30.38	10.10	11.09	9.23	7.69	4.05	4.21	3.10	0.00	12.25	0.00
MDA–MB–435	3.40	0.00	13.93	3.41	19.37	20.27	18.45	14.35	4.76	6.74	4.34	0.00	4.41	0.00	0.00
SK–MEL–2	NT	0.00	9.58	NT	NT	NT	NT	12.36	NT	11.70	9.35	0.00	NT	0.00	2.67
SK–MEL–28	0.00	0.00	8.65	0.00	3.8	11.70	11.68	0.00	1.56	0.00	0.00	0.00	0.00	0.00	0.00
SK–MEL–5	0.16	4.80	42.77	2,95	87.92	16.98	13.05	1.23	0.00	4.93	7.34	0.00	0.78	0.00	3.66
UACC–257	0.00	0.00	0.69	0.00	0.00	23.48	22.57	5.74	0.00	11.96	5.86	0.00	0.00	0.00	5.42
UACC–62	13.30	13.11	29.85	12.33	15.97	50.50	50.45	NT	24.50	NT	NT	13.24	20.43	20.31	NT
**Ovarian Cancer**															
IGROV1	0.00	0.00	34.47	10.41	0.00	21.20	20.93	0.00	1.91	0.00	0.00	3.48	3.16	0.00	0.00
OVCAR–3	NT	0.00	11.99	NT	NT	17.21	19.01	0.00	NT	0.00	0.00	0.00	NT	0.00	0.00
OVCAR–4	7.55	0.61	74.82	0.00	9.67	35.98	39.85	9.30	7.41	7.88	1.08	0.00	1.06	0.00	0.00
OVCAR–5	0.00	0.00	2.58	0.00	0.00	5.37	5.36	0.00	0.26	0.00	11.55	6.69	4.21	0.00	0.00
OVCAR–8	0.00	0.00	84.52	0.00	19.29	4.89	4.79	2.38	0.00	0,38	0.00	0.00	0.00	0.00	0.00
NCI/ADR–RES	0.00	0.20	60.21	0.00	34.03	44.35	40.17	0.00	0.00	0.00	1.53	0.00	0.00	1.59	1.63
SK–OV–3	0.00	0.00	83.26	0.00	0.00	10.56	13.33	0.00	0.39	0.00	0.00	0.00	0.00	0.00	0.00
**Renal Cancer**															
786–0	8.7	13.92	–6.57	0.00	8.21	16.45	16.35	1.37	0.00	0.00	6.32	5.79	0.00	7.53	0.00
A498	0.00	0.00	34.47	0.00	0.00	5.90	0.00	0.00	0.00	0.00	0.00	NT	0.00	NT	0.00
ACHN	0.00	0.00	64.71	9.45	10.22	40.19	44.84	2.52	6.56	0.00	0.00	0.00	0.00	0.00	0.57
CAKI–1	20.58	7.98	45.96	28.61	27.51	30.45	36.44	27.36	30.17	23.47	27.71	21.01	18.26	30.61	19.00
RXF 393	0.00	0.00	96.43	0.00	11.86	40.10	40.26	0.00	0.00	0.00	0.00	0.00	0.00	0.00	0.00

SN12C	3.82	4.15	22.47	13.35	13.77	34.67	30.75	10.96	12.68	13.71	17.94	0.01	6.19	3.11	13.76
TK–10	0.00	0.95	33.08	0.00	0.00	20.13	18.54	0.00	0.00	0.00	4.39	0.00	0.00	0.00	0.00
UO–31	4.60	10.03	NT	30.74	11.21	NT	NT	14.68	18.79	6.16	10.52	8.09	14.52	8.17	1.07
**Prostate Cancer**															
PC–3	0.00	0.00	22.44	0.00	42.18	29.66	30.06	0.00	0.00	0.00	0.00	0.00	0.00	2.44	0.00
DU–145	0.00	0.00	8.94	0.00	8.65	34.12	29.48	0.32	0.00	0.00	1.11	0.00	0.00	0.00	0.00
**Breast Cancer**															
MCF7	11.66	13.52	37.9	32.70	18.75	67.90	60.98	15.68	24.78	18.22	7.29	11.03	21.89	23.16	21.20
MDA–MB–231/ATCC	0.00	0.00	NT	36,83	17.85	NT	NT	1.27	18.73	5.58	3.78	7.16	9.42	0.82	6.92
HS 578T	0.00	0.95	79.18	0.00	0.00	36.31	30.90	0.00	0.00	0.00	0.00	0.00	0.00	0.00	0.00
BT–549	0.00	0.00	26.07	0.00	8.78	20.20	17.07	0.00	0.00	0.00	0.00	1.94	0.00	0.00	0.00
T–47D	23.46	23.64	21.91	31.13	44.99	45.34	49.54	9.45	19.65	13.63	9.79	8.70	13.83	20.32	0.89
MDA–MB–468	7.76	8.91	29.84	1.26	28.07	80.23	81.29	14.12	5.95	0.00	0.00	15.44	1.62	14.12	0.00
**$GI_{average}$ (%)**	0.10	1.71	37.88	1.64	14.20	30.18	32.73	2.24	2.06	0.00	0.00	0.00	0.00	0.78	0.00

[a] Data obtained from NCIs *in vitro* disease-oriented human tumor cell screen; [b] NSC 840320; [c] NSC 840759; [d] NSC 838452; [e] NSC 840324; [f] NSC 840325; [g] NSC 846822; [h] NSC 838911; [i] NSC 846819; [j] NSC 840323; [k] NSC 846825; [l] NSC 846820; [m] NSC 840763; [n] NSC 840322; [o] NSC 840764; [p] NSC 846824; NT, Not tested.

Table 2S Growth inhibitory percentage (GI%) of compounds **5** over all tested *in vitro* tumor cell lines.[a]

**Cancer cell lines panel**[a]	**Growth Inhibition Percentage (GI %) of compounds 5**									
	**5a**[b]	**5b**[c]	**5c**[d]	**5d**[e]	**5e**[f]	**5f**[g]	**5g**[h]	**5h**[i]	**5i**[j]	**5j**[k]
**Leukemia**										
CCRF–CEM	14.56	69.76	78.02	89.16	67.84	28.93	35.59	59.20	58.08	0.00
HL–60(TB)	89.33	84.60	–8.73	–27.57	75.07	61.45	39.74	58.02	79.50	14.98
K–562	59.51	92.99	88.85	98,2	82.02	52.30	44.62	71.54	86.51	21.53
MOLT–4	64.88	65.58	72.39	–1.65	41.03	43.95	42.14	47.67	73.46	10.54
RPMI–8226	61,89	66.39	68.92	83.89	72.81	52.30	36.27	73.24	64.30	33.14
SR	38.11	NT	NT	95.4	70.89	57.53	55.01	NT	92.94	18.35
**Non-Small Cell Lung Cancer**										
A549/ATCC	34.77	47.30	72.00	89.94	68.19	35.57	31.03	26.11	42.32	23.72
EKVX	42.48	56.18	59.54	64.39	60.71	52.44	48.41	45.12	67.93	44.52
HOP–62	3.19	27.08	76.42	70.17	31.95	21.71	23.94	16.26	41.12	8.66
HOP–92	31.67	40.12	46.08	80.85	45.41	50.08	0.00	18.95	67.86	3.20
NCl–H226	26.22	32.94	71.37	–43.90	43.46	36.84	0.00	22.63	57.15	0.00
NCl–H23	13.97	49.48	96.21	–14.17	54.07	37.41	25.91	28.64	68.09	1.26
NCl–H322M	3.97	43.98	87.24	86.38	56.89	23.93	14.35	22.63	41.27	8.80
NCl–H460	40.03	43.53	74.56	81.64	65.22	17.44	22.47	22.76	47.67	3.05
NCl–H522	49.84	60.70	61.45	–16.27	77.34	44.01	43.66	35.31	78.42	39.50
**Colon cancer**										
COLO 205	19.5	22.21	–57.00	–71.21	46.50	53.61	10.71	0.00	47.67	0.00
HCC–2998	25.46	11.59	67.11	99.96	87.64	13.31	0.00	0.00	36.30	0.00
HCT–116	61.27	65.71	96.31	78.85	65.08	40.94	40.40	42.32	60.91	9.33
HCT–15	47.4	76.62	92.93	76.81	76.76	50.57	31.32	44.73	65.83	19.87
HT29	54.69	54.85	75.91	90.60	60.90	24.03	17.35	18.29	54.42	9.99
KM12	37.4	68.11	92.35	89.92	88.36	19.82	33.99	25.35	21.52	10.28
SW–620	27.5	71.54	99.58	96.99	79.37	31.36	16.08	24.37	58.07	0.00
**CNS cancer**										
SF–268	18.38	32.67	42.76	66.16	59.33	13.56	25.58	30.08	40.73	20.48

SF–295	22.69	34.02	52.23	78.03	39.18	35.64	0.00	21.03	24.75	0.00
SF–539	5.47	50.60	88.86	–3.62	60.36	12.02	15.88	15.90	50.84	15.19
SNB–19	14.27	37.88	51.32	86.17	47.99	28.25	0.00	18.48	34.06	17.10
SNB–75	0.00	61.96	NT	–13.87	88.14	NT	50.29	28.49	NT	35.61
U251	23.24	63.40	77.50	94.69	69.30	48.61	21,2	28.61	59.70	15.17
**Melanoma**										
LOX IMVI	19.37	54.72	81.63	–7.92	65.36	57.55	36.65	43.41	63.37	21.41
MALME–3M	0.00	33.46	39.36	36.25	2.60	12.02	0.00	13.52	44.25	0.00
M14	15.15	46.10	77.55	65.59	33.80	25.42	16.02	25.82	33.29	4.98
MDA–MB–435	20.12	78.45	84.31	81.73	68.48	17.61	32.45	26.26	54.74	2.97
SK–MEL–2	20.01	59.93	NT	–38.41	53.75	NT	21.42	20.06	NT	2.08
SK–MEL–28	10.59	20.44	45.31	45.11	12.00	6.10	3.44	0.00	20.06	0.00
SK–MEL–5	27.65	55.02	43.33	78.99	37.38	11.49	16.19	25.17	27.61	9.76
UACC–257	8.53	30.89	41.90	49.34	22.02	30.42	17.21	20.52	49.97	13.85
UACC–62	30.53	57.70	82.74	–11.68	NT	50.34	45.95	43.82	57.52	NT
**Ovarian cancer**										
IGROV1	13.48	25.28	55.31	69.89	88.49	18.90	6.48	5.33	25.79	29.27
OVCAR–3	15.42	62.91	NT	–21.85	91.46	32.35	20.38	36.23	52.97	5.42
OVCAR–4	27.06	23.17	59.61	80.28	62.11	22.67	28.44	23.82	55.27	8.01
OVCAR–5	7.64	20.38	92.98	88.94	59.48	8.27	13.02	0.00	20.91	0.00
OVCAR–8	21.65	44.54	88.44	–7.54	50.28	22.78	27.6	25.41	42.40	7.04
NCI/ADR–RES	NT	68.54	–6.38	–35.29	72.26	28.97	NT	33.75	70.50	11.57
SK–OV–3	23.41	38.53	49.52	64.64	35.10	8.58	84.77	14.23	51.74	1.17
**Renal cancer**										
786–0	0.00	33.58	64.96	75.05	35.42	8.94	6.56	8.81	29.80	0.00
A498	20.58	0.00	0.00	45.33	0.00	0.00	0.00	0.00	10.86	0.00
ACHN	20.56	50.67	73.70	87.54	62.53	45.06	23.08	25.34	58.15	17.14
CAKI–1	38.94	41.92	65.27	93.98	84.12	27.44	43.95	15.78	47.26	29.88
RXF 393	1.70	46.05	83.45	87.85	40.53	4.52	37.83	0.82	77.80	0.00
SN12C	29.27	48.04	78.61	93.89	66.87	44.71	42.46	15.15	52.67	20.59

TK–10	0.00	0.00	22.79	85.35	70.75	0.78	0.00	0.00	44.77	0.00
UO–31	48.32	NT	74.00	82.20	46.29	NT	24.19	NT	NT	33.04
**Prostate cancer**										
PC–3	65.43	54.53	93.58	65.11	51.66	47.00	12.15	34.43	47.51	3.18
DU–145	0.00	54.11	85.25	85.19	86.45	17.14	31.77	35.49	55.69	16.2
**Breast cancer**										
MCF7	49.03	70.23	89.16	82.44	95.75	64.88	50.47	63.28	70.27	48.66
MDA–MB–231/ATCC	18.49	NT	92.85	–11.52	43.26	NT	40.69	NT	50.39	18.86
HS 578T	12.91	38.63	25.55	84.10	51.7	16.66	32.29	21.79	73.51	11.92
BT–549	21.68	83.29	70.40	90.46	71.71	17.21	24.52	29.80	57.43	6.07
T–47D	69.07	69.74	77.45	98.98	95.40	57.55	91.94	51.17	NT	65.78
MDA–MB–468	16.87	–38.00	–20.52	–39.18	–35.82	49.38	75.66	87.21	0.00	39.96
**$GI_{average}$ (%)**	26.00	50.26	73.52	91.37	60.48	30.19	26.20	25.61	53.44	10,69

[a] Data obtained from NCIs *in vitro* disease-oriented human tumor cell screen; [b] NSC 839195; [c] NSC 838449; [d] NSC 840315; [e] NSC 847835; [f] NSC 846817; [g] NSC 838910; [h] NSC 839196; [i] NSC 838448; [j] NSC 838908; [k] NSC 846816; NT, Not tested.
